# Heat Shock Protein 20 Gene Superfamilies in Red Algae: Evolutionary and Functional Diversities

**DOI:** 10.3389/fpls.2022.817852

**Published:** 2022-03-16

**Authors:** Tian Gao, Zhaolan Mo, Lei Tang, Xinzi Yu, Guoying Du, Yunxiang Mao

**Affiliations:** ^1^Key Laboratory of Marine Genetics and Breeding (Ministry of Education), College of Marine Life Sciences, Ocean University of China, Qingdao, China; ^2^Key Laboratory of Tropical Aquatic Germplasm of Hainan Province, Sanya Oceanographic Institution, Ocean University of China, Sanya, China; ^3^Key Laboratory of Utilization and Conservation of Tropical Marine Bioresource (Ministry of Education), College of Fisheries and Life Science, Hainan Tropical Ocean University, Sanya, China; ^4^Yazhou Bay Innovation Research Institute, Hainan Tropical Ocean University, Sanya, China; ^5^Key Laboratory for Conservation and Utilization of Tropical Marine Fishery Resources of Hainan Province, Hainan Tropical Ocean University, Sanya, China

**Keywords:** heat shock protein 20, red algae, phylogenetic analysis, expression profile, abiotic and biotic stresses, growth and development

## Abstract

Heat shock protein 20 (*Hsp20*) genes play important roles in plant growth, development, and response to environmental stress. However, the *Hsp20* gene family has not yet been systematically investigated, and its function in red algae (Rhodophyta) remains poorly understood. Herein, we characterized *Hsp20* gene families in red algae by studying gene structure, conserved motifs, phylogenetic relationships, chromosome location, gene duplication, *cis-*regulatory elements, and expression profiles. In this study, 97 *Hsp20* genes were identified using bioinformatic methods and classified into 13 subfamilies based on phylogenetic relationships. Phylogenetic analysis revealed that *Hsp20* genes might have a polyphyletic origin and a complex evolutionary pattern. Gene structure analysis revealed that most *Hsp20* genes possessed no introns, and all *Hsp20* genes contained a conserved α-crystalline domain in the C-terminal region. Conserved motif analysis revealed that *Hsp20* genes belonging to the same subfamily shared similar motifs. Gene duplication analysis demonstrated that tandem and segmental duplication events occurred in these gene families. Additionally, these gene families in red algae might have experienced strong purifying selection pressure during evolution, and *Hsp20* genes in *Pyropia yezoensis*, *Pyropia haitanensis*, and *Porphyra umbilicalis* were highly evolutionarily conserved. The *cis-*elements of phytohormone-, light-, stress-responsive, and development-related were identified in the red algal *Hsp20* gene promoter sequences. Finally, using *Py. yezoensis*, as a representative of red algae, the *Hsp20* gene expression profile was explored. Based on the RNA-seq data, *Py. yezoensis Hsp20* (*PyyHsp20)* genes were found to be involved in *Py. yezoensis* responses against abiotic and biotic stresses and exhibited diverse expression patterns. Moreover, *PyyHsp20* is involved in *Py. yezoensis* growth and development and revealed spatial and temporal expression patterns. These results provide comprehensive and valuable information on *Hsp20* gene families in red algae and lay a foundation for their functional characterization. In addition, our study provides new insights into the evolution of *Hsp20* gene families in red algae and will help understand the adaptability of red algae to diverse environments.

## Introduction

In recent years, heat has become a prominent abiotic stress factor owing to global warming. All organisms synthesize a set of proteins called heat shock proteins (HSPs) to combat heat stress (Lindquist and Craig, 1988). According to protein molecular weight and sequence homology, HSPs can be divided into five families: Hsp100s, Hsp90s, Hsp70s, Hsp60s, and Hsp20s, among which, Hsp20s are considered the most abundant and complex members (Vierling, 1991). In addition, the molecular size of Hsp20s ranges from 12 to 42 kDa, and thus they are called small HSPs. Hsp20s function as ATP-independent molecular chaperones to prevent proteins from irreversible aggregation and play a fundamental role in plant-acquired thermotolerance processes (Waters, 2013). Hsp20s mainly possess a highly conserved sequence of approximately 90 amino acid residues, called the α-crystalline domain (ACD) or the Hsp20 domain, in the C-terminal region, which is involved in substrate interactions (Waters and Vierling, 2020). In addition, the ACD is flanked by a variable N-terminal domain and a short C-terminal extension, which performs two different functions (Waters and Vierling, 2020).

Analyses of genome sequences from eukaryotic organisms have shown that *Hsp20* genes belong to a group of diverse and complex families. Plant Hsp20s can be divided into several subfamilies (CI-CVI, MTI, MTII, ER, CP, and PX) based on cellular location, sequence homology, and function (Waters and Vierling, 2020). CI-CVI subfamilies are localized in the cytoplasm and nucleus; MTI and MTII subfamilies are localized in the mitochondria; and the other three subfamilies (ER, CP, and PX) are localized in the endoplasmic reticulum, chloroplast, and peroxisome, respectively (Waters and Vierling, 2020). Previous studies have suggested that plant Hsp20s are not only induced by heat stress but also by a wide variety of other stresses, such as drought, salinity, osmotic stress, oxidative stress, and UV-B radiation (Wang et al., 2004; Waters, 2013). In general, stress-related Hsp20s are tightly repressed at low temperatures, whereas their overexpression might induce deleterious effects on plant growth and development (Sun et al., 2016; Bourgine and Guihur, 2021). Hsp20s plays an important role in plant immunity (Park and Seo, 2015). Currently, an increasing amount of data reveals a close correlation between Hsp20s accumulation and plant stress tolerance. In addition, Hsp20s are involved in plant growth and development, such as embryo development, somatic embryogenesis, seed germination, pollen development, and fruit maturation (Waters et al., 1996).

Red algae (Rhodophyta) are one of the most ancient groups of eukaryotic algae, with over 7,000 known species.^1^ They form a distinct photosynthetic eukaryotic lineage with primitive features, such as the absence of centrioles, flagella, and parenchyma; the presence of unstacked thylakoids; and phycobilisomes in the chloroplast (Lopez-Bautista, 2010). Red algae are classified into seven classes (Cyanidiophyceae, Bangiophyceae, Florideophyceae, Compsopogonophyceae, Porphyridiophyceae, Rhodellophyceae, and Stylonematophyceae) and exhibit a remarkable diversity in their habitats (e.g., hot springs, acidic sulfur fumes, fresh water, deep ocean, and intertidal zone) and morphology (e.g., unicells, filaments, leaf-shaped thallus, parenchymatous blades, single-cell-thick tubular form) (Yoon et al., 2010). Red algae comprise many commercially valuable species that can be used as food, pharmaceuticals, nutraceuticals, cosmetics, phycocolloids, and phyco supplements (e.g., soil additives, fertilizers, and animal feed). For example, *Pyropia* spp. are consumed worldwide. *Chondrus*, *Eucheuma*, and *Kappaphycus* serve as raw materials for carrageenan production. *Gelidium* and *Gracilaria* are agarophytes used in the agar industry (Reddy et al., 2010). In addition, red algae are important for eukaryotic evolution (Yoon et al., 2010). Over the last 20 years, research on the physiological ecology, evolution, and commercial importance of red algae has surged.

To date, the *Hsp20* gene family has been investigated in many plant species; for instance,19 *Hsp20* genes have been identified in *Arabidopsis* (Scharf et al., 2001), 39 in rice (Ouyang et al., 2009), 48 in potato (Zhao et al., 2018), 35 in pepper (Guo et al., 2015), 42 in tomato (Yu et al., 2016), and 51 in soybean (Lopes-Caitar et al., 2013). However, only a few studies have investigated the *Hsp20* gene families in algae. Currently, only six *Hsp20* genes have been identified in the green alga *Chlamydomonas reinhardtii* (Waters and Rioflorido, 2007), two in the unicellular red alga *Cyanidioschyzon merolae* (Kobayashi et al., 2014), and five in the multicellular red alga *Py. yezoensis* (Uji et al., 2019). Overall, systematic and comprehensive studies on the *Hsp20* gene family in algae are lacking. In recent years, with the development of high-throughput sequencing technology, high-quality and complete reference genomes of eight red algae have been assembled, annotated, and made available through public databases (Matsuzaki et al., 2004; Bhattacharya et al., 2013; Collen et al., 2013; Schoenknecht et al., 2013; Brawley et al., 2017; Lee et al., 2018; Cao et al., 2020; Wang et al., 2020). In addition, some red algal protein data without reference genomes are available on EukProt (Richter et al., 2020). These studies provide convenient and useful resources for further understanding the *Hsp20* gene families in red algae.

In the present study, we performed a comprehensive and systematic analysis of *Hsp20* gene families in red algae. We identified the members of the red algal *Hsp20* gene families using bioinformatic methods and analyzed the gene structure, conserved motifs, phylogenetic relationships, evolutionary origin, chromosome location, gene duplication, *cis-*regulatory elements, and expression profiles. These results provide valuable information for further functional characterization of red algal *Hsp20* genes and elucidation of the evolutionary history of red algal *Hsp20* gene families.

## Materials and Methods

### Genome-Wide Identification and Characterization of Heat Shock Protein 20 Gene Families in Red Algae

To identify *Hsp20* genes throughout the red algae, 17 kinds of red algae were chosen depending on the availability of genome or protein data (Supplementary Table 1). High-quality *Py. yezoensis* and *Pp. haitanensis* reference genome assemblies and protein sequences were obtained from our laboratory (Cao et al., 2020; Wang et al., 2020). *Chondrus crispus*, *Cy. merolae*, *Gracilariopsis chorda*, *Galdieria sulphuraria*, *Porphyridium purpureum*, and *Ph. umbilicalis* reference genome assemblies and protein sequences were downloaded from the National Center for Biotechnology Information (NCBI^2^). Other red algal protein sequences were downloaded from the EukProt database^3^. The hidden Markov model (HMM) profile of the Hsp20 domain (PF00011) was downloaded from Pfam^4^ to identify *Hsp20* genes in red algae. HMMER software version 3.0 (Finn et al., 2011) was used to search for Hsp20s from the red algal protein sequences with an *E*-value cutoff value of 0.001. According to the HMMER results, all candidate Hsp20s that may contain the Hsp20 domain were submitted to Pfam and CDD^5^ to confirm the Hsp20 domain. The red algal *Hsp20* genes were named according to their molecular weights. The molecular weight, instability index, and theoretical isoelectric points (pI) of the red algal Hsp20s were calculated using the ProtParam tool (Gasteiger et al., 2003). Subcellular protein localization was predicted using WoLF PSORT, CELLO, Yloc, BUSCA, MULocDeep, and SeqNLS (Yu et al., 2006; Horton et al., 2007; Briesemeister et al., 2010; Lin and Hu, 2013; Savojardo et al., 2018; Jiang et al., 2021).

### Phylogenetic Analysis and Classification of Red Algal Heat Shock Protein 20 Gene Families

First, we constructed a maximum likelihood (ML) phylogenetic tree to classify *Hsp20* genes in red algae. All highly conserved ACD sequences of the predicted red algal Hsp20s were aligned using the MEGA X-ClustalW program with default parameters. An ML tree was inferred using IQ-TREE 2.1.3 (Minh et al., 2020), and the best-fit substitution model was automatically selected using ModelFinder (Kalyaanamoorthy et al., 2017) implemented in IQ-TREE. Branch support was calculated using ultrafast bootstrap approximation with 1,000 replicates (Hoang et al., 2018). Finally, the phylogenetic trees were edited using EvolView (Subramanian et al., 2019).

Second, highly conserved ACD sequences were used to construct an ML phylogenetic tree to study the origin of red algal *Hsp20* genes. This conserved domain has proven to be useful in evolutionary studies of *Hsp20* genes (Caspers et al., 1995; de Jong et al., 1998; Huang et al., 2008; Kobayashi et al., 2014). The phylogenetic tree contained not only ACD sequences of the previously predicted red algal Hsp20s but also the other 154 ACD sequences of Hsp20s. Hsp20 protein sequences of *Cyanophora paradoxa* were downloaded from the *Cyanophora paradoxa* Genome Project (Price et al., 2019). Other Hsp20 protein sequences are shown in Supplementary Table 2. Multiple sequence alignment was performed using the MEGA X-ClustalW program with the default parameters. An ML tree was inferred using IQ-TREE 2.1.3 (Minh et al., 2020), and the best-fit substitution model was automatically selected using ModelFinder (Kalyaanamoorthy et al., 2017) implemented in IQ-TREE. Branch support was calculated using ultrafast bootstrap approximation with 1,000 replicates (Hoang et al., 2018). The ML tree was compiled and visualized using FigTree 1.4.4.^6^

### Gene Structure, Conserved Motifs, and Conserved Domain Analysis of Red Algal Heat Shock Protein 20 Gene Families

Exon-intron structures of the red algal *Hsp20* genes were identified using the TBtools software (Chen et al., 2020). Furthermore, conserved motifs of red algal Hsp20s were identified using the MEME program (version 5.4.1)^7^ with the following parameters: number of motifs 6 and optimum motif widths from 6 to 50 amino acid residues (Bailey et al., 2015). The conserved ACD of red algal Hsp20 protein sequences was aligned using the MEGA X-ClustalW program with default parameters. GeneDoc software^8^ was used for homology shading and scoring of the aligned sequences. Sequence logos were generated using WebLogo (Crooks et al., 2004).

### Chromosome Location and Collinearity Analysis

Chromosomal locations of *Hsp20* genes were visualized using TBtools with eight red algae genomic sequences and annotation files. In addition, using the One-Step MCScanX program of TBtools, we analyzed tandem duplication events and segmental duplication events of the red algal *Hsp20* gene family (Wang et al., 2012; Chen et al., 2020). Similarly, the One-Step MCScanX program of TBtools was used to analyze the collinearity relationship of *Hsp20* genes between different red algae. Furthermore, to determine the selection pressure, the rates of non-synonymous (Ka) and synonymous (Ks) substitutions were calculated using TBtools.

### Analysis of *Cis-*Acting Regulatory Elements in Red Algal Heat Shock Protein 20 Genes Promoters

To identify the *cis-*acting regulatory elements in the promoter sequences of red algal *Hsp20* genes, the upstream 1.5 kb promoter sequences of red algal *Hsp20* genes were submitted to PlantCARE^9^ (Lescot et al., 2002). The detected *cis-*acting regulatory elements were classified into different response types based on their annotated functions, and the number of detected *cis-*acting regulatory elements was displayed using a heatmap. In addition, we followed manual inspection and the motif-based sequence analysis tool MEME to search for heat-shock elements (HSEs).

### Expression Analysis of *Pyropia yezoensis* Heat Shock Protein 20 Genes

To investigate the expression pattern of *PyyHsp20* (*Py. yezoensis Hsp20*) genes, Illumina RNA-seq data were collected from previous studies conducted in our laboratory (Tang et al., 2019; Wang et al., 2020). The RNA-seq data were deposited in NCBI under BioProject PRJNA589917 and PRJNA560692. These data included different developmental stages and abiotic and biotic stresses. RNA-seq data analysis, including experimental design, quality control, read alignment, and quantification of gene and transcript levels, was performed as previously described (Tang et al., 2019; Wang et al., 2020). Furthermore, gene expression levels were quantified by fragments per kilobase of transcript per million fragments mapped (FPKM) values, and expression heat maps were created using TBtools software based on log2 transformed FPKM values. In addition, RNA-seq data including different parts of the blade were collected from previous studies conducted in our laboratory, and the FPKM values are given in Supplementary Table 3. RNA-seq data and gene expression analyses were performed as described previously.

## Results

### Identification of Heat Shock Protein 20 Genes in Red Algae

In this study, we analyzed 17 representative species of red algae with available genomic or protein information. A total of 97 putative *Hsp20* genes in red algae were identified and named based on their molecular weights. Two members were identified in *Cy. merolae*, three in *Ga. sulphuraria*, two in *Cyanidium caldarium*, five in *Ch. crispus*, six in *Gr. chorda*, nine in *Agarophyton chilense*, seven in *Madagascaria erythrocladioides*, eight in *Compsopogon caeruleus*, three in *Erythrolobus australicus*, six in *Porphyridium aerugineum*, eight in *Pr. purpureum*, seven in *Py. yezoensis*, eight in *Pp. haitanensis*, nine in *Ph. umbilicalis*, three in *Rhodella violacea*, six in *Rhodosorus marinus*, and five in *Stylonematophyceae* sp. Among the 97 putative Hsp20 genes, eight candidate sequences (*AcHsp20-14.9*, *AcHsp20-13.0*, *CocHsp20-7.6*, *PyyHsp20-20.3*, *PphHsp20-15.6*, *PphHsp20-20.8*, *PhuHsp20-20.5*, and *RomHsp20-13.4*) contained incomplete Hsp20 domains according to Pfam and CDD. Detailed information on the red algal *Hsp20* genes is shown in Supplementary Table 4, including names, chromosome locations, intron numbers, protein lengths, molecular weights, pI values, and instability index. The corresponding predicted molecular weights and length of encoded proteins varied from 6.61 to 49.27 kDa and 60 to 434 amino acids. The pI of Hsp20s ranged from 3.97 to 10.88. The instability index of Hsp20s ranged from 21.76 to 73.59. The protein sequences of the red algal *Hsp20* genes are provided in Supplementary Data Sheet 1. Protein subcellular localization is closely related to protein function, and their prediction is helpful in understanding protein function. In this study, we used six protein subcellular localization prediction tools to predict the subcellular location of red algal proteins. The prediction of the subcellular localization of red algal Hsp20s is shown in Supplementary Table 5. However, the results were not entirely consistent. Finally, we accepted the majority consensus.

### Phylogenetic Analysis of Red Algal Heat Shock Protein 20 Gene Families

**FIGURE 1:**
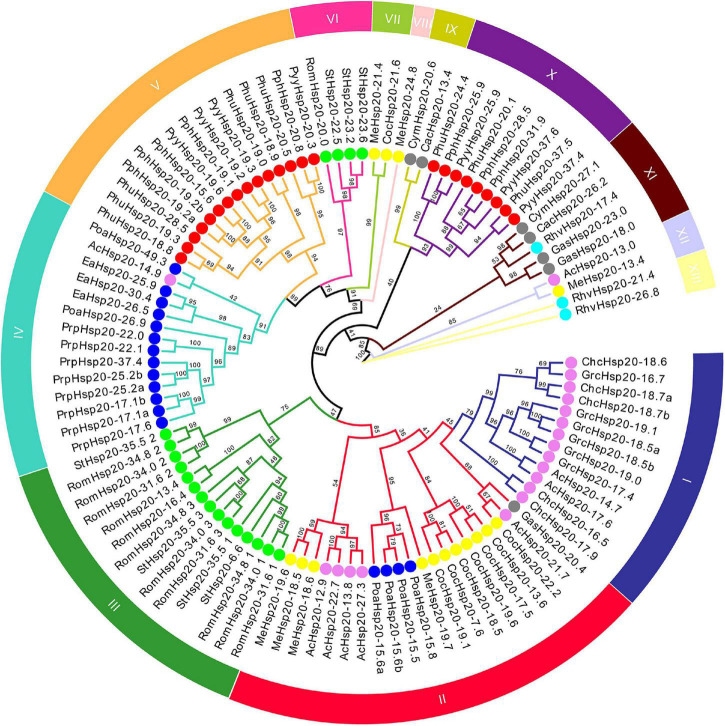
Phylogenetic tree of conserved ACD sequences of red algal Hsp20 proteins. The maximum likelihood (ML) phylogenetic tree of conserved ACD sequences of red algal Hsp20 proteins was constructed using IQ-TREE 2.1.3. The different subfamilies, numbered from I to XIII, were marked using different colors. Red, gray, violet, yellow, blue, cyan, and lime circles represent Bangiophyceae, Cyanidiophyceae, Florideophyceae, Compsopogonophyceae, Porphyridiophyceae, Rhodellophyceae, and Stylonematophyceae, respectively.

An unrooted ML phylogenetic tree was constructed based on the alignment of the conserved ACD sequences of the red algal Hsp20 protein sequences (Figure 1). Based on the phylogenetic tree of the conserved ACD sequences, all identified 97 *Hsp20* genes were classified into 13 distinct subfamilies. There were two types of subfamilies in the red algal Hsp20 gene families. One type was a broadly distributed subfamily, including the II, IV, XI, and XII subfamilies, which included members from different classes. For instance, in the II subfamily, eleven members were from the class Compsopogonophyceae, five members were from the class Florideophyceae, four members were from the class Porphyridiophyceae, and one member were from the class Cyanidiophyceae. The other type was a class-specific subfamily, such as the I, III, V, VI, VII, VIII, IX, X, and XIII subfamilies, which included members from the same classes. For instance, in subfamily I, all 13 members came from the same class, Florideophyceae. It is worth noting that only 4 of 97 red algal Hsp20s (RomHsp20-31.6, RomHsp20-34.8, RomHsp20-34.0, and StHsp20-35.5) belonging to the class Stylonematophyceae possessed three ACDs, and the other red algal Hsp20s possessed only one ACD. Most strikingly, in these 12 ACDs, ACDs from different Hsp20s clustered together rather than those from the same Hsp20. In addition, classification did not correlate with predicted subcellular localization.

**FIGURE 2:**
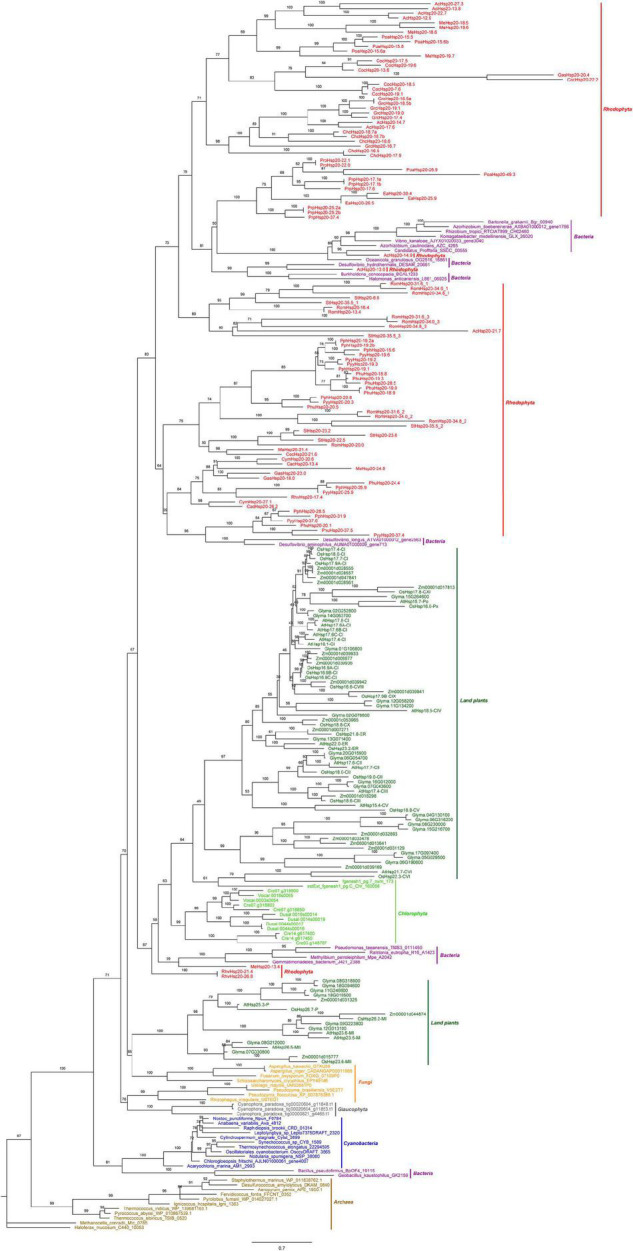
The maximum likelihood (ML) phylogenetic tree based on the conserved ACD sequences of Hsp20s. The tree was rooted by the ACD sequences of *Haloferax mucosum* Hsp20 protein. We applied different colors for species in different phyla, such as saddle brown for the archaea, purple for the bacteria, blue for the cyanobacteria, orange for the fungi, green for the land plants, gray for the Glaucophyta, lime for the Chlorophyta, and red for the Rhodophyta. Detailed information for the proteins included in the analysis can be found in the Supplementary Material.

A phylogenetic tree based on the conserved ACD sequences of 251 Hsp20s from various species of archaea, cyanobacteria, bacteria, fungi, algae, and plants was constructed using the ML method (Figure 2). The goal of our analysis was to elucidate the origin of red algal *Hsp20* genes. Phylogenetic analysis showed that archaea Hsp20s clustered at the base of the tree, followed by bacteria, cyanobacteria, glaucophyta, and fungi clades. Plant Hsp20s clustered into two major clades, and clustered according to the cellular localization. In green algae, apart from two Hsp20s, all green algal Hsp20s clustered together in one clade. In red algae, Hsp20s were always grouped together. It is worth noting that some red algal Hsp20s clustered with bacterial Hsp20s. This indicated that these red algal Hsp20s may originate from bacteria *via* horizontal gene transfer (HGT). In addition, none of the red algal Hsp20s belonged to land plant organelle-localized Hsp20s.

### Gene Structure, Conserved Motifs, and Conserved Domain of Red Algal Heat Shock Protein 20s

**FIGURE 3:**
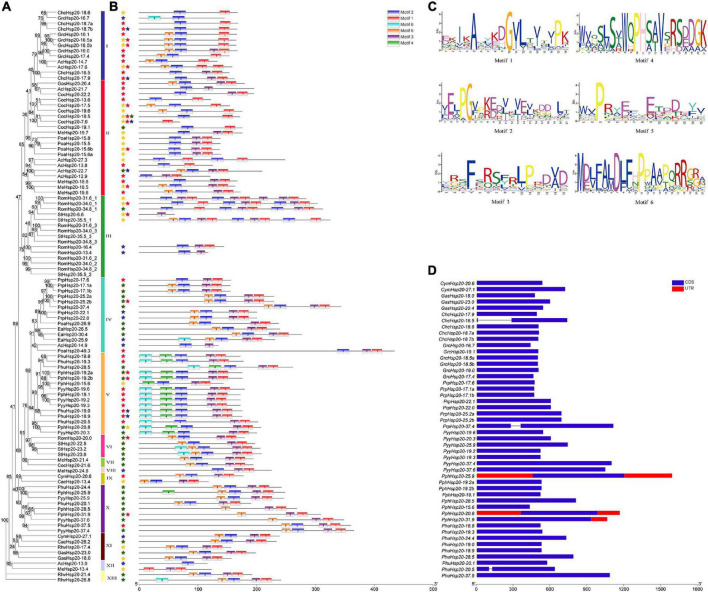
Gene structure and motif compositions of *Hsp20* genes in red algae. **(A)** Maximum likelihood (ML) phylogenetic tree and subcellular localization prediction. Red, yellow, blue, and green stars represent nucleus, cytoplasm, mitochondrion, and plastid, respectively. **(B)** Motif analysis was performed using the MEME software. Different colored boxes represent different motifs in the corresponding positions of each red algal Hsp20 protein. The scale at the bottom represents the length of the protein and motif. **(C)** Motif logos of the conserved motifs. **(D)** Gene structure was analyzed using the TBtools software. Boxes filled with blue represent exons, and solid black lines represent introns. The untranslated regions are indicated using red boxes. The scale at the bottom is in bp.

The gene structure plays a crucial role in the evolution of multiple gene families. Our results showed that most of the red algal *Hsp20* genes contained no introns and only three contained one intron (Figure 3D). In some studies, genes with few or no introns were considered to be rapidly activated in response to various stresses (Jeffares et al., 2008).

The MEME online tool was employed to identify conserved motifs in red algal Hsp20s, and six types of consensus motifs were identified (Figure 3B). The length of these conserved motifs varied from 15 to 21 amino acids (Figure 3C). The locations of the six motifs matched the conserved regions, as revealed through multiple sequence alignment analysis. As shown in Figure 3B, motifs 1, 2, and 3 were found in most members of the red algal *Hsp20* family. Motifs 4 and 6 were mainly found in the Hsp20s of *Py. yezoensis*, *Pp. haitanensis*, and *Ph. umbilicalis* belonging to the same class, Bangiophyceae. The type, order, and number of motifs were similar in proteins within the same subfamily but differed from those of other subfamilies, indicating that these genes may be highly conserved (Figure 3A). However, the functions of these highly conserved amino acid motifs remain elusive.

**FIGURE 4:**
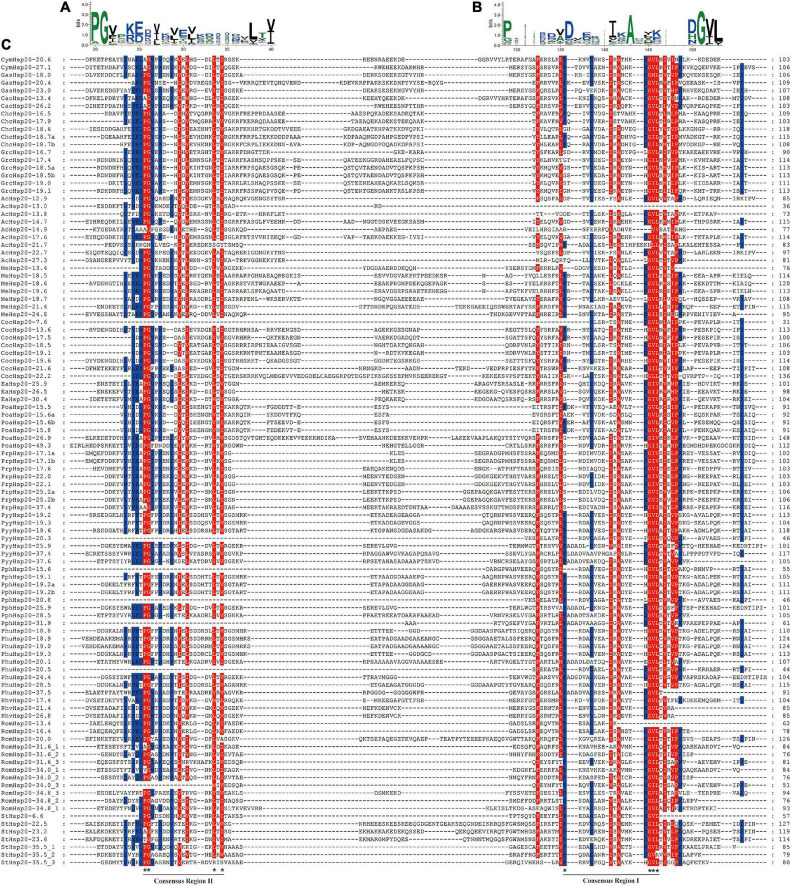
Multiple sequence alignment of α-crystalline domains of Hsp20s in red algae. **(A)** Sequence logo of the consensus II region. The height of individual symbols within a stack indicates the relative frequency of an amino acid at that position. **(B)** Sequence logo of the consensus I region. The height of individual symbols within a stack indicates the relative frequency of an amino acid at that position. **(C)** Multiple sequence alignment of the red algal Hsp20s containing consensus I and II regions. Conserved amino acid residues are indicated using color shading. Consensus I and II regions are underlined at the bottom and the typical amino acid residues within these regions are indicated by asterisks.

Multiple sequence alignments of the conserved ACD domains among the red algal Hsp20s are shown in Figure 4. In previous studies, the ACD domain was divided into consensus I and II domains separated by a hydrophilic domain of variable length in plants. These two conserved regions are separated by a hydrophilic domain of variable length and characterized by residues Pro-X (14)-Gly-Val-Leu and Pro-X (14)-X-Val/Leu/Ile-Val/Leu/Ile, respectively (Waters et al., 1996). These conserved regions are known to play important roles in the chaperone function of Hsp20s. To date, the structure of the ACD region in red algae remains relatively unknown. In our study, the ACD domain of red algal Hsp20s could also be divided into two parts (consensus I and consensus II) separated by a hydrophilic domain of variable length. Moreover, most motifs 1 and 3 were located in consensus region I, and most motifs 2 were located in consensus region II.

### Chromosomal Location, Gene Replication, and Collinearity Analysis of Red Algal Heat Shock Protein 20 Genes

**FIGURE 5:**
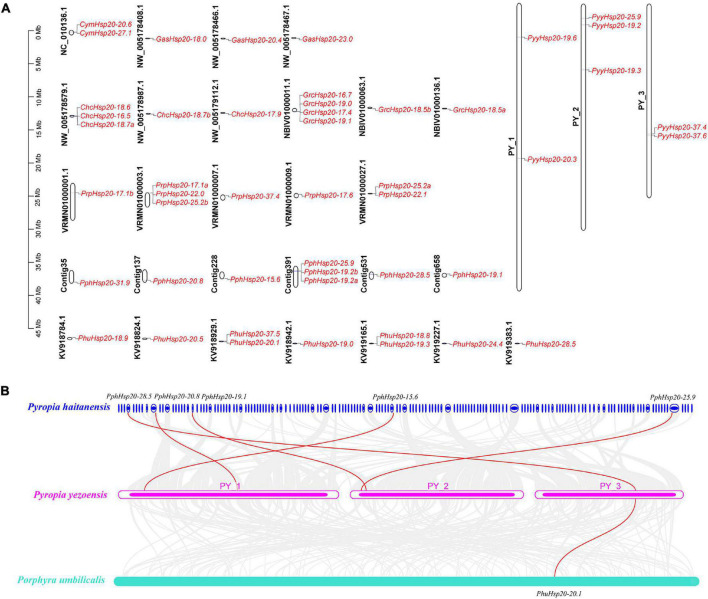
Chromosome location and synteny analysis of red algal *Hsp20* genes. **(A)** Chromosome location of *Hsp20* genes in eight red algae. **(B)** Synteny analysis of the *Hsp20* genes between three red algae. Gray lines in the background indicate collinear blocks within the three red algal genomes. The red lines highlight syntenic *Hsp20* gene pairs.

To better understand the chromosomal locations of red algal *Hsp20* genes, their positions on each chromosome were marked. As shown in Figure 5A, 48 *Hsp20* genes were mapped to 31 chromosomes. The number of *Hsp20* genes on each chromosome was between one and four. Except for *Cy. merolae* and *Ga. sulphuraria*, *Hsp20* genes were unevenly distributed on 27 chromosomes. In *Cy. merolae*, both *Hsp20* genes were located on one chromosome. In *Ga. sulphuraria*, the three *Hsp20* genes were evenly distributed on the three chromosomes. Interestingly, the chromosomal locations of *Ch. crispus* and *Gr. chordas* exhibited similar patterns. It might be related to their close relationship.

Subsequently, using the One-Step MCScanX program of TBtools, we analyzed tandem duplication and segmental duplication events. The results are shown in Supplementary Table 6. We identified five gene pairs with tandem duplication events and two gene pairs with segmental duplication events. Notably, these events occurred mainly in *Pr. purpureum*. These results suggest that these events are the main driving forces for the diversity of *Hsp20* genes in *Pr. purpureum*.

Furthermore, we explored the collinearity relationships among the eight red algae. As shown in Figure 5B (Supplementary Table 7), five *Py. yezoensis Hsp20* genes had collinearity relationships with *Pp. haitanensis Hsp20* genes and one *Py. yezoensis Hsp20* gene had collinearity relationships with *Ph. umbilicalis Hsp20* genes. The other five red algae species showed no collinearity. These results indicate that the *Hsp20* genes in *Py. yezoensis*, *Pp. haitanensis*, and *Ph. umbilicalis* are highly evolutionarily conserved.

TBtools was used to calculate the non-synonymous (Ka)/synonymous (Ks) ratios for each gene pair (Supplementary Tables 6, 7). When the Ka/Ks ratio is equal to 1, it shows a neutral selection; when it is >1, it denotes positive selection; when it is <1, it is used for purifying selection. The Ka/Ks ratios of tandem duplication, segmental duplication, and collinearity gene pairs were <1, indicating that the red algal *Hsp20* gene families might have experienced strong purifying selection pressures during evolution.

### *Cis-*Regulatory Element Analysis of the Red Algal Heat Shock Protein 20 Gene Families

**FIGURE 6:**
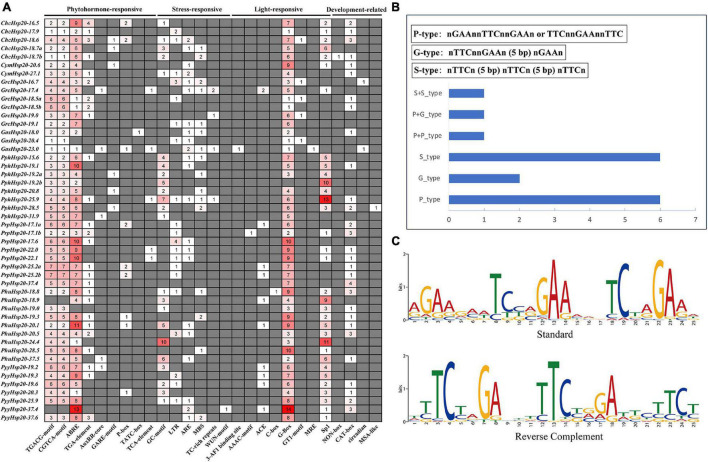
Predicted *cis-*elements in red algal Hsp20 promoters. **(A)** Analysis of 1.5-kb upstream *cis-*acting elements found in red algal *Hsp20* genes. The different colors and numbers of the grid indicated the numbers of different promoter elements. **(B)** Distribution of HSEs in red algal *Hsp20* promoters. **(C)** Motif logos of the putative heat shock elements (HSEs) in red algae *Hsp20* promoters. The motifs were obtained using MEME analysis based on promoter sequences of red algal *Hsp20* genes.

*Cis-*regulatory elements are important molecular switches involved in the transcriptional regulation of gene expression and control various biological processes, including stress responses, hormone responses, and developmental processes (Yamaguchi-Shinozaki and Shinozaki, 2005). To further explore the regulatory mechanisms of *Hsp20* genes in red algal growth, development, and stress response, the *cis-*elements in the promoter region (1.5 kb upstream sequences from the translation start sites) of the 48 red algal *Hsp20* genes were further analyzed. Four categories of *cis-*elements, phytohormone-, stress-, light-responsive, and development-related, were identified (Figure 6A). Among the four *cis-*element categories, the phytohormone-responsive category accounted for the highest proportion, including auxin, gibberellin, MeJA, abscisic acid, and salicylic acid-responsive elements. Among these elements, MeJA-responsive and abscisic acid-responsive *cis-*elements accounted for the largest proportion of the phytohormone-responsive category. In the stress-responsive category, stress response-related *cis-*elements, such as the GC-motif (anoxic specific inducibility), LTR (low temperature-responsive), ARE (anaerobic induction), MBS (drought-inducibility), TC-rich repeats (defense and stress responsiveness), and WUN motifs (wound-responsiveness) were detected. However, we did not find HSEs in the promoters of the red algal *Hsp20* genes using the PlantCARE tool. In general, the expression of *Hsp* genes was regulated at the transcriptional level by binding of heat shock transcription factors (HSFs) to HSEs. Eukaryotic HSEs are categorized into three types: perfect (P), gap (G), and step (S). P-type HSEs have three inverted repeats in a contiguous array (nGAAnnTTCnnGAAn or TTCnnGAAnnTTC). G-type HSEs have two consecutive inverted sequences, with the third sequence separated by 5 bp [nTTCnnGAAn (5 bp) nGAAn]. S-type HSEs have 5 bp gaps separating all three modules [nTTCn (5 bp) nTTCn (5 bp) nTTCn] (Mittal et al., 2011). We subsequently used manual inspection and the motif-based sequence analysis tool MEME to search for HSEs. In total, six, two, and six red algal *Hsp20* promoters contained only P-, G-, and S-type HSEs, respectively, and one red algal *Hsp20* promoter showed both P- and G-type HSEs. One red algal *Hsp20* promoter showed two P-type HSEs, and one red algal *Hsp20* promoter showed two S-type HSEs (Figure 6B and Supplementary Data Sheet 2). In addition, one motif showed consensus sequences similar to the known perfect HSE consensus sequence (Figure 6C). The main components of the light-responsive elements were G-box and Sp1. In the last category, plant development-related elements, including meristem-specific activation (NON-box), meristem expression (CAT-box), circadian, and cell cycle regulation (MSA-like) were identified. These results indicate that *Hsp20* genes play crucial roles in the growth, development, and stress response of red algae. In addition, there are some differences in *cis-*acting elements between different red algae species. For example, Sp1 was found in *Pp. haitanensis* but not in *Ga. sulphuraria*. These results suggest that *Hsp20* genes not only play common roles in different red algae species but also play specific roles in certain red algae species.

### Expression Patterns of *Py. yezoensis Hsp20* Genes Under Biotic and Abiotic Stresses

**FIGURE 7:**
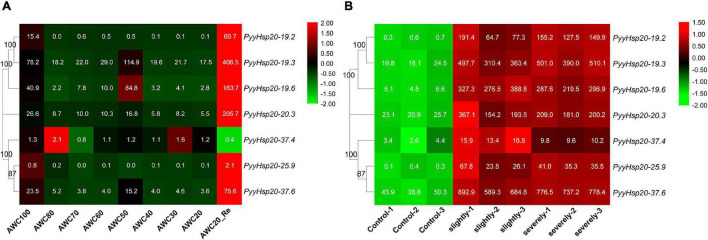
Heat map of the expression profiles of *PyyHsp20* genes under various biotic and abiotic stress conditions. **(A)** Heat map of the expression profiles of *PyyHsp20* genes under the dehydrated and rehydrated stresses. AWC100, AWC80, AWC70, AWC60, AWC50, AWC40, AWC30, and AWC20 represent water loss at 0, 20, 30, 40, 50, 60, 70, and 80%, respectively. AWC20_Re represents rehydration 30 min after 80% water loss. Differential expression is shown in different colors. The numbers represent relative expression quantity. Each experimental condition contained four biological repetitions. **(B)** Heat map of the expression profiles of *PyyHsp20* genes in *Py. yezoensis* during infection. Control represents a healthy blade of *Py. yezoensis*. Slightly represents a slightly infected stage. Severely represents severely infected stage. Differential expression is shown in different colors. The number represents relative expression quantity.

To explore the functions of red algal *Hsp20* genes in response to biotic and abiotic stress, we performed a comprehensive expression analysis using the available RNA-seq data of *Py. yezoensis* from our laboratory to investigate the expression patterns of *PyyHsp20* genes in response to dehydration/rehydration and red rot disease. Different expression patterns of *PyyHsp20* genes were observed in response to dehydration/rehydration and red rot disease (Figure 7). As shown in Figure 7A, under dehydrated and rehydrated stresses, except for *PyyHsp20-37.4* and *PyyHsp20-25.9*, the expression of all the other *PyyHsp20* genes was significantly downregulated under dehydrated conditions and significantly upregulated under rehydrated conditions. Moreover, *PyyHsp20-19.3*, *PyyHsp20-19.6*, *PyyHsp20-20.3*, and *PyyHsp20-37.6* were up-regulated under 50% water loss conditions. As shown in Figure 7B, the expression of all *PyyHsp20* genes was significantly upregulated when *Py. yezoensis* was infected with the oomycete pathogen *Pythium porphyrae*. Among these genes, *PyyHsp20-37.6* showed the highest expression level. These results indicated that most *PyyHsp20* genes responded to biotic and abiotic stresses, and the response mechanisms of different *PyyHsp20* genes to biotic and abiotic stresses were different. In conclusion, these results suggest that *PyyHsp20* plays a role in mediating the response of *Py. yezoensis* under environmental stress conditions.

### Expression Profiles of *Py. yezoensis Hsp20* Genes in Various Parts and Developmental Stages of *Pyropia yezoensis*

**FIGURE 8:**
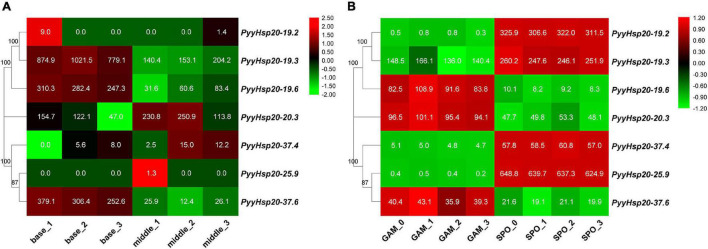
Heat map of the expression profiles of *PyyHsp20* genes in various parts and developmental stages of *Py. yezoensis*. **(A)** Heat map of the expression profiles of *PyyHsp20* genes in different parts of the blade in *Py. yezoensis*. Differential expression is shown in different colors. The number represents relative expression quantity. **(B)** Heat map of the expression profiles of *PyyHsp20* genes in different developmental stages of *Py. yezoensis*. Differential expression is shown in different colors. The number represents relative expression quantity.

To investigate the function of *PyyHsp20* in *Py. yezoensis* growth and development, we analyzed the expression profiles of *PyyHsp20* genes in different parts and developmental stages of *Py. yezoensis*. Significant differences were observed in the expression profiles of *PyyHsp20* genes at different developmental stages and different parts (Figure 8). First, RNA-seq data were used to explore the expression levels of *PyyHsp20* genes in two different parts of the blade: the base and middle parts. Cell morphology and size differed significantly between the two parts of the blade. However, this finding has not yet been reported. As illustrated in Figure 8A, most *PyyHsp20* genes were expressed in two parts, except for *PyyHsp20-19.2* and *PyyHsp20-25.9*, which were barely expressed in any part. The expression levels of three *PyyHsp20* genes (*PyyHsp20-19.3*, *PyyHsp20-19.6*, and *PyyHsp20-37.6*) in the base part were relatively higher than those in the middle part. Second, RNA-seq data were used to explore the expression levels of *PyyHsp20* at two different developmental stages, including leafy gametophytes and filamentous sporophytes (Figure 8B). All *PyyHsp20* genes were expressed in the sporophyte, and most *PyyHsp20* genes were expressed in the gametophyte, except for *PyyHsp20-19.2* and *PyyHsp20-25.9*, which were present at almost undetectable levels. The expression levels of *PyyHsp20-19.2*, *PyyHsp20-19.3*, *PyyHsp20-37.4*, and *PyyHsp20-25.9* were higher in sporophytes than in gametophytes. Conversely, the expression levels of *PyyHsp20-19.6*, *PyyHsp20-20.3*, and *PyyHsp20-37.6* in sporophytes were lower than those in gametophytes. These results indicate that *PyyHsp20* genes showed spatial and temporal expression patterns during different developmental stages and in different parts of *Py. yezoensis*. In conclusion, these results suggested that *PyyHsp20* plays a crucial role in the growth and development of *Py. yezoensis*.

## Discussion

### Identification and Characterization of the Red Algal Heat Shock Protein 20 Gene Families

Although *Hsp20* gene families have been investigated in many plant species, only a few *Hsp20* genes have been identified in red algae. Moreover, a comprehensive analysis of the *Hsp20* gene family in red algae is lacking. Therefore, we analyzed 17 representative species covering all seven classes of Rhodophyta with available genomic or transcriptomic information. Detailed information on the red algal Hsp20 genes is shown in Supplementary Table 4, including names, chromosome locations, intron numbers, protein lengths, molecular weights, pI values, and instability index. However, some information, such as chromosome locations and intron numbers, may be incomplete owing to the lack of a relevant reference genome. To date, only eight high-quality and complete reference genomes of red algae have been assembled, annotated, and made available through public databases (Matsuzaki et al., 2004; Bhattacharya et al., 2013; Collen et al., 2013; Schoenknecht et al., 2013; Brawley et al., 2017; Lee et al., 2018; Cao et al., 2020; Wang et al., 2020). In addition, some red algal protein data without reference genomes are available through EukProt (Richter et al., 2020). Although some of the red algal protein sequences in EukProt are incomplete, such as StHsp20-6.6, their Hsp20 domains are complete. Therefore, we chose these red algal Hsp20s to study the classification and origin of the red algal *Hsp20* gene families. We believe that a unified classification for Rhodophyta would be beneficial for future studies, when genomic data are available for these taxa. In addition, these genomic data will soon be available owing to rapidly developing sequencing technologies and declining costs, which will be of great help in the study of the red algal *Hsp20* gene families.

Protein subcellular localization is closely related to protein function, and its prediction is helpful for understanding protein function. In general, proteins can only perform their functions at specific subcellular positions (Wang et al., 2014). Currently, numerous programs or websites are available for the prediction of protein subcellular localization, such as WoLF PSORT, CELLO, Yloc, BUSCA, MULocDeep, and SeqNLS (Yu et al., 2006; Horton et al., 2007; Briesemeister et al., 2010; Lin and Hu, 2013; Savojardo et al., 2018; Jiang et al., 2021). However, no software has been specifically designed to predict the subcellular localization of red algae proteins. An earlier study reported that a subcellular localization prediction tool called PredAlgo is dedicated to green algae (Tardif et al., 2012). Tardif et al. (2012) also pointed out that currently available predictors are unreliable when used to predict the localization of algal proteins. However, the PredAlgo website is currently unavailable. In addition, the use of PredAlgo is inappropriate for red algal proteins (Mori et al., 2016). To accurately predict the subcellular location of red algal proteins, we used six protein subcellular localization prediction tools to predict the subcellular location of red algal proteins. The prediction of the subcellular localization of red algal Hsp20s is shown in Supplementary Table 5. Unfortunately, the results of the six tools are not entirely consistent. The reasons for this may be as follows: First, existing algorithms for protein subcellular localization prediction are not suitable for red algal proteins because of their unique cell structure, such as the presence of unstacked thylakoids and phycobilisomes in the chloroplast. Second, there is little experimental evidence of the subcellular localization of red algal proteins. Finally, we accepted the majority consensus. Therefore, it is necessary to develop a new algorithm with the specific aim of predicting red algae targeting. This will be helpful to study protein function in red algae. In addition, more experimental evidence about the subcellular localization of red algal proteins is required.

### Structure and Function of the Red Algal Heat Shock Protein 20 Genes

The gene structure plays a crucial role in the evolution of multiple gene families. The gene structure of Hsp20 proteins were investigated where genomic data were available. In addition, gene duplication, chromosome location, collinearity analysis, and *cis-*regulatory element analysis were explored in the Hsp20 proteins of eight red algae. Our results showed that most of the red algal *Hsp20* genes contained no introns, and only three contained one intron, suggesting relatively simple gene structures. This is consistent with previous results in higher plants (Zhao et al., 2018). In some studies, genes with few or no introns were considered to be rapidly activated in response to various stresses (Jeffares et al., 2008). The instability index provides an estimate of protein stability in a test tube, which can be predicted, as described by Gasteiger et al. (2005). A protein whose instability index is smaller than 40 is predicted to be stable, and a value above 40 predicts that the protein may be unstable. In our study, the instability index of most red algae Hsp20s was greater than 40, indicating that most of them were unstable proteins (Supplementary Table 4). Instability is also considered to be a common trait of stress-responsive proteins. *Hsp20* genes are considered as one of the rapidly expressed genes under various stress conditions. The absence of introns and the presence of instability may be in accordance with the need for rapid induction of *Hsp20* genes in response to various stresses and rapid disposal of proteins after the stress response.

The function of *Hsp20* genes has been systematically investigated in many higher plants (Scharf et al., 2001; Ouyang et al., 2009; Lopes-Caitar et al., 2013; Guo et al., 2015; Yu et al., 2016; Zhao et al., 2018). *Hsp20* genes not only play important roles in plant responses to various stresses but are also involved in plant developmental processes (Yu et al., 2016). However, the function of *Hsp20* in red algae is relatively unknown. *Py. yezoensis* is one of the most economically important marine red algae worldwide, and is recognized as an ideal model for studying the molecular mechanisms of stress resistance (Wang et al., 2020). In this study, *Py. yezoensis* was used as a representative of red algae to explore the expression profiles of *Hsp20* genes under abiotic (dehydration/rehydration) and biotic (red rot disease) stresses. The data demonstrated that numerous *PyyHsp20* genes were significantly induced to a larger extent under dehydration/rehydration stress and showed differential expression patterns, indicating that different *PyyHsp20* genes may play different roles in response to dehydration/rehydration stress. Previous studies have shown that *PyyHsp20* genes are induced by other abiotic stresses, such as heat, oxidative, and copper stress (Uji et al., 2019). Furthermore, the *Hsp20* gene in *Cy. merolae*, and *Pp. haitanensis* are also induced by heat stress (Kobayashi et al., 2014; Chang et al., 2021). Several *Hsp20* genes also participate in the interactions between plants and pathogens, such as viruses, bacteria, and fungi (Park and Seo, 2015). Tang et al. (2019) previously found that *PyyHsp20* genes showed increased expression during the slight and severe stages of the oomycete pathogen *Pythium porphyrae* infection. In the present study, we found that all *PyyHsp20* genes are induced by pathogen infection. These results were inconsistent with the report that biotic stress can induce the expression of some, but not all *Hsp20* genes (Li and Liu, 2019). In higher plants, spatiotemporal regulation of the *Hsp20* gene family has been observed in various tissues and developmental stages (Yu et al., 2016). In our study, spatiotemporal regulation of the *Hsp20* gene family was also observed in different parts and developmental stages of *Py. yezoensis*. *Py. yezoensis* exhibits a haploid-diploid heteromorphic life cycle with a haploid macroscopic blade-forming gametophyte and a diploid microscopic filamentous sporophyte. In addition, *Py. yezoensis* require fertilization and meiosis for the transition from gametophytes to sporophytes and from sporophytes to gametophytes, respectively. In our study, we found that some *PyyHsp20* genes were highly expressed in sporophytes, whereas some *PyyHsp20* genes were highly expressed in gametophytes. In the two different parts of the blade, *PyyHsp20* genes also displayed differential expression. These results indicate that *Py. yezoensis* seems to have established a sophisticated mechanism to tightly regulate the expression of *PyyHsp20* genes, where and when required.

*Cis-*regulatory elements are important molecular switches involved in the transcriptional regulation of gene expression and control various biological processes, including stress responses, hormone responses, and developmental processes (Yamaguchi-Shinozaki and Shinozaki, 2005). *Cis-*regulatory element predictions have been widely used to explore the functions of Hsp20s in several species (Wang et al., 2021). In our study, various stress-responsive, phytohormone-responsive, light-responsive, and plant development-related *cis-*elements were found in the promoter regions of red algal *Hsp20* genes (Figure 6). Among the four *cis-*element categories, the phytohormone-responsive category accounted for the highest proportion. Phytohormones are a large category of small endogenous, low-molecular-weight molecules that not only regulate plant growth and development at low concentrations, but can also act as signaling molecules that participate in plant responses to environmental stresses. Phytohormones such as auxin, ethylene, jasmonic acid, salicylic acid (SA), abscisic acid (ABA), cytokinin (CK), and gibberellins (GAs) can play important roles in plant development and stress responses (Saidi and Hajibarat, 2019). Emerging studies suggest that red algae contain phytohormones such as auxin, ethylene, ABA, CK, GAs, jasmonic acid, SA, and methyl jasmonate (MeJA) (Lu and Xu, 2015; Song et al., 2017). In higher plants, phytohormones have been shown to be linked to heat stress signaling and modulate the expression of Hsp under heat stress (Zhang and Wang, 2011; Nazar et al., 2017). Previous studies have reported that *Hsp20* genes can be induced by the exogenous application of MeJA, MeJA/SA, and the ethylene precursor 1-aminocylopropane-1-carboxylic acid (ACC) in the red algae *Ch. crispus*, *Gracilariopsis lemaneiformis*, and *Py. yezoensis* (Collen et al., 2006; Wang et al., 2017; Uji et al., 2019). In addition, *cis-*regulatory elements in the promoters are involved in the cross-talk of different stress signals in gene expression and constitute gene expression cascades during abiotic stress responses and control the molecular processes of stress responses and stress tolerance (Yamaguchi-Shinozaki and Shinozaki, 2005). Thus, we may assume a crosstalk between Hsp20s and phytohormones in the response of red algae to environmental stresses. Taken together, these genes may play important roles in regulating the growth, development, and stress responses of red algae. However, further analysis is needed to investigate how red algal *Hsp20* genes perform their functions.

### Evolution of the Red Algal Heat Shock Protein 20s

Hsp20s is a ubiquitous protein family found in archaea, bacteria, and eukaryotes. All organisms possess Hsp20s, indicating that these proteins evolved very early, prior to the divergence of the three domains of life (Archaea, Bacteria, and Eukarya) (Waters, 2013). A previous study reported that Hsp20s was the first in the last universal common ancestor (LUCA) (Rebeaud et al., 2021). Plant Hsp20s are particularly diverse and numerous in number. In evolutionary terms, they have exhibited lineage-specific expansion patterns (Waters and Vierling, 2020). According to their subcellular localization, plant Hsp20s are classified into several subfamilies (CI-CVI, MTI, MTII, ER, CP, and PX) (Waters and Vierling, 2020). Previous studies based on the limited number of algal Hsp20s sequences suggested that none of the algal Hsp20s sequences were found within the land plant lineage of cytosolic and organelle-localized proteins (Waters and Rioflorido, 2007). However, it is important to note that although algae do not possess close homologs of land plant CP Hsp20s, chloroplast-targeted Hsp20s are present in *Cl. reinhardtii* (Waters and Vierling, 2020). Further analysis indicated that algal Hsp20s are closely related only to Hsp20s from the same species (Waters and Rioflorido, 2007), which is different from the higher plant Hsp20s. Higher plant Hsp20s are more closely related to members of the same subfamily from divergent species than to other Hsp20s from the same species (Waters et al., 1996). In our study, red algal Hsp20s were classified as not based on predicted subcellular localizations. This might be because the lack of experimental data for the red algae organisms studied and the existing tools used to predict subcellular localization are inaccurate for red algae, or because the red algal *Hsp20* gene families might have distinct but currently unclear classification mechanisms. In addition, we identified two types of subfamilies in the red algal Hsp20 gene families. One type was a class-specific subfamily that included members from the same class. The other type is a broadly distributed subfamily that includes members from different classes (Figure 1). In the broadly distributed subfamily, red algal Hsp20 genes were always closely related to Hsp20 genes from the same species. This suggests that a significant expansion of red algal *Hsp20* genes occurred after the divergence of the red algae (Rhodophyta). Furthermore, we found that only four of 97 red algal Hsp20s belonging to the class Stylonematophyceae possessed three ACDs, and the other red algal Hsp20s possessed only one ACD. Moreover, among these 12 ACDs, ACDs were more closely related to ACDs from different Hsp20s than to ACDs from the same Hsp20s. This implies that *Hsp20* genes in the class Stylonematophyceae might have experienced a specific evolutionary process. In our study, red algal Hsp20s were distinct from those of land plants and green algae, indicating that Hsp20s probably evolved independently in red algae and plants (green algae) (Figure 2). Furthermore, some red algal Hsp20s clustered with bacterial Hsp20s, indicating that these red algal Hsp20s may originate from bacteria *via* HGT, while some red algal Hsp20s were not clustered with bacterial Hsp20s. Taken together, Hsp20 genes may have a polyphyletic origin and a complex evolutionary pattern. In addition, plant Hsp20s clustered according to cellular localization, which was consistent with the findings of a previous study (Huang et al., 2008; Waters and Vierling, 2020). Our study provides new insights into the evolution of Hsp20 gene families in red algae.

## Conclusion

In this study, 97 putative *Hsp20* genes were identified in red algae. A comprehensive analysis of red algal *Hsp20* genes using gene structures, conserved motifs, phylogenetic relationships, chromosome location, gene duplication, and *cis-*regulatory elements was performed. Finally, *Py. yezoensis* was selected as a representative of red algae to explore the expression profiles of *Hsp20* genes under different stresses, at different developmental stages, and in different parts of the blade using RNA-seq data. This study provides comprehensive information on *Hsp20* gene families in red algae and lays a foundation for their functional characterization. In addition, our study provides new insights into the evolution of Hsp20 gene families in red algae and can help understand the adaptability of red algae to diverse environments.

## Data Availability Statement

The original contributions presented in the study are included in the article/Supplementary Material, further inquiries can be directed to the corresponding author/s.

## Author Contributions

TG collected the public dataset, analyzed the data, and performed the experiments. TG and ZM drafted the manuscript. LT and XY contributed to bioinformatics analysis and gene expression profile analysis. ZM and YM conceived and designed the study. GD and YM reviewed the manuscript. All authors have read and approved the final manuscript.

## Conflict of Interest

The authors declare that the research was conducted in the absence of any commercial or financial relationships that could be construed as a potential conflict of interest.

## Publisher’s Note

All claims expressed in this article are solely those of the authors and do not necessarily represent those of their affiliated organizations, or those of the publisher, the editors and the reviewers. Any product that may be evaluated in this article, or claim that may be made by its manufacturer, is not guaranteed or endorsed by the publisher.

## Footnotes

1
http://www.algaebase.org/

2
http://www.ncbi.nlm.nih.gov/

3
https://doi.org/10.6084/m9.figshare.12417881.v2

4
http://pfam.sanger.ac.uk/

5
https://www.ncbi.nlm.nih.gov/Structure/bwrpsb/bwrpsb.cgi

6
http://tree.bio.ed.ac.uk/software/figtree

7
https://meme-suite.org/meme/tools/meme

8
https://github.com/karlnicholas/GeneDoc

9
http://bioinformatics.psb.ugent.be/webtools/plantcare/html/
