# Supplementary material for: Heat Shock Protein 20 Gene Superfamilies in Red Algae: Evolutionary and Functional Diversities
Source: Front Plant Sci. 2022 Mar 16;13:817852. doi: 10.3389/fpls.2022.817852 (PMC8966773; doi:10.3389/fpls.2022.817852)
Supplement: Supplementary file 8 [file Data_Sheet_1.PDF]

Supplementary Data Sheet 1. The protein sequences of red algal *Hsp20* genes

>ChcHsp20-18.6
MFSLLPFHYGMSAPFSSFDDDMWRPRNRLGMTESDRGHTQRIEPTFSIESDDDGAHFEIEL
PGVCKEDISIEAENNTLTVSAKRFKKHRTGKDDTVVEEEGKDGAEATKHPATKKDVQPKII
YVLQTRLGHGADVDAIEADVEGDGILYVTVPLKKGNGPRRIQIPI
>ChcHsp20-16.5
MEVAFHDRRLAPPFASSLGGLSARAPASAAPTCEVEPSYSQHGAKDAVFIEVELPGVARAD
LHVTVQGRRLVVVGKRFREPPRDAASEEAAESPQKADEKSADKQTAKTFKAVFRVPQIVD
VDGIETVAHKDGVLTLRLPHAKAAQPRKIEIQ
>ChcHsp20-18.7a
MSFLTPYNHHSLFPWGNPFSNDLRTMRRMLDMTESGARSTHTVEPSYRYEADGEAAHFEI
EIPGVSKDNLSVEVHDNKLTVRGKRFRRPLIEKKDDDPAAAKDPAAQNGDPAHPGEDPVP
SIVYLLEARLPQGANVDAIKADHVGDGILNMTIPMVADKGTRKIQIEF
>ChcHsp20-18.7b
MSILTPYNRHSFFPWGNSFGNDLRTMRRMLDMTESGPRSTHTVEPSYRYEADSAAAHFEI
EIPGVSKDNLSVEVHENKLTVRGKRFCRPLIEKKDNSAVAKDPAQNGDPDQADKDPAPSIV
YHLEARLPQRANVDAIKADHIGDGILNMTIPMVDDKGPRKVQIEF
>ChcHsp20-17.9
MMYQIAPFSTHLTFHDRRLPHPFASSLGGLSACMPASAARTSEVEPSFSQHSAEGALLVEIE
LPGVARSDLQVTMQGRRLVVVGKRFKERRQDAASEETAEAQEKADEKSTEKQAAKTFK
AVFHVPQIVDVERIETVAHKDGVLTLRLPHAKASQPRKIEIE
>CymHsp20-20.6
MSYYFFDPFEDLMDDWFFGFPRVSRRRQAALQQQQQQQYDDQKPAKANGVVARPQNV
MAMITPRVDFKETPEAYEINAELAGVPRDQVKVELHGDLLTIRGEKREENRAEEKDEGGR
VVYLRTERAFGAFERSLKLPKNVDRNSIKATHKDGVLNIVINKLKKDEDEKMNIEVTEA
>CymHsp20-27.1
MQYVLRSLQGNLKMLGQASAKLNFVVPFTGSSVVLRRPLMTPVGGRSPYAPALARSVAK
RSLSSARTSLMQANANSRERDNERTLVRGRRDWGWPFSIMRRDPFFMSPLMDLDRFWSD
FDALAQRSNAYLPALDITETNDAFVVSCELAGVPRENVKIALDGDILTVQGEKKWEHEEK
DAKMHRMERSYGSFSRSVRLPTDVVDAENIKAQHKDGVLRITIPKKVKQQENVKEIPIEV
S
>GasHsp20-20.4
MSSLLPFVDFLDPWDVFERASWSLDSDTERPEQTEGKNESNRKRGGRKGWVPRVEVTED
EQGNLNLDAEVPGVNSENLQLDVREGSLVISGVKRRQTEVTQNQVENDKDEVKRPPAKK
SKKDHPYVYSERQYGKFRRVVKLPKEVDVSKITASCKDGVLHVFIPVQEEASKISVPIEFS
>GasHsp20-23.0
MFVVQEILRDDCSRMVKVFNYRRPARLLSSSTRMRAGDPERRELSRPMRTLDSAFDELLA
FAQDPWAMFRSPWSMTPRNMAVDQWMPRVDLVEKEDGFYAYVELPGLSRENVKVEVRG
EVITISGEKKDEAKSESEKNGVVYHRMERSYGSFQRSLRIPPQVEKDKIKAVCKDGVLTVT
MPKRHVEKQDAKTIEIHAE
>GasHsp20-18.0
MSELVRPGRFFDSSFGDLFSWATDPFYRDIWSVTPRSIGEGQIWSPRVDLVEKDDCFLVKA
EVPGVPKENINVDLKGDILTVSGEKADERKSDEEREGTVYHRMERSYGKFERSIRLPKHID

RKGIKANCKDGMLTVTVPKKQVEKSESQKIEIANE
>GrcHsp20-16.7
MRVSAYDYFWPFCDDLSLLHSLFRVPPLSAQYGSRHVGFLYPSYSYKSKDGKAFIEIELPG
VEKDDIHVETRGHTLTVKAKRFDHGTTDKGKEKARATYSFKARLSDRVNMDEIRADYPG
LGVLRIFVPYKAKEAEKVRKVPVHS
>GrcHsp20-19.1
MALSLNVHDPFFPLSSFDRDMHSFLRLADRLGTVHSNRDRTNYQVPNYEYNRDKDNFHL
QVELPGVAKEHIEVEMNSGNLTITARRFRSSPAQPVSQEPDQSTENTGENDANKTRSLKPS
HVYKMQVKIGNNVDEEGIKAEYNHGVLELIIPQRKMTTRKISITDL
>GrcHsp20-18.5a
MALSRNVYDPLFPLSSFDRDMDSLLRLANRINVRPNADGTTYQVPNYEYQRDNDNFHLQ
VELPGVAKEHIDVKINTGNLTIIARRFRPSQAESGSQKPEQSTQVTAENDADVTQSPKPSHV
YKMQVEVGSTVDEDAIKADYNHGVLSMVIPLKKISARTIAITEH
>GrcHsp20-18.5b
MALSRNVYDPLFPLSSFDRDMDSLLRLANRINVRPNADGTTYQVPNYEYQRDNDNFHLQ
VELPGVAKEHIDVKINTGNLTIIARRFRPSQAESGSQKPEQSTQVTAENDADVTQSPKPSHV
YKMQVEVGSTVDEDAIKADYNHGVLSMVIPLKKISARTIAITEH
>GrcHsp20-19.0
MALSDGVFDPFFPLPSFDSDMRPLLRLADRVVNAYPITDRTTIQVPNYEYQRNNDKITLQV
ELPGVAKEHIDVEMNSGKLTITARRLRLSHAQPSSKESEQSTEENEAHEAQKAELSQMSRL
YKLQVRISTNVDDEGIKAEYNHGILNMILPLKKIDSARRIAITDQ
>GrcHsp20-17.4
MSFDREMRSQRRFADRSENVCLKTNRTIYKAPNYEFQRDNDNINLQVELPGVAKEHIDLE
MNSGKLTITARRFKASHSQPPSKESEQATEEKGGRDAHEAELPQPSCVYKLHVKTGTNVD
DEGIKAEYNHGILNIVLPMKKVNTARKIALTDI
>PrpHsp20-17.6
MALRVRTSVPMAFPDSRHMTWDSLIDSFFNIPVEDREQRVYKPTIAGPTSFFHEVDNKFVL
TIEAPGIPKENIKLEVKDNVLKISGEAEQAKENQDSENGKATPHFYRARSFQRSISLGNDIA
QDQITATAKDGVITIELPKRVREDPPVRTISVL
>PrpHsp20-17.1a
MALRARGLGPLASLRGSRGNTWDLIDKIMDAPFGSARNEWNRMFDPTLVGPPSEMQEFD
DKFVVNIEAPGIPKENIKLEVKDNVLKVSGKLESSEGRADGPMHSFSSRAFEQSMDLGNDI
MVDKIAASAKDGIVSIELPKREYVESSATRVIDIQ
>PrpHsp20-17.1b
MALRARGLGPLASLRGSRGNTWDLIDKIMDAPFGSARNEWNRMFDPTLVGPPSEMQEFD
DKFVVNIEAPGIPKENIKLEVKDNVLKVSGKLESSEGRADGPMHSFSSRAFEQSMDLGNDI
MVDKIAASAKDGIVSIELPKREYVESSATRVIDIQ
>PrpHsp20-22.1
MRVYHIRPSVVKRLATKSTKRNQERAAKSVAKRASSPMALRTRPAVSSHLAYPTGRANTF
ELIDRLLGAPFGTTRDDWNSLLEPVRASPPSLLSEFDDKYVMTIEAPGIPKEKIQLEVKDN
VLKVSGGMEENVTEDGENGDGKGEKVTHGYVARSFHRSLTLGNDVDIDKISASAKDGIV
TIELPKHGREEPATRLIEIK
>PrpHsp20-22.0
MRVYHIRPSVVKRLATKSTKRNKEKAAKSVAKRASSPMALRTRPAVSSHLAYPTGRANTF

ELIDRLLGAPFGTTRDDWNSLLEPVRASPPSLLSEFDDKYVMTIEAPGIPKEKIQLEVKDN
VLKVSGGMEENVTEDGENGDGKGEKVTHGYVARSFHRSLTLGNDVDIDKISASAKDGIV
TIELPKHGREEPATRLIEIK
>PrpHsp20-25.2a
MVKSDGMAFTFGVAPTIRVYGAKPSYMQTSPQNAPRKEGDGNSNGNVAIRTQPPGQSRA
MARPNRQMFAPLSPFAPLSPFDLMDAAERLMNTPFEQWDRAMGLAPAQQRAWSPTYKFE
EFDDKYVLYVEAPGIPKEKVVLEVKDSMLLISGNLEEKTTKPSDGGEPAGQKGAVFESYT
KRSFKRSLVLSEDILVDQISAAAKDGVISVVLPKRPKPEEPKPKRIEIASH
>PrpHsp20-25.2b
MVESDGMAFTFGVAPTIRVYGAKPSYMQTSPQNAPRKEGDGNGNGNVAIRTQPPAQSRA
MARPNRQMFAPLSPFAPLSPFDLMDAAERLMNTPFEQWDRAMGLAPAQQRAWSPTYKFE
EFDDKYVLYVEAPGIPKEKVVLEVKDSMLLISGNLEEKTTKPSDGGEPAGQKGAVFESYT
KRSFKRSLVLSEDILVDQISAAAKDGVISVVLPKRPKPEEPKPKRIEIASH
>PrpHsp20-37.4
MKKYSGKQSRSPTFQARDNEAASAVPAKTPLRPCAVWKARTAWPTGTLTAWRMAHDAL
ARPASSVRCKVGARYKNMRADMAFDGQLTLLEQTAVQSDTSVLIITNIIKMEKAANAMAF
MFGVVPGARVYGVRPSYSQASPQTNRVGGCASDGAVAGPPGRANARAARYMVPSMSPF
GVPINALDLLNVAERMMTTPFDQWDRAMGVSPAQRMWSPLYRYEEFDDKYVLYVEAAG
IPKEKVVLEVKDNVLLISANMEEKTAKRAEDTDNTAADAAAAESVSSKGTTMDSFRKRSF
ERRLTLSEDIVADNISAAAKDGIISVTLPKRAKPEAPKPKRIEIVNNTD
>PhuHsp20-18.8
MDFFALDLFQPPAPPQQRRGSAANPWGLWRPMMDPSLMNSAWQPHAAVTRSDDGKALN
VRVETPGFPRDHLNIELSDDRSLLTVSGSMHKETTTEEGGDGGDAASPGAFTSIQERQFSQ
SYRLPRDADVENIKADYVHGVLSVTVPTKGADELPQKRSIAIEDKTPAAKGSE
>PhuHsp20-19.3
MDVFALDLFHPPAPTQRRRDRAANPWGLWRPMVDPSYMNALSAWQPHSAVSRSDDGKA
LNVRVETPGFPRDHLNIELSDDRCLLTVSGSMHKETTTEEGGDGCDGGDAASPGACTSIEE
RQFSQSFRLPRDADVENIKADYEHGVLCVTVPTKGADALPQKRSIAIEDKTPAAKGTE
>PhuHsp20-24.4
MAFIPAAGAALGLRAPAAGAALGVVARRVAATPRLPRVSMATATPSDGAKGAADAGDAP
ESPAGASAPRHGGGGMVRRRRGWPTPLLRPGTLSADLATALDAPFFCGGLWGGGTAGG
WPFGEGADWAPRADMTVSADGSSYEWFVELPGMAKEDVTLSIDGDVLTVRGEKKTTRA
VMGGGTERVWGTFARSVVVPADGDVADKGAVRAVAKDGVLTVSVPKVAPRSKADAPEE
DNTIPIHGE
>PhuHsp20-19.0
MELLAMDLFHPQAVAPRRDGGGDGWGLWRPMTDPAWAASLSRWQPHATVEHDEAAKE
MAVRIETPGFPRDRLSVELSDDRRMLTVSGRMRKSEATPADGAADGAAAANGCGSGGAA
ETALSSFEERQFTQSFRLPRDADVEAIKADYEHGVLCVRVPTKGPEALPQKRNIAIADK
>PhuHsp20-18.9
MELLAMDLFHPQAVAPRRGGGGDGWGLWRPMTDPAWAASLSRWQPHATVEHDEAAKE
MAVRIETPGFPRDRLSVELSDDRRMLTVSGSMHKSEATPADGAADGAAEANGCGSGGAA
ETALSSFEERQFTQSFRLPRDADVEAIKADYEHGVLCVRVPTKGPEALPQKRNIAIADK
>PhuHsp20-28.5
MPMAGLVAPRLQLPARCVAITATDWSIKEQQPRTFVHWPYTNHPNTHTPPTSRQPSNPPFL

TYRGRPHFAPPTHPPPPRAPAMDVFALDLFHPPAPPQRRRDRAANPWGLWRPMMDPSFM
NALSAWQPHSTVCRSDDGKALNVRVETPGFPRDHLTIELSDDRSLLTVAGAMRKETGAEA
GATGGGDGGGDAAAPGTYTGVEARQFSQSFRLPRDADVENIKADYEHGILCVTVPTKGA
DALPQKRSIAIEDKTPDAKGTE
>PhuHsp20-20.1
MAAMSGMAADFVRTYGAFGRPPMDGRCGPAGRQQQQQQAQQAQRRAPSSPSPQQAR
VDWVPLAERAETATMYVWRLEVPGVTKGHVKTELKGADRVIVISGEKARPSAAAAAVAG
GVAAAAESEEARTVRGELTYGTFARTLRMPADADLDATDQIRAAVKEGVLTLTVAKVKAA
PEPEAEPPVEIPIM
>PhuHsp20-20.5
MEVLAADFFAPWAAPVRGSGCPRRMMVHGGGGGAHPPPPAWRPAAHVGRDEADGSVV
LRFETPGFPRSALGLALSDDSSVLTVSGKLLGKRKAPAASAGAAEPTAAAAAPAETTKETE
AAPVPAPATPAAAVTDASAGAAAVDEDAASADGAEVLKSFEKAYRLPRDVDAELVAATYE
DGVLTVRVPKKKRHAERRTIPIIG
>PhuHsp20-37.5
MNEPPSAYPTTRLPDVAAISYFSNLLLNPPHFLFSSTPPYHGGNVTLRHPLHHGVCLSSGRH
RLCPCRRRRAAADDHPPPARPNGVDPLSGPHVWRRRPPPPPTDAQREAWRRQVAAFGAA
AASAASSPDVQAALTKMGQAAAAAAGSPAVQSAMADMANEFLRNAEGEVDGGGGTPW
ARPPPPETTRVRPPTSSQPATPPPPPSPSPSRSPQPQPPSPPAGASTAPWPAWGVPTPAGRVD
WVPRAELAETPTAYTWRVELPGVAKADVRTELRAADREVRVAGVKRRPGGDGGGGGGW
PRGEVAYGSFGRSLALPADADVADRSSMRAAVRDGVLTVSVAKVVAEAVAADEAGVDIPV
V
>PphHsp20-25.9
MAFVSAAAAAGLGLRAPAVTAAALGVAARRVRLVPLPATRVRMTSAATPHDAKDEDADT
KSDAQASPRGHAPVRRRRGGGRGWRGVPSLWGLSGMRPDLIAGELAGAFDSPFFRAGGG
LAAGGWPSMAGAVADWAPRADLTVSADGKSYEWAVELPGMAKEDVKLTIDGDVLTVRG
EKRSEREVLGVGTERLWGTFTRSVVAPADADLDKNGVKAVSKDGVLTVSFPKTSPPEEHA
DKEDNTIPIIEE
>PphHsp20-19.2a
MDLFALDFFHPPAAPQRRGRAAADPWGLWRPMTDPSWVHSLSTWHPHSAVGRSADGKE
LTFRFETPGFPRDRLNVELSDDHTTLTVSGTARTETAPADAAGGDAAGGAERRPWASVEE
RQFSQSYRLPRDADVEAIKADYEHGVLAITVPTKGADALPQKRSIAIEDKDPAKESK
>PphHsp20-19.2b
MDLFALDFFHPPAAPQRRGRAAADPWGLWRPMTDPSWVHSLSTWHPHSAVGRSADGKE
LTFRFETPGFPRNRLNVELSDDHTTLTVSGTARTETAPADAAGGDAAGGAERRPWASVEE
RQFSQSYRLPRDADVEAIKADYEHGVLAITVPTKGADALPQKRSIAIEDKDPAKESK
>PphHsp20-19.1
MDLFALDLFNPPSAPQRRGRTVDPWGLWRPMTDPSWVQSMSVWQPHSAVSRSDDGKML
NIRFETPGFSRDRLNIELSDDHTLLTVSGSMRKETPAADGDAAAEPGAAQRNPWASVEER
QFSQSYRLPRDANVEAIKADYEHGVLAISVPTKGAEALPEKRTIAIEDKDPAKA
>PphHsp20-28.5
MAFVVTAVGVGAPAARTLGRPATAVKGRPLAAAAVTPRMTIRGGYPSADAQREAWRRRN
ASRAAAAAQGRRVGAPGRGPTQEELMETFSGMAAEFLRNSGAFSGPFAGGRCGPRAGPT
PGEWGAQQRRRQQQRRQQVDWVPRAERAETPTSYVYRLELPGVGRNVKTELKAADR

LIVVSGNKAPPAETREEATDAEAPAAEADVRNVRSELVYGAFSRTLRLPADADTSAKDEIR
AAVKDGVLSVTVPKVKAEPEPPAEEAVDIPIA
>PphHsp20-15.6
MADPSFVRSMATWQPHSAVSRSDDGVTHIRIETPGFPRDRLHIELSDDRSHLTVTGTAGTG
TAPPDGDDAADKTGAVGDARGPWAHVEERQFSQSYRLRDADVEGIKADYEHGVLSITVP
AKQRNMVPRTIAIEDKVAPKDSK
>PphHsp20-20.8
MEVLAMDFVSPWEVVAAPFGGGCGRRGYRVDGCRSAPSSAPAAAHTRRAPAWRPVSSV
GRDAADGAVVLRFETPGFPRDALRLDLSDDATRLDVSGKLTRDGKAPSTRPAVGDAAAA
DAADAADAADAAGAAAGDAGAKPASAGRGGAGAGPAAADAEVVKSFELAYRLPRDVD
AGAITATYELGVLTVRVPKKAVVAESRAIPILD
>PphHsp20-31.9
MAFVAPAVRVGTPAAPPLPGRPATTPPAGRRPAAAVTPRMTIRDGYPSAAQREAWRRRHAS
GAAAAAAAAAAMQRGPAATPGAVASQEALAEALSGMAAEFLRNLGECGGGPPPPGGRR
GPRASPAAGECAAGPQQQQQQQQQQQQQRQQQQRQQQQRRQRQRVDWVPRAERADT
PTAYVYRLELPGVGRGVKAELHAADRVIVVSGRKAPPRAEPTDAAAADTAADAAADAAV
AAAAAARTVQSEFVYGAFVRTLRLPADADVAAAGGIGAAVKDGVLTVTVRRVKAEPEPP
AEAPVDIPIMERGAE
>PyyHsp20-25.9
MAFASAAAGLGLRAPAAAATALGVAVRRVRLVPPHASRVRMTTAATPKDDKNEGADTQG
SMQASSRGQAPARRGRGRGRGWRGVPSLWGLSAMRPDLVAGELMGALDSPFFRTSGGG
GLFAGGWPSASGVDWAPRADLTVSADGKSYEWAVELPGLSKEDVKLSIDGDVLTVRGEK
RSEREVLGVGTERMWGTFTRSVVVPADADLDKSNVKAISKDGVLTVSFPKTTQHEEHAE
KEDNTIPIMGE
>PyyHsp20-19.2
MDLFALDLFNPSAATQRRSRAVDPWGLWRPMTDPSWVQSMSVWQPHSAVSRSEDGKLNI
RFETPGFPRDRLNIELSDDHSVLTVSGSMRKETKADEAGEAGGAQTQRNPWSSVEERQFS
QSYRLPRDADVESIKADYEHGVLAISVPSKGAVALPQKRTIAIENKDPPQVTE
>PyyHsp20-19.3
MDLFALDLFNPPAAPQRRSRATDPWALWRPMTDPSWAHSMSVWQPHSTVSRSEDGKML
NIRFETPGFPRDRLNIELSDDQSVLTVSGSMRKETKADDAGEVGGSQAQRNPWSSVEERQ
FSQSYRLPRDADVESIKADYEHGVLAIAVPSKGSEALPQKRTIAIENKDPQVTE
>PyyHsp20-19.6
MDVFVMDLFHPPTAPRSRGRNADASSLWQPTAASSWTQNMSVWRPHSAVTRSDDGATHI
RFETPGFPRDRLHIELSDDRSHLTVSGTARTKTGPANGDGDASAKDGAGDGKARGPWASV
DERQFTQTYRLPRDADVEGIKADYEHGVLAITVPAKHADMLPQKRTIAIRDKEDAKVAKQ
>PyyHsp20-37.6
MRPSRTVRDGHSRENRIKPPSCALLFTAPRLSTSSHPMRTHFVTHYHTLLLSAPAAGRVHR
KTLPRHHPHSRRRNLLSSTMAFVVTAVGLGAPAARQLGRASTTVTEGPPLAVAATPRMTF
RGGYPSADAQREAWRRRAANRSAAAGGQPQRRPEGPSQAELMDAVSGMAAEFMRNYS
AFGGPLAAGRCGPPAGQATGGRGARQQRQQGRQQQQQVDWVPRAERAETPTSYIYR
LELPGVGRNVKSELKATDRVIVVSGEKVRPSETANASADAAAPTSKVDVRNCRSELAYGS
FSRTLRLPADADVEAKDQIRAAVKDGVLTVAVPKVKVEPEPPAEEPVEIPIM
>PyyHsp20-20.3

MEVLAMDFVSPWAVTAPYGGCARRGHYGDGCRSSSVAPTRARSAPAWRPRSSVGRDAH
DGSVVLRFETPGFPRSALHLDLSEDATRLNVSGKLTREGGKASPPTVDGTAAAAADSPAA
AEGAATAGASGGVAGEPAAAASAGGGAITTDTEVVKSFELAYRLPRDVDASAITASYEFG
VLTVQVPKKAVVAESRTIPILD
>PyyHsp20-37.4
MAFVPAATAAAHFRCARRIGNAALTAGRPFRPRVVPAVRACRCVGRDDDKGDGFSGGGG
SGGGRAASGVDDSSYGGTTPQPPNHRREAWRQQAAAFGEAVIDAAGRGDVAAALGEVG
AAVAEVAGSSDVHAAVAEMVAELFSGQPRDDGQHSDTQGDDSPQRPPSSTRTAEASASAG
TARQTQASVANSAPRRRPRQRDWPQQVPSPLRAAAAAEQLLLPPPTQGVETTQPPPSPPPHH
ASAGWAPPAECRETSSSYVWRIELPGVARADLRVSLVPASRSVWVSGVKPRPAGGAWVG
VKAGAPQSAVGSSEVLYGAFRCRLPLPLDADVAAVPHGAPASLRDGVLTVTVARLPPPPDT
VFDIQVV
>CacHsp20-26.2
MRHATSNPAFLSVAPLRLGPSSPHLAARQSLWLGGSAPAVSRATPRRPFTTARQVVAAQQQ
QQQQHQNQRGSNGERGQRALQRRADWPFGIFRRDPFFTSPFTELDRFWSDFDALAQRQS
TYIPALDINETPDAYMVSVELAGVPKENVKVTLKDNVLNIQGEKKWEHEESNPTTHRSER
SYGQFSRSVRLPEEVVDQNGIKAQFKDGVLKLVVPKVQRPEAQPKEIPIEAE
>CacHsp20-13.4
MIAPRMDFKELPDRYVITAEVAGVPREQIKLEMHGDLLTIRGEKKEEHTQEEKDKDGRVV
YHRTERAFGSFERSVKLPKNIDKQSVHAACKDGVLTVTVNKAKRDEDERMTIDVAEA
>AcHsp20-27.3
MAVSKEKVAAKDSANDEEPVVYTLDMPGVKHENLKVTLHNNSTIVIKATRKVGGNEKY
YERMLRLQRTQLLDVTKLQAQLLLGVLTITVPKKDDLQPVLVPVAASYPPEVTESEDNNL
AEFTLDLPGVKAADAKIEVSVEGIVTITAEHKNRSYNRMARFLQPVDRHSLKAYLIDGVL
MVRGYMLHKSTATDLQEVIAVSTTEQPQIDTKMVESSKGEQDVVVEAKSNDADEVDGVV
VDVNEEDDNN
>AcHsp20-22.7
MKFSIPALTTLALVPTASAWSNSVFFGPGHRLGTSADTLLYPSDILAMHQRRKQKFFDNAL
TRISPRYEIQDTEQQIQISLDVPGVSPEDINVTVEEDGKVLAITAQREKIGDNGKYTNSVSQS
FSLDPVVDIEKFSANLKNGVLVVTAPKDLARLEKNIRKIPVAAVSEDVETPVEAKETIAPVE
DTNVDTEEPQDKDKVDGTAPAS
>AcHsp20-12.9
SLDVPGVQAEDINVTIEDDGKVLSVSAQRQKVGENGSYTSRFSQSFALDPTVDIESFSANL
ENGVLVVTAPKDLQRIEQNIRKIPVVQGDGVSSSFATDEVVAETKDDAATEDGDDLEIID
>AcHsp20-14.7
RVRASDGTDKRVPNYEYHREQDKILLEVELPGVQKEQVGVEMRGSNLCISGRRFKASGLF
VEEHKDDAADNGGTDSKVEEGKSASMIYELQVKVGDSVDENRIKAEHKDGLLKMMIPL
KTHPGSRKIAITHG
>AcHsp20-14.9
MGGPEFRIASTRGYDSMRTFDMTPLYRSTVGFDRMASLIDRALTADNTSQSYPPYNIEKTG
EDAYRITLAAAGFGEGDLNIETHESQLTISGAKQDAEPAEGVEILHRGIAARNFERRFQLAD
HVKVVSATLEHG
>AcHsp20-13.8
SKEAANANAKRTSLCGDFRTNQAKNGALQLTFDVPGLKETDLQVTLSRKNVLEIRGETK

NRRFLKTTTVDDETTDLSKLSAQLADGVLTVTAPPKETPKAVSFTVTTNDIEKLEDNLDEK
IMRFF
>AcHsp20-17.6
MEPLFPFSFSNREIHRLRQLTDQMENSFTASDGSQHHVPNYEYHQDGDKIHLEVELPGVQ
KEHLDVEMRGSNLCISGLRFKTSVVHIEEQKGDVAPTAEKDAEKKEGIVSIVYELQVKVG
ANIDEGGIRAEHKDGILKMLIPLKTETAPLKIAITQG
>AcHsp20-21.7
MPFFAPARYSDIYRPSTWTSPFGDDFFSMRPFDALLFDHPRSSLDNSEGFSSAPSFHVKRED
GKLTVEGALGSKYTPEDVKISLKNGNLLVEGKLTVEREDKSSGYTSMSQSSFSQVIPMPAD
ADLESLEASIHPEKKNLLVISAPLKALEHTESSAPVPLHITRDDPVSIEVEHDDEQPMNDDE
EIVEIGREEH
>AcHsp20-13.0
FSITSTKPMFFLRHNPSFRHGFFEPSTVLMSHSPTFPSVDDKGEAKSNDEPKKVYRSIDAHA
NESDDKFTLTMDLPGVKMDKVTVQLDEGTLSVSADRDTTNGAVVAYRQRFSLDQ
>MeHsp20-13.4
XLPGLTADDVDVHLDDGVLVVRGEKKYDGDAAERDDEQQHYSERSYGMFERRLRVGDG
VDESDVSATLDNGVLTVTVAAKXAAAAAAAEAHHDRHQVGVVAVRAVRQRSRWRCCIE
LVLAAVR
>MeHsp20-21.4
XSAMARGVKSGPLMSYGPRHRAYWRRHAFRPGSISPWTAPSGSISPFAPLVKEMLGSDVF
SAMENASRAFVPHGQVTENDKEYSLRLEIPGFPKESVKVDVKGKYLTIKGDVSTTSNDEP
QGEATEDSTEKEGAEEIQSSWERTAFYSKFERSFRLPNNAEIAGIAAEVKDGILLISIPKKQP
EEPETIPINIQ
>MeHsp20-24.8
MATLARVRPALRAMATIARRPGRGGSSSAWQNANLSQGNAYRSMLLRRDFTRMGFSPVL
EHLASKGPLSLLEMQARMAQGPHRAVFQGKSCFWPEKGLTAGSARLRSQMPCEVVEGSH
AYTLTAELPGVPRANIKVEVRQDDVLVINAQNQRTNDKGEVVFTAEFHREIRLPSNVDKTA
IRASTRDGILSIHLGKRPVENIVIEVADEAKQEIPTVHARPIDTQA
>MeHsp20-18.6
MDLVLGNYGFGPGSLWASPFATRNWMADFDKEFSNWPDRTRMPSYNSAVDDNGTIHLEV
ELPGIPREDVTVEVSGDRVLTIAGKRSTVKTWGKAEEPAEEAKPNGNPDEKQGEPAGKSES
YYELKRSFTLPTTADSEKLDCKLENGVLRMSIPKKEEKDTTMKIAIQ
>MeHsp20-18.5
MVCTTTYYFNPLEGVVYYPYGETKREAERESNVAPRYEERVDESTGTIHLEVELPGVKRE
DVSVEVRNYGKNLEIAARRARPGHNAEAQREGKISEEVVDGVAKPKTPEEDKSRNAGYV
QLLRSFKLPRTADANKLQAKLLDGILYVDIPRAEEAAPRKIELQ
>MeHsp20-19.6
XKSKTKDKRKKRMVCTKFYYYDPVDGALYYPHRVTKRETSRKLAPRYQAQVNDETGVI
YLEVELPGVRREDVAVELRNAGRDLEITARRARLSPKAQAATDERAVDGEPSRAETKENE
GGNAPGTKFVELRRHFSLPRTADTAKLEAKLLDGVLRIEIPRLEEASPRKIELN
>MeHsp20-19.7
MAALSRTFGGHSEPFHTPLWPWLEPHSLELPHGRSLLDPMAHMFSRNRFPGVEQHKTRD
GDICIAVDVPGVRKEDIEVHIRADDRLITIKATRRPENLWKSERVKTENGQVGGAEEEEAE
TVEGEAVEYERAFRVPGTVDLHRAEVSLQLGVLKLVVPQLHQSSEPVKVAVKSED

>CocHsp20-21.6
MDCGFVAGSLPVVSRGTNRSSICPIVTAPKGLNVQCVGGMRPHSNLARRRLDPMLSPMM
GFPFGSLMQEFADAMEKETTWIPRGDFHETEKEYFLRLEIPGFPKEKVSASIEDGVLTITGD
MNRNGKDAADNTEKDVEDPRPIESFYTKFTRSWRLPKNVKKESIRAEARDGILTVVIPKL
APEEPTIIPITVN
>CocHsp20-13.6
MAPFEDHVDENGDIHLTVDMPGVRKEDASIEVKDGEVIIKGTRHRHSARRVKENGSDAGE
KKGESGNAPREGTTSLQYFSRFAVPRNYDATQIKATHEHGVLNVFIPRRPESKPIQIPIESA
>CocHsp20-17.5
MYGSDLRMSPRFWGPTFSFFEDTDRMLDKMMTAMAPFEDHVDENGDIHLIIDMPGVRKE
DASIEVKDGEVVIKGVRHRRQKTKEAEGNVAERKDGADQDTSGATTSFQYYSRFAVPRN
YDAAQIKATHEHGVLNVFIPRRPESEPIKISIESAQ
>CocHsp20-19.6
HNSLNCCGRRVSTLEMALSSRVFGDWGMSPKFWGPTFSFFEDTDQMMDKMMTTMTPFE
DYVDENGDIHLVLDMPGVRKEDASVEVKDGEVIIKGTRHSRKTQKADGSSGTGGDRTEG
SRKGSSETTTSFQYYSRFAVPRNYDGTRIKARHEHGVLNVIIPRRPESEPIKISIASE
>CocHsp20-22.2
MAVLRTVRWGVPMKNEGSRRRKSRDWEDVEMEEQEQEQIKWEEPKWQRRLDENGVVH
LWIELPGVKKEDLSLEVKDGCLSVTGERSKRRKVQLMEGEVVVEDGDELCAEGGRPDTD
GSPIKKGKSSEPMVKSEEREKGTKEKEKSVPSPTEKTYHAIIKLGKLADTSKINATLRHGLL
TLTVPQVRPTPIQIPIS
>CocHsp20-18.5
XCQTMVFYSSPGGIFVSELVCPPSWDKLTNGGKRRATSECLTGAERILAPRVDSSIREDGTV
DLWVDMPGVSKDDVTVEATGAELTVSGSRRRIRPNIEAAIGVSGENGNTDAKTQKGAHQ
DRPVNLRYSTTIKIRNTMDLSRTSATMEDGVLHLAIPLKPEVAPVRITVT
>CocHsp20-19.1
XSRSLQLCQTMGFYSRPGGILVSELVCPPSWETLNYGEKKRAANECVNGAERILAPRVESS
LREDGTVDLWVDMPGVSKDDVTVEATGAELTVGGSRKRNTPNTEAANEASGDNRDTKV
KEGAHHDRVVNLRYSTTIKIRNTMDLSRTSAIMEDGVLHLAIPPKPEVAPLRITIA
>CocHsp20-7.6
XASGHIGDTKVKTQEGAHHDRMVNLRYSTTIKIRNTMDLSRTSATMEDGVLHLAIPPKPE
VAPLRITVA
>EaHsp20-26.5
XDSVLRARAMAFVAGIVPVTKQGSTVASCNSFVAPKLTAQVKPLAAESVVMSMMYGPRS
YRASRARASAPTALHMSMLGPYFGRSVVPRKQGATPSPIQLLNEAERMLTNPFEFFDLATR
EAMWTPQYGFDENEKEFVLTIEAPGIPKEQISLEIKDDILTVSGGMETKEKKEGEKGQVES
FQSRSFRRSLRVGSGVDQEKIRAAVKDGIIRVRMPKIEEEAVKTRKIELEEEHEVPPQ
>EaHsp20-30.4
FEQVVAYKTDAVAREPCVGREHKATDSSACVRTERRKIQANVTEEMAFVGGVVPVTKQA
SLSASCSSFARGDGAVQPVSMRRVAAGGVSMMFGPQYYRGPRARVCGPTAFELSMMAPL
LGLGLNMAARSQSSAPSPVQSVNDVERVMNNRCDPFDSMWQPRYKIDETETEFVLMIEA
PGIPREQISLEIKDDTMTVTGGAPEQKAEEQKDGDAATQSRSFQRSFRRSFRVGPQVDKDK
IRAVVKDGIVRVRMPKIEQEVVKTRKIEIEDDSDIPAQ
>EaHsp20-25.9

MAFVSGAVLANKLASSAASCSEFAGAKVAMQPAALRRVATSGVSMMFGPQYYRVPRGRV
YTPVPIELSLLAPLLGLGPSVAYRPQRSAPSSVRVMSDVERKMKQEYDMLRTTWQPRYKT
EENETEYILLIEAPGVPKEQISLELKDDVLTVTGGMAEMKEREQARAGVTSRSASLERRFR
RSFRINQHIDKDQIRAAVKDGIVRVRMPKIEHDVIKTRKIEFGDDSSFPQQ
>PoaHsp20-15.8
MALSRRYPMPTDLWYEIPRFESLFRPMELENSAFGAMNSYTTKDGTAVVHMDLPGVKKE
DVVISHEHGMVNIKAKRQTKFEDEKVTEESYSEITRSFMIPDKVYDVEKADATLVDGVLKI
AIPKLPENKQAKKSIAIQ
>PoaHsp20-15.6a
MAVSRRYAVPMDLWFDTPRLESLFRPVELETSSFGAINSYVTKDGTAVVHIDLPGVKKEDV
TISHENGMVTIKAKRQINYKDDVTFKQESFAEVSRSFTIAEKVYDVDKSDASLQDGVLKIS
IPKKEQSKPAKTSIAIQ
>PoaHsp20-26.9
MAFVFGVPTIARPAVTIAPRQAPTMSLMRPNWNAPIRSRSRVDPWLVPFPAATDISTIFEEM
QKMMVPFYNEMEQFHGLLEKENWAPKFELKEDETDYHIRIEAPGIPKEHIKIYVKDNVLH
VSGDSTQVTGHTEQDKEKEVVEEKKHDIKAEVEHAANKDEEKKVENPKLAKEEVAAPLA
KQYTSTYVQRSFNRSFPLGKDVNADAITASTKDGIVHIKLPKVKPQEAAVKTINID
>PoaHsp20-15.5
MALSRRMLTPTDLWWDAPRLKSLFHPMELEVSPFGAMNSYETKDGTAVVHIDLPGVKKE
DVEISHEHGTVTIKAKRQTKFGDDDTTEESYSEITRSFTIPDKVFNVEQAEAVLTDGVLKISI
PKLPEDKAPKTNISVQ
>PoaHsp20-15.6b
MDLSRRRAPMDLWWDTPRFESLFRSMQLEASSFGAMNSYVTKEGSAVIHIDLPGVKKDD
VQIIHENGTVNIKAKRQTKFTSEEVDEESFSEIQRSFTVPERAFDVSKATASMNDGVLKICV
PRLPESEMQQQSISIQ
>PoaHsp20-49.3
MAAFIFVSPYLWKCSTLPISCEITFPHSKRMVQTNNNFFHGCSQRLYSKCDILSGARNRKR
SRNMGWFMAAAPIEMNERDEDDEKANVNRPYASPTDDAESTCANQNKDINSGQGAGAG
EDVEDDKSDSADGNDDDACKRSDRNPMGELNENEAELRKARVNEQQRSQSEEEQKESK
NQENNQDEDVGCSRAEQEERMRQKQQTESKWTAPESGGNGNKGVPKREEKKVATSPWS
GKTQEHTADNRSPRRGPGMSLRASSPLGAVDLWAPWLPFPFSKCCGSSRWSSGPYCWLDE
LDFLAPSYSISSYSNPWDYFSSRFPEIRLHEDPSRKEYTLVIEGPGFDNDDIEVFVKHRTVY
VRGGWNNEHGTELLGGRCRTLLSRRFSRSFKISNDVDVDRIHARVKHGIIYVHMPKEGMD
HDNEKGDCGKKIDIQQD
>RhvHsp20-21.4
MAFVSSSTLIPYRALMVPISVAAPKRFLCASGRKPCRAAFRTTSPVQMRTGSIVPNYSGIMS
TLLDPFPSLTRLEQELLGTGMNWVPLCDVSETADAFIIKMDIPGMTRDDVNVQVKNGML
QVSGEKVNEFKDENVCKMERRYGSFSRMTRLPENVDLDNIKAEVKNGVLTVRAMKKEK
DVEEEEGKVIQIE
>RhvHsp20-26.8
XFSTSKVAGTAKNGPIKARGPRPRHSSRLRVAALTRPPPSATPISSSNCHGFRFLKHAHPVQ
GLNGAHQRRCSQEVPLCFGEEALPRSFPHDLSGSDEDRLNCAELQGIMSTLLDPFPSLTRL
EQELLGTGMNWVPLCDVSETADAFIIKMDIPGMTRDDVNVQVKNGMLQVSGEKVNEFK
DENVCKMERRYGSFSRMTRLPENVDLDNIKAEVKNGVLTVRAMKKEKDVEEEEGKVIQI

E
>RhvHsp20-17.4
MAEFSPKDIIPRTSNIIDSVFNDDFFSPFLPLTTLGMPVTRGGGNLIPRVDVSETDKTVTVVA
ELPGIKREDVTVSVKGDVMTLRGQKKEMREEGAPGTTFHRMERSFGTFERSMRLPKDVD
PDKVNAKYTDGVLTVTFEKKDKKEMQGKMITIGE
>RomHsp20-31.6
XELDLFRLPTLFDSVRYRPRARMSQEETESEYKFTIVLAGVGREAVELKIVGKKLDIKAGE
EDSQFQFAQRFSLPKNAVHEEAKAVMKNGVLQITVPKQAEKAARDVIVQDSGEPGENDY
KLAYQVPGASKDNVTVTVNGNKLVIKVTAENNHYFRNFSDDYKIPKDASPEDITAICKNG
VLTVIVPKISPTSVAVEESVEEQEGSFSTSIRLPGIAKEQISLNRVKHAFKLIVEDSQRQYEYS
FYTPEEVDVEKVKAGLKNGILTISAPRVAEVEHVIPVESA
>RomHsp20-34.8
MALTIFRYPFEVDCHPRRHHRMRRTQSETEDEYTFNLVMPGVNRDAVKVEVVRRKLKISL
TKVEVVRRKLKISLTKDGASEAQWISLPKYADRSGITAKAKNGVLAITIKKKTRDPTKKID
LRDSIELIDSAYKLDYSVPGAGVDDVQVSITGARLTINAQSGSMDFFDDFRRTLSTPSDADL
ANVSAALKDGLLLIQIPKLEPVTVPVEKGQATESDELYVAYFRTPGIPAEKIEVTRVGRKVE
VEAKLSDSMKENDFYSSVETEFSESIYLPESVDLESTRAVAENGVLAITAPVLETERPREVA
VQSES
>RomHsp20-34.0
RRIICVKRDKCFETNANRMMELDLLQLPMLFDGVRYGPRVRTSQEETESGYTFTIVLVGV
DREAVELKIVGNKLDIKAGKKDGNFLFEKRFALPKNSVHKEAKAVMNNGVLQIRVPKQA
QKAARDVIVQDSGEPGESDYKLAYQIPGASKDNVAVTVQGNTLLIKVTAENNQYFRNFSD
DYRIPKDASPEDITAICRNGVLTVSVPKISPTSVAIEESLEEQEGSFSTSIRVPGIPKEKIILNRV
NHAFKMIVEDSQRQYEYMFYTPEQVDVEKVRAGLKNGILTISAPRVAEVEHVIPVEST
>RomHsp20-20.0
MRPMRFSPTRMGPMRMSNAFWTSPLFNEILNPLFQEMASSGYSDGNMWTFPRGEFINKE
TEYVVRLEVPGYAKEDINVEIVGDFLVASGKVQKTSEPAQEGETKVQAEPTEATPYTEEEE
PKAEQSVGWTTAKQFKRQFPLPKGVQREAISANVKDGILTITIPKSVSSEAPSTSIPIN
>RomHsp20-16.4
MSMSLWRCRGGSLMRHPHTRQVLMEMEQAVNTAYENGVSRGGFWNGMWRRQSIAEQE
HQFELKVELPGIPKDKINVERLGDQLKVFAEDKGADSLSRFEQVYNLPESADLAKISANST
DGVLTVRIPKTEHHIESSKPVNVKVE
>RomHsp20-13.4
GRAMTMSLWRCRGGSLMRHPHTRQVLMDMEQAVNMAFENGVNRGGVWNGMWRRQS
IAELEHQYELKVELPGIPKDKINVERLGDQLKIFAEDKGADSLSRFEQVYNLPESADLAKIK
>StHsp20-35.5
XKVDRGGVQWSQAYWEGETGGPGQSSTEDVKMEFEIYRFPAAFNVCSRSRPQPRYTTTE
TDDAYVISVVLPGVERQDMQVKRIGEKLTVNAAASGAGESCAFEETFSVPEDANLHDVK
AETSNGVLRVTVPKKAAELPRAIEVAAVMERDKESYELVYHVPGAGAEQVSVEVTGNEL
KVAAEAGERAFFRGFERTYTIPQDADANRIRAFCADGVAVVRIPKLAPVKLDLAGEVPTGE
DMYTVHLKLPGVASENITVERTRRDVRISVAEAEGERQIVHKHSLRVPRRVDPGQVTAAY
KHGVLTIGGPKAQFETKVFEVVAHNAA
>StHsp20-22.5
RPRHLQARLAXQNNVAQKPVTAVTMRMRGPAFNPLAASFAAPLFFEMMQPFMQPMMPS

GKNVQMQFNPRGEAIEKDKEYIIRFELPGFTKDDVKTEIKGDVLVVKGNRHEEESKEESK
EEQGDGENTGEAKSEQGNDEIKRRQSFGWSMQSSFIRSFTIPKDVDRQSIKAEMKDGILTI
NLQKQSKPEMETIDIPIN

>StHsp20-23.2

XKEYNMAFVSGFSAATKAKTLRLVESTPKKSVASQRAAPTMGMRARGPMYNPMAASFM
PLAAPFLFDLMQPMFTPNKNSYMRFNPRTEALEKDKEYVIRFELAGFSKDDVKTEIKGDT
LTISGEKHAKEAKEDEKQSSEDQEKQQEEVIRRQTFGWTMHGSFARSYMIPKDVDRENIS
AVMKDGILTVTLPKQAKPQSESTSIPIN

>StHsp20-23.6

MKTAFVSGLNTAIRPKRELLVHNGPTKAAHSRKGAVEMNMRRREAFDPFASMGMVPFSS
PFMSSFMSPMMMDLMEPMFTPMRRGNNMFSMPRSEAFDQEREYIIRMEMTGFGKENVR
TEIKGDNIVIMAEKMARGMPYDQESQTNNNTMQGQSFGWAMSNSFAKSYIIPEDVERQKI
SATMKDGILTLVLPKKDREEPAIETTSIPIA

>StHsp20-6.6

XLPGVDSDNIHMEVVENRLAITAKSSTPTSQYDYEEYFSLPEDANSADLKAETKNGVLNX
