# Supplementary material for: Heat Shock Protein 20 Gene Superfamilies in Red Algae: Evolutionary and Functional Diversities
Source: Front Plant Sci. 2022 Mar 16;13:817852. doi: 10.3389/fpls.2022.817852 (PMC8966773; doi:10.3389/fpls.2022.817852)
Supplement: Supplementary file 9 [file Data_Sheet_2.PDF]

Supplementary Data Sheet 2. The heat shock elements (HSEs) in red algal *Hsp20* promoters.

>*CymHsp20-27.1*

TCGTCTATCCGTTGGTTGGCGAGATCCAGACGATTCCCGGGCTGCCGACCAGGCCCGC

GTTCTACGATATCGATGTAGATGTGGAGACGGGGCAAATTCTGGGCCTTTCGTAGAGTT

TGCTCGCAGACGCTGTGTCCAGGCTGCAGCGATATGATTCGCGATGCGGACACGGCAC

TGCCCAGGAGCGGTTTTATGCGTCGAAGCGGATCGAGTCCGTTTGGGTGCCAGAAGCG

CACGTCTCGGAACGACACCTGGAGCAGCAGACCGGCGTGCTCGCAGACTCGCACCAG

GGCCACTGTGAAGCCGTGTCGACGCGCTCGTTCGGCTCTGGACGAGCGCGCCCGAGC

TGGAGCAGTTCATGAGCAGATCCAACCAGTCAGAGGGACTGGCTTGTGTTCCGAATCG

CTATGCGAGCGGACACGGAAACGCATGGAGAAACAGGCGGTCCAGCGCATAACGAGC

GAACTGCACTGAACCAAGACGCATGTCAGGCGGAAGGTATCTAGGAAGGGAAACCCT

ATAAATGTACGTTGCGTTTACACGAAAACTTTGTACAAACACAAAAAAGACCTTACAC

TATTCAGCGATCGCTCGCACGAGCGCTGCACCCCGGCGGGATGCTCACGCCTCGGTCA

CCTCAATATTCATTTTCTCGTCCTCGTCCTTCTTCAGCTTATTGATGACGATGTTGAGCA

CCCCGTCCTTATGCGTGGCCTTGATGCTGTTACGGTCGACATTCTTCGGCAGCTTGAGC

GAACGCTCGAACGCACCAAAGGCCCGCTCCGTACGCAGGTACACAACACGCCCGCCT

TCGTCTTTCTCCTCAGCGCGATTCTCCTCGCGCTTCTCGCCGCGAATGGTCAAGAGGTC

CCCGTGCAGCTCCACCTTCACCTGATCGCGAGGCACACCCGCGAGCTCCGCATTGATC

TCGTACGCCTCGGGGGTCTCCTTGAAGTCCACCCGCGGCGTTATCATCGCCATGACGTT

CTGCGGCCTCGCGACCACACCGTTGGCCTTGGCCGGTTTCTGGTCATCGTACTGCTGTT

GCTGTTGCTGTTGCTGAAGAGCAGCTTGACGACGCCGCGAGACGCGCGGGAACCCGA

AAAACCAGTCGTCCATAAGGTCCTCAAACGGATCAAAGAAGTAGTAGCTCATGGCTCG

CTCACGTTTGTTCGTATTGCTTCGCACCGTTTTTCCGCGCTTGTATCTCACTTGCCTGGT

TTCGCACACTGGTCTGTGTTCACGATGGAGAAACACCAAAGCGTAACAAAGCCATGA

S-type

ATCGACGATCAGGCGTGCTGCTCTTATAGCTTACGTGGCGGATTCGCAGCGATTCGCGA

GATTTCCCGAATCGCCGATCTCGCGCGAGATCCTCGGCGAAGATTCGCGGCGCATCATC

GAACGGATCGCAGATGCTGAAATACCGCGCGCAGCGAAATTTTTCCAACACTAGATTT

CCATTTGTGTTCTAGCCGTGGAAACCTGTGAGAGAACCAGGGATTC

>*CymHsp20-20.6*

GGATCCCGATCTGGCGTTTCGCAAAGGCCTTGCCTCCAGACCAGCCTCGATCGTCTTG

GGAAGGTTCGCGGGATGCGTGAAAACAGCGGCACACGCTGCGGTCGAGATGCCGTGC

TCCCGGCTCTGAAGACGACGCTCGTACCGGACTAGACCGAGCGGTTGCCAACAGCGC

AGACAGGCAGTCGAGCCGCGCACGCGGCAGCTGCTGCGGCACGACGACTCTGCGGAT

CGCAGGGAATCCGCGCCAGCAGAAGCGACCTATTCGTTCAACGGTCACGACGCGTTTG

CAAACTTGTTCAGTACCGGACAATGGTGCAGATGCTCATGAACTGCGCATTCGTCACTC

TTTACAACGAAATCGAAAGCCCCTATGTACATCCCTTAAAAGCGCCTACGCATCACGGT

TTCATGCGTTAGCGAGACTGCCACTGCGGATCACGACACCTCAATCGGAATCTCCTTCA

CGTTTTCTTGTTGCTTCACTTTCTTCGGAATCGTGATACGCAGGACGCCGTCCTTGTGC

TGCGCCTTGATATTTTCTGCGTCTACGACGTCCGTAGGCAGCCGCACACTCCGCGAGAA

GGAACCATACGAGCGTTCCATGCGATGCATCTTGGCGTCCTTCTCCTCGTGCTCCCATT

TCTTCTCGCCCTGAACAGTAAGAATATCACCATCGAGGGCGATCTTGACATTCTCTCTG

GGTACTCCGGCGAGCTCGCAACTAACCACAAACGCATCATTCGTTTCCGTGATATCCAG

GGCCGGCAAGTACGCATTACTGCGCTGCGCGAGCGCGTCAAAGTCCGACCAGAAACG

ATCCAAGTCCATCAGCGGCGACATGAAGAAGGGATCGCGGCGCATGATGCTGAACGGC

CAGCCCCAGTCCCGTCGTCCGCGAACCAGCGTCCGCTCGTTATCACGTTCGCGACTGT

TCGCATTCGCTTGCATGAGGCTCGTTCGTGCGCTGCTCAGCGAACGCTTCGCAACACT

GCGAGCGAGCGCTGGTGCATACGGCGAGCGCCCGCCCACTGGGGTCATCAACGGACG

CCGCAGCACAACAGACGAGCCAGTGAACGGCACGACAAAGTTCAGCTTTGCACTTGC

TTGACCCAACATTTTCAGGTTTCCTTGCAGTGAGCGCAATACGTATTGCATGAATCCCT

GGTTCTCTCACAGGTTTCCACGGCTAGAACACAAATGGAAATCTAGTGTTGGAAAAAT

TTCGCTGCGCGCGGTATTTCAGCATCTGCGATCCGTTCGATGATGCGCCGCGAATCTTC

G-type

GCCGAGGATCTCGCGCGAGATCGGCGATTCGGGAAATCTCGCGAATCGCTGCGAATCC

GCCACGTAAGCTATAAGAGCAGCACGCCTGATCGTCGATTCATGGCTTTGTTACGCTTT

GGTGTTTCTCCATCGTGAACACAGACCAGTGTGCGAAACCAGGCAAGTGAGATACAA

GCGCGGAAAAACGGTGCGAAGCAATACGAACAAACGTGAGCGAGCC

>*GasHsp20-23.0*

GCTTGAAACAACCAAACATGTACTGGAATGCCTCCTTTCTGAAAAGCGGTGAACGTTG

AAGACTACGTCCTGCAATGAATTCTACAAACACTCTTCTTGTTCCACATAGTCCTCTTTT

CATTTTATAGGCTACCAGTAGTTTCTTTTCTTCAAAAAGAAGATAACAACAACAACAAC

GAAAACCACGGACCAAAAGAACCTCCATAAAATAGCCTTATAATATGGACCATTCCTCA

CTTCATTGAATATCTCATCGTTCCACAAGAAAATAGCTCTTTTCTCTTCAATAACTTCAT

CTTTTTGTTCTTCCGACAGAGTTAGAGCATTAACTGTTTCAACATACTTCTTAAAGAAA

GCATCTGTATCACTAACATTTGAAAAGTCATAAATTGCGAGGCCCTCTCCAGAATTTTTA

TCGAGAGCCATGGAAACTACCAAAATTTTACGGAGAACAGAACCTCCCCTGAAAAGC

GCCATATACCTGTTGCAAAATAAGAACAGAGAGGTTTTAGAACGAAGCACTTACAAAG

TTATCACATAAGACAGTATAAGCCAAGGTTTTGCGGAAGAAACCTGCCTAATATGATCA

ATATATCGCGTCACTGTCGGACTGGGTTCCTGTAACAATTGGCGCCAGTTGTCTCCTAG

GAAAAACTGAAGGTCCCTTTCAAAAGACTGCAACAAACTAGACTCAGTAAAATAAAA

CCACTGCATATTATGTTGACAAAGGTACCTTTTTCCTTCTCAACTCTTCAAAGTAAAGA

GCCCCTATTTTAGGAAAATTTCTTCTCTGGCGGTCAAACTCCTCTTCAACGGTGGAGTA

GACATAATAAAAGGACTCTAACGCCTTAATAACTTTAAAAATATTTCCAATTAAGGTGC

AGATTTTCTACCTTAACGTATACGCGACGGTCTGATAGTGCAAAAGGTAGGGTTAGGTT

TACCAGGGTATTGCTCACATTATGTTCTCTTCTTGTTTCCCTTCGCAAGCGAATCCAGAA

ATCGTCCATTTTTATTCTATTCCATAATTCGATTATCTTCCAATACTTCACGTTGTAAAAG

TAACCTCAGCGAGCAATTTTTTTGTAAATAACTTCTCTCGTTTGTTTCCTCTCTTCAAGT

AGTTTCAACACGCGTTGATACTTTGTCATGATTCTGTCCCCTGAGTAACTAACCAATTTT

TCAAAGCTCCTTGGAAGAATAGACAATTCATAACTATACTCACAAAAAATTGCAAGTTT

TCCAGCGTCCAGACAGAAACTGAAAAAATCTTTGCAAGAAATTTGTTCGATATACAA

P-type

GATCTTTGTCTAAATGTTTGCTGTGTTCTGGAAGATTCCAAATCTCACATGACTCTTCTG

GATTCTTCCTCATCAGATTTTTAAGTATGTAAGTTGCTTCATTCTTGCCTCATCTGTGGCA

AGAACTAGCTTCTGCAATAATGTTTGTGTATCACTGTGTACCTGCGAGAAACTTACAAG

CCTTGGCTCGTAACTTTTCTA

>*GasHsp20-20.4*

TTGCCAAACGGCCGTTTCGTATCTCCGACTCTTGATATCTGCAAAACGTTAAAACTGGT

TCAAAAGTAGTAATAAAGTTTTCGTTACCTCCTGCGTAATTGTGGAGATTTTGCAAGAC

CAAGGGGATCAAATCCAAAGTCACCAGGTTCACGATCAAAGGAAAACGTAGTACGAA

ATGTCGCAATTTCAACTAGACTGATAAACAGAATAATTTGTATCCATCCCTCTATTGGCA

CCTTGTGAATAGCCGCCAAGGCATGTTTCTCGTTGAAAACATCTCCCGGAAGATGAAA

AAATTCTTGTGTTATATAGCCTAGAGCAGCAAGCATAGCCACACGACAGTGTTTATTTC

CTTTGTAGGTTAAGTAGAAGATCGACAATACCCTAGAGTTACCTACAGAAGCTCGAAG

ATAATCAAGATTCATGTTGTCAGAAATATAAAGTGGATCAAAGCTAACAATCCATATTAA

TTCAAAGCACAACCAGAACGATGCTTACCCTGCATCTCCAGGAATCTTTCCATCGAGCT

TTTTAGGTGCTTCGAGAAAAGGAAGCGCTCTGGACTTCACAGTCATATGGAGTGAGAA

GATACTCTTATTCACGGACACATGCTGCAACTTGGTCAAATGAGTGAAACTCTTTCTCG

ATACGAAAAATTTACATAGGCTAGCAAAGGCAAACATTGTGTACAAGATTGTAAACTG

AGATAATCTTCACCAGATTTATCTTACTTTGGGTCAATTTGTGGCTTTCGGCTGCAGAGA

TTTATTTCTGACAGTACATTGTTCTCTGCTTTTATCCAAATGAACTCTGAGCTGAGATCA

ACCACTTTATCTGCTTTCAATATCCTTGTTTTATCGATTTTCCTATCAAACGCTTGCTATT

GAAACATCTTTTCAAAATCCGTCATATTGTCTACTATTTCCACTAAAGTTTCAACAAGTC

TTGTCAATACGAGGAATTTTAAGAATTTGCAAAAGTTAAGTAAAAAGAGGAAGAGAAC

TATTGTGCGTATCCCTAAGTTTTGTTCCAAGGTTGTTTCATTTTACGTAGCAGCAAGGTA

P-type

GCAACTTGAACAATTTGTTATTTCTTGAATGTTCTGGCCTTCAACTTTGTAAGAATAGCT

TTCTCAAGAAAGTGGACGAGCCTTTAACGTTTCATTGTTTCTAATCTGCCCAATGGCTA

TAAAGTTCTTTGCAAACTAAAATAATACTCAAAGAGTCATAGAAAGAGAAATGGTACA

GGTTCCTCAAGAAAGGAAACCATGGTTACCTCAAAAACATTCAGTTCAAAGCAAGGC

GCGATAAATTCTCATTCGAAATGAAAAGAACCATACTAATTTATCCAGATTTGTTTTATT

TGTTATTCTACATAAATATTTCTCGTTATCAAAGGTCACTAGAACATCAAGCAGAATCAT

P-type

CCACAATCTTCTCGAAATTTCGAAAATGACAATTTAAGGATCGCACTTGCTCAATAATC

GAATCTGAGGAACCAATAGAAAA

>*GasHsp20-18.0*

GTTTCTAACAACTGTTCATTTTGAATTGCATATTTACAATAAAGACGGTATTTAGAATCG

AAGTATACTAAGAGTGATTCAAGAAAGAAGAATGCAGCCTTGGTATGGGTGACTTTTT

GGATAATCCTGCTCTGATAGGTCTTACATGTAACAAACGAGAACTATGGGACGATTCCA

ACTCTGTTTGGTTATTCGACGATAAGCCACTGCGAATGGGCTTCACGGAAATTTTTGAC

TTGCAATTCGTATCGTTCTTCACAGCTCTCGTACTTGTTGCTGTTGATTTCACGTTTTTC

TTCTGTGTTGACTGATTTCCTTTAGCTTTGGACGCTCTAGGATTTGGGGTTTTCTTGGAG

CAACTCGTAGAGTCACGTCTCTTTTTTCCTTTTTTCAGCTTATCATCTATTAATGAAGAA

TCTTCTGAGTCATCTTCGTCAAAAGCACTATCTTCAGTATCGTTGGTAGAAAAAGATTC

GTCTTCATCGTCATTGTCACAATCAGGTTCTTCAAGTACTTTAGAGTCCCTCTTCCTTCT

TGAAGATGACTCCTTCATATCAGATACAGAGGCTTGTACTTGAGTCATTTTGCCTTGTGC
TTTAACAGCTGAACGGCGACTTGGTCTACTATTGAGTTCTTCACAATATTCTTGATCACT
TTCTTGCTCACCAGTCACTCCTTGACCCAGTAGTAGAGGAAAATTCTCCTTTTCTTCGG
TATCAGAAAGAGACAACTTTTCCGATGCACCTTTATTAGGAGATTGTTCTTTTTTCTTGT
TTGTTGGTTTTTGAGAAACAGCGCTTTTGTTATTTCCTATGGCTGTTGTTGCAAAGTCA
GAAGACTCTGAATCCGTATCCATCCTCGCTTATCCAAAGAAGAGCAATGCACGATGCTG
CTTCATTCCCATTTTTGAAAAAATGACTTTGCCCGCCAAATTTTGCTGTATCAAGGAAG
TGTTTATAAGTGAACCACTCTTGGAACGATTTTCTTATAGACTTGTTCTTCTCCTATTAG
GAATCACTTACAGAAAGGGTTTTACCCATAGGAAAGAACATTCACATGTCTGTTCTCCA
ATAACCATACATAGAGCTATTCACTCAACACTCTCTTTGGAAAGAGTATTGATAAAAAG
TTATCCCATTTGATTGAAGACAACCCATTTCTGTCACAACGTCTAAAATGATATAAACCT
AGCCCTCTGTATGAGAAAAAGAGAGAAAGTGCAGAGAGTGGGATTCGAACCCACGCC
ATCTGGGATGACTGCGACCTGAACGCAGCGCCTTAGACCGCTCGGCCATCTCTGCTTC
CATCCATATTTCTGTGGTGTTGTCGAATTTTCTAGTAGAGATAGATTTAAAATGGAACAT
TCCTTGGTCACATTTTAGAAGCATATAGATGTATCGAAGAAATTTCTATGCATTCTTTAA
ATAAGCATGATGAACTGCGTGATTGGATAAGATATCGAATTTGGCGAATAGTGAATAAC
TTGCATTGTCAGCTACC

>*ChcHsp20-16.5*

ATCTCGACGATTTTAACGACCTTATCGACAAAAGGCACGGTGACAAATAGCAAGCCGT
CGCCGCAACAATCGGCCTTGACGCACTCCTTATCCACGGACTCGCCCAACGTGAACTC
CAGGAGGTACCTGCACTCGACGAGCGACTCGTCCTTGTGTGTCCTGACAGCGCAGGA
CCCCGGCTCCCTTTTGTAGCGCGTGCCTTTGATGACCAGCTTGTTCATCTCTACCTGCA
CGGAGACGGCGTCCTGCGGGACGCCGGGCAGCTCGACTTGCAGCGTCGCCTTGATGG
AGGAAGCGCGCAGCTTGTAGCTCGGCTGGACCGTCGCGCACGAGTGGCACGAGTGGC
ACGAGCTCCCGCGCGCCCCGCGCCGCAGGTTCGCGGCGAGGGGCGACAGGTGCAGGT
CCCAGAACGGGTCTCGAAAGAAGGGAGTGTGGAAACCCATTGCAGCGCCGCGTGCGA
GCTGGGAGGACGTGGGCGTGAGCGGCAAAGGTTTTTTTTTTCTTGCTGGGACGCGAG
GGGGGGATTAGGGTGGCGAAGATTTCAGCTTGCCAAACGGGGTGGCCCTTACAGCGA
AGAGGTTCGATACTGCATAAACGCGCATACGGTTGTGTTTTGCTTGGGCTGGGGCGGTT
TAAAGTGATGGAAGTGGACGAGGAGCTGGGTTTGAAGCACATTACTGAGACCAGGCC

ACGTGGGCAGATGTTCTGGAACAAGGGGAGGGCGGGGGGAAAGCTGCGACACAGGG

AGGTTGGTTCCAGCTTGGAACGCGAATGGTACTGCATGCGAGGCAGATTGCCTCGCAT

GCAATGAGGTGGAATGGATGTTTGTGGAAAAGCGTGACACGAGTCACTTTTGCGGAG

AGGCGCGCATGGGAATTGGATGATGCTACTATGAGGCAGAAGCAGAACAGGACGACG

CATACATTCGGGAATGGGATGGCGCGCGTGTGCCGGAAAATGTCAGCGTTTTAGCTTG

GGCAAGGGGCGAAAGTCGTCGAGATAAAGTTTGATAAAGCATTGTCGTTTTAAGAGGG

CGTTTTGGGTTTCTTGACGACTCTGGGATGTGTTTGCCGCTTTTCAATCCAGACGTATG

GTCTGTTAGGGCATTCATATCGAGATACCTCTCACTAGTCGCTTTCACAAGCGGAAGAA

GCGGTAGTATGTGTAGCGAGATGTGGAATTTCGCGATGCGGTGCAATGTTTAATTAAGT

ATTGGAGTTAGAACGACCCTAGTTCTGCGACGTGTCCTGTTTCCAGAAACAGGAGTCT

GGAATCTCCGTCGTTAAGATTTCTAGAAAAATCTTATGGCCAATGAAATAACGCCATCA

TCCGCCAGTTAGCGCGCCACAACCAATCACCGTGAGCCCATTTTGCAAAACGCTCCTT

TTCTACCAACACATGCGCGTACGAGCATCGTTCTAGAAGAGCACAATTGGAGTCATCC

GATACTCCGCCTCTCGCTTCTGGAGGGAACGTGAGCAATTCAATAAACCCTCTTC

>*ChcHsp20-18.6*

GTCGCAGCTTTCCCCCCGCCCTCCCCTTGTTCCAGAACATCTGCCCACGTGGCCTGGTC

TCAGTAATGTGCTTCAAACCCAGCTCCTCGTCCACTTCCATCACTTTAAACCGCCCCAG

CCCAAGCAAAACACAACCGTATGCGCGTTTATGCAGTATCGAACCTCTTCGCTGTAAG

GGCCACCCCGTTTGGCAAGCTGAAATCTTCGCCACCCTAATCCCCCCCTCGCGTCCCA

GCAAGAAAAAAAAAAACCTTTGCCGCTCACGCCCACGTCCTCCCAGCTCGCACGCGG

CGCTGCAATGGGTTTCCACACTCCCTTCTTTCGAGACCCGTTCTGGGACCTGCACCTGT

CGCCCCTCGCCGCGAACCTGCGGCGCGGGGCGCGCGGGAGCTCGTGCCACTCGTGCC

ACTCGTGCGCGACGGTCCAGCCGAGCTACAAGCTGCGCGCTTCCTCCATCAAGGCGAC

GCTGCAAGTCGAGCTGCCCGGCGTCCCGCAGGACGCCGTCTCCGTGCAGGTAGAGAT

GAACAAGCTGGTCATCAAAGGCACGCGCTACAAAAGGGAGCCGGGGTCCTGCGCTGT

CAGGACACACAAGGACGAGTCGCTCGTCGAGTGCAGGTACCTCCTGGAGTTCACGTT

GGGCGAGTCCGTGGATAAGGAGTGCGTCAAGGCCGATTGTTGCGGCGACGGCTTGCTA

TTTGTCACCGTGCCTTTTGTCGATAAGGTCGTTAAAATCGTCGAGATTGGTGCGTGAAT

GGGAGGGGGACGGGGTGGTCTCACGAAGCTCTGAGAACTCTCAGGAAGGTTTGAGG

GCTGGGACACCGTTTTGCATGCGGTTCTGACGAGTTCGTCGTTCGTAGTGGCCTGGAA

ACTTTGTCCATAAGTACCTTGTCAGTTATTTAGAGTCGTTGCTTTTCTTCACACAACTC

TCTTGAAGGAGTCCAAGCACGCCTGTGTGCACCTGTTTAATGTTCTTCTGTGCTTACGT

TCCCGCAGCAACATTGCATCAAGAAAGAGGCCAAAGATTTGGTTTGGCTTTTGTGGTG

GAAGCAGAAATTCTAATTCCTCCTGGTCTGATGATGTATACCGAAATGTAAAAGAAAGC

ATCAGCACGTTTGCTGCGGAAAAGATAAATTGCAGACTACTCGATATGAGCGCGGCATT

GATCCGAGAGGGCATGTCTCTAGAAAAAACCGGAAGGCTCCACCCGCCTCGACAGTTT

CCGCCAACAGACAGACTAGAAAAAAACACTGATCAAAAAAATAAAGATATGCAGTCT

ATTTTACTGTGCATGACGTCGGGAATCTTCGAGATCTTTCGCATCCGCATTTCCATGATG

GGAGGGGTCACACCACATATCACACTAGACTACATAAACAACCCCTTCCCATGCAGTC

CGCTCCCGTGCTCTGTCGATCATCAAAACAATCTCTACCTTTACAACAAACCAACTAAC

AAGCGAACTGCTTCACGTCAACGGTTCCAAGTTTCACACACAAGCC

>*ChcHsp20-18.7a*

NNNNNNNNNNNNNNNNNNNNNNNNNNNNNNNNNNNNNNNNNNNNNNNNNNNNNNNNNNNN

NNNNNNNNNNNNNNNNNNNNNNNNNNNNNNNNNNNNNNNNNNNNNNNNNNNNNNNNNNNN

NNNNNNNNNNNNNNNNNNNNNNNNNNNNNNNNNNNNNNNNNNNNNNNNNNNNNNNNNNNN

NNNNNNNNNNNNNNNNNNNNNNNNNNNNNNNNNNNNNNNNNNNNNNNNNNNNNNNNNNNN

NNNNNNNNNNNNNNNNNNNNNNNNNNNNNNCGTAAACAAAAATCTTCTCCTCCTTGGCCT

TTTCCCTCCCCTACGCAAGCAGCCCCATTCAACTGCCAACACTAACCATTAATGGTTAC

GCGCGATGCAGTTTGGTTTTGCACATACGAAACATAATCTGTTGTCCGGTTGGCGGCGC

AGGAGGGGCGGGGACGAAGCACAAGGCACAAGGCAGCAAGTAATTCCCATGAAAGC

GTCAAGATGGAAGTCAAGAGGAGAGAGGGAAAGAATTGGGAAAGGTTTGGAAATTTG

TGTGATTGTGAAATTGGTCGGAAAAGTTTTCAATTTCGCAATCACACAAAGTTTTAAAA

GTTTTTTAAAAAAAATTGATCGGAGAAAGTGGGGCTTTGTAAGCGGGTTTGTAAGCGG

GTCTGTAATGGTCAGTCAATATGGAAGATAAGAAGGGCGAGAAAAAGATATGGAAGAG

TTTGCAAGGCGGCGAGCAGGCTGGCGGTTGCGGAGAAAGCGCGACTGGCAGCGCCG

CTACTCGGGGTCGCCAACGCCTCGCGCACAGAACGAGTTGCAGCGAAGCGGCGGTGC

GCTTTTGCACGTCAAGCGGGCGGGGGCCACGTCCCATGAGTGTTTTCCATGAGGACGT

TTCCAAGCCGTGTCCGGGCGGGCGCGGCGAGCCTGCAGCTCGACCTCCGTGCTGTCG

AGCGCCTGTGTCGCGGGCGCAGCGGCGCGGCGATCACTCTGGATGCCTCTGGATGCCT

CTCGATGCCTCCCTCTCGATGCCTCTCGATGCCTCTCGATGCCTCTCCGCCACGCGTCT

CTAGAAAAAAAGTCTACGTGTACATGCACACGCGCCAAAAACACGCAAAAAACGCTC

CCCCCCACCTTTCAACACCCCCCAAAAAAACACCCCCCCTCACCGTCGCCCACCCCCG

CGCGGGGAGCATGCCTTCTCCACCGTCGGACACATACCTCGTTCCTTCGACTTGGCCTG

CCCGCGCGACACGCCAAGCGCACGTCTAGACTTTTCGCGCAGCGTCCGTCCCGCCCCC

CCCCCCCCCTCCTTCTAGAACCGCGACACGGGGTGCTACAGTAGTCCGCCCTGGGAGG

CGAGGCGCCCCCGCCAGCACAGACTCTATATAAACGCACCATGCACCGATCACCCCCT

CTCCCCCTCACCCGCACCCACACCCACCACACCCTTCGGACACACTCCACGACAAGTA

CCCGACCCGCGCCTACACCACACCCCAACTCCTCACCCCCCCAACTTCCTCACCGCCC

AACGCCCGCGCC

>*ChcHsp20-17.9*

CTCAGGCAAGTTTGGTTCTGAGGACATCGGTCATTCCTGTGAGGGGCGCTGAGCAGCC

TGCTTGCTCCCATTTTGTCGCCCATTGCTCACATTCCAAGCTATCGTAACCACATGGTTA

CCTGAAGAAGCGATAGTGTGTGCCGTGTAGTTAATGAACCATTCGTTATCCATACTTCA

AAGAAAAATCGTAATGATGCCCCAATTTTCATAGTCTAGAGCCATGTTGCCGAACGGGG

TGGCCCTTGAAACTACGAGGTTCGATACTGCAGATACCCCCACAAGCTTATGTTTTCAT

TGGGCTGGGGCGGTTGAGCATAAAGTGATGAAAGTGGACGAGGTGCTGTGTTTGAAG

CACATGACTGTCAGATCAGGACACGCGTGGGCAGGTGTTCCGGAACAAGTGTAGAGC

AAGGGGGGAAGCTGTCGTACAGGGAGGTTGGCTCCAGCTTGGAACGGAGATGGTATT

GCGTGTGAGGCAGATAGACATGGAAGTGAGGGGAGGGAAGGAAATATGTGGTGGAAT

GGGGGTTCGTGGAATACCCTGATACGAGTCACTTTTGGGGAGAGCCGTGCTTGGGCAT

TGGATGATGTCACATGGAGGCGGAAGCAGAACATGCAGACGCATAAATTAGGGAATGG

GATGGCGCGCGGCCAAATGTCAGCGTTTCAGCGTAGGCAATGGGAGCAAGTCGTCGA

GATAAAGTTTGGTAAAGTACGGTCGTTTCGGGAGCGTGTTTTATTGAGGACCCTGGAAT

GTGTTTGCCGTTTTTTAATCCAGACGTATGGTCTCTTGGGGTATTCAAATCTGGATACTT

CTCACTCTAGTCGCTGTCAAAATCGGAAGCGGCGGGAGTAAATATAGCGAGATGTGGA

GATTTCCCAACGCAATGTAGTGTTTCTGGGCTGTGCCTTCGCATCTCAGCGGACGTCTA

CGGCGTTCCAGGCCAAGCATTAGGAAACAGGAGTCTGGAATCTCCATCGTTGAGGTTT

CTAGAAAAATCTTCTGGCCAATGAAACAACGCCATCATCCGCCACTTAACGCGGCCAC

AACCAATCACTGTGAGCCCATTTTGCAAAACGCTCCTTTTCTACGGATAAATGCGCGTA

GGAGCATCGCTTTAGCTAGAGGTAGAAGAGCACAAATGGAGTCATCCAAAAAACTAAT

CCTTCGGAGAAGCGCTGTAGATACTCCACATCTCGCTCCTGGAAGGAAAATGAGGAAT

TCAACAAACGCTCTTCATGGAAGTGTGTCTACACGTCAGGACCTCTGAAGCCATAAAT

P-type/G-type

CCTTTGCCCTCCACACCATCCACACTCTTCTAGAATATTCACGAAACGAACCATCTACC

GAAAAGACAAAACCTACAGCAACTCGACCCCGTTCTGCCTCTCGCAAAAGTCTATAAA

GCCCTGCGTCCGAACTTGACTCCTCGCATCATCCCACAAAACAATCCTCACCCTCCACC

ACAACCTACGACCATCTCAAACGCCTTCACCCCCACCCACAAG

>*ChcHsp20-18.7b*

GTACACAATGGGTGGTCGGGACCGGAATAACAGCCTCGTGATGTGGGCACCCTACAGT

GATAATCTAGCACCACCACCGGCGTTCAAGAATGAAGTGACTATGTTTAACAGTCCCAC

GTGGTGCAATACTGTAGAGAGGGATCATGTGTTCGGCGATGATCCGTATTCCTTTATGG

ACGGAGGAATGAAACGGCAGTTCATTTGGCGTACATCGCCAGCAAATCTGCACACTAG

GTACGACCGTCGATCTGTCACTGCGGTCGGTCTCAGTACTACGAACAACGATAATCCAT

TTTTCAGGTAAAATTTTCAATGAGCGGGCGGACACGGTGATTTTCTTTAACACACAGG

GGGGCTAATCCCCTAATCCCGTAAACCCCTAATCCCCCCAAACCCCCCTACTGGCAACA

TTTAGTCTACTGGCAACATTTAGCCTTATTATTATCACTCTAGTGCAGTATGAGAAAATTT

CTGACTTTTACAGTATTTGATGACGTATTTCATGTTTTACGACAAAACGTCGCAGACGT

GTACAGGTACTGTACTGCATAGTCGTTCACTGTAATGACAGTTGCAATGAACAGCATAC

AGCGGTGCCCTGAAATGTTAGTATCTCCGTAGGGGCAGGGCAATACGTAGTGATCTGTG

GTTGGAGGAGAGGCATGGCGTGTGTTGTACCGAACAACCAACTGGCGCAATCTTCGTA

ATCTCGTAGGGCGTGCTCCGTCGCCACTGGTACAAGCAAGGTGCAAGGAGCCGAGCA

GGCTGGCGGTTGTGGAGAAAGCGAAGAGGCGTGCGAAGCGCGCCACCGTGCACCAC

GCTGCGCGGTGGTGCGCCTTACACGGCAAGCGGGCGGGCCATGTCCCATGAGGGCGC

S-type

GGCGTCCAATCGGGGCGTTTCCGGGCGTTTCCGGGCGTTTCCGGGCGTTTCCGGGCGA

GCGGGCGAGCCTGGCCGCTTGACCTCCGTGCTGTCGAGCGCCTGTGCCGCGGGCGCA

GGGGCGCGGCGATCACTCTGGATGCCTCGCGATGCCTCTCCCTGTCTCTAGAAAAGTC

TACGTGTACGTGCACACGCGCCAAAAAACAACAAAAAAATACCCCCGCACCTTCAAC

AACAAAAAAAACACCCCCTCATTGTCGCGCAGCCGCGCGGGGAGCATGCCTTCTCCA

CCGTCGGACACACACACCTGGATCCTTCAACTTGGCCTGCCCGCGCGACACGCCAAGC

ACATGTCTAGACTTTTCGCGCCGCGTCCATCCCGTTTCCGTTCTGGAACCGCGACACGG

GGTGCTACAGCAGTCAGCCCTGAGAGGCGAGGCGCCCCGCCAGCACAGACTCTATATA

AACGCACCATGCACCGATCACCCCCTCTCTCCCTCACCTCACCCACATCCACCACACCC

TCCGGACACACTCCACGACAAGTACCCACCCGCGCCTACACAATACTACAACTCCGCA

CCCCAACTCCTCACCCCAGCTCCTCACACCCCCGACGCCCGCGCC

>*GrcHsp20-16.7*

AAGGTATGTGATAATGGGATTTGCATCTACATTATCAAACGAACGACCTGGGAACTGTC

TGATAAACCCTTGATCGTCCTGAAAGCATCCAAAGTCTCGAATAATCGGAACTCTTTCG

GCCATCAAACGTACAGCTGCGATGAAACGATCTCGACTGGGGCCAGAGCCATCGACGA

AGAATAACTCACGCCGCTTGTTCTCTTCGGCCTTGCAATACGCTATGACGTCCTCACTA

GTAGACACATTCACCAAGGGAGGCGAAAAGGCCGGGAAATATGCAATGTACGGTGGG

G-type

GTGGGTGGTTTCTGGAAGTGCATCACATACTTCTCGAAACAACCTGAAGTACGAAGTG

TGGGTTGCAAAGGCTGGTAAAGGAATGCCTTGCGGTGAACCTTAAGGGTTTCGGTGGA

GGCCGGGCGATAACAGTCGTCCAACCAACTGAATACCTTTCCTATAGAGGTTTCTGGG

ATTGGGGCCGGGAGAGTGCCATTGCATCGCGGTGGGGTTATTGTAGCATCAACAGGAT

CAATGAAGATTACGGGGAATAGACACAACAAAAGAAACGGGGTTGCTGCAATCATGCT

TGGGTTTAACTGTGCTTGGTCGTCACGAAGAGTTGTGGGGAGCGAATTACGAAGCGAA

ACGGGGATGGTTGTTCTTCACGGTGCACTCGCTTTGCGAACGAGCAGTGGGTTTTAAG

GCTGAGAAGATCAGGAAGGAAGCAAGATACGTGCTTCTCTCGCCGAAGCTGCGTCGC

GATCTTTTGCACTGTACCCTTGTAACGCCTCCTCGAAGTTCCAACTGAAAGGGTAGTCT

AGCTAGGCCAGCCATCATGGCATGATGTTGTAAGCCTACTCACATTTCTCAATTTTGGTG

TCGCGTTGACAACAAACTTGAAGTTGCGGATTCTGTCAGCTGCAGTGCTTTTGGTGCC

CATCCCGGCTGTAACCTTTAACCCTGACATTAGTTGACGAATAGGTGTTGTGGAAGAAG

TCCGCAGCTATCGCATTGTGATAGACGCAGGAGGCAAACCTGCTCGAACGAGTTCTTC

CAGGTGATAACTTTGACCAAGGTCTGTGAGGAATCCAGCAATGTCAGAACTTCTGACT

TTGAAGAGTCACGAGACGAGCTTCATTTTCTCTGCTATCTGCATATACGTGTACAGATT

GTTTTTGGACCAGAGGGAGCCGGAATCGGTTATTTCGGTCACTATGCGGGTTTGGGTAT

CTATTGTGTTGCTGTGCAACGACTGCTTCGAGCTGGCATAGGGAAGGTTCCATGCGTAA

CTTTCACTGTACGATGGAGTTTTGACACCTGACAAAGCTGTGTCAAGAATAGGGTAAG

GGTTAGCATCAAACGTTTCCTACCCGGACCCAACGAGCCGGTTGAATTACCTCCACGT

CCGTCAGAACTGGTGCTCACCGTATAGAAACATTGTCACATTTCCCGTACATCGGCAGC

AAGGATAATAGAGCAAATTGTAAGAGCCGACCAAGCCAGCG

>*GrcHsp20-19.0*

AACACCAGGAGAGCTATCCTACAGATAGTTCTGCCTCCTGAGAAAGACCAAAGACTTT

AACAGCCTTCTTTTAATCGACCCTAACAAGGATTGATCCTTTCTTTCTCGCGATGTAGGT

AACTCATCTGGCCTTGTATCTTGTTATATCGTTTATGGGCGATAGTTGTATATCTTCTTGA

AATTGCTGCGTCATTGTAGTAGAAATACATCTGAATACGCTTGGTATCATCAGAATTTAG

ACGGTGATAAGAACCAAGCCATTGTCTAGATTCCCAACCATGTTCCTTTGATGACTCTA

TGATCCACGTACCATTTCCACAGATGGGAGGATAGCCTTCTACGATAATCTACTCTTCGT

CGCTCGGGTTGTCTTACTTGCTGCCGCGTTTGGGTTGTTACTAGAGGGTAAATGCATCT

GAATACTGAATGCGTCGTATGAAACTGAGCGAACGCAAATAACAGCTTTGAAAGATCA

ACACGAAGGCGCCTAAGATGTTTTGACGAAATATGGTCACAATGAACGACACAGCTGT

ATACACATATGTGGCATACAAGGATATGATCGCGTAGGGAGTTGTCTTCAGCCCGGACT

TTATCCTTGAGGGTTAGCGATTGTTAGCAGTTAACAAGGTCAACGCTACCCTTCCTTCG

GTACACTTTAGCGTCGGTAGGGATACCCTTCCCTTTGGCTTAATTTGCGGGTATGGAGTA

ATTTGAACGCTTAATTACCCAATCACATTGGATATTATTGCTGAGTAGGATTCTTGTCAAT

TGGGGGATGTTGCAAGAGCGCAAGATTTTGCACTTTTCCAAATTACACCAGTTTGGATC

TGTACACGTTGCTGAAAGTACCTCCAAAACACCGAATTATTTAGTTTGTTTGAGAAAAA

TCAAAAACCGGCCCAAATCAAGCCAGAGGGAAGGGTAACCCTGTACCAAGAGTGCAA

GAAATAAACCCAACAAAAGATGCAAGATAAAGACGGAGGAGTGACCGCATCCATAGT

CACGCGTGAAGCTTCCCCGTGACGTGACTGTAACGTATGGGCAGCAACGTGATGACAT

GGTTGTGCGCTGCACAACGTGCGGTGGAGTTACAGGCATCCAGTACAGTGCGTACCTT

CGTAATTATAGCAACGATATTCGTGAAGAAATCATTCACCAAGAGTAGATAATTCTACCA

AGGTTAAATGGTCAAGAGTCTAGAACTTTTGAGATCATTCTCCAAGAAGCCGCCAAAG

TTCCATATGCTCTTCTAGTGCAGTTCTAGGTAGAGTTACTTTCAGATTTCTCCAGTTCTT

TCTAGTCCTGTGCACTTCTCACGTACAGCCACAGGCAAGGAGAAACGATGCGCACCAA

TCTCTTTCCTTCAAGTCTATAAAAGTCCAACCCCCATTCCGTTCTCTGCAGACCTACTTC

GACCAACTCAAAAACATAACTCCACTTCCACACAGTACACATCTCTCAAAACGATCAA

TTGCTCATCCATACGTAACTCATCAA

>*GrcHsp20-17.4*

GAGATCGTATGCATCAATGGCCCGAGAAGAAAAAAACAATATCCTGATGTACTGTTCGT

TGTCTGGCTATTACGCGCTGCTTCTAAACTGTTGTTCTGTTTCCACAAACTATTCTTTCA

TGGTGTCTGGTTCTTCTCCAATCTGGAGTTCAGTTGTACTGCCTGGCTATGATAAGAAA

AGATGTCTTTAGAATATTTTATAGCTCTCTTGTGGTATGAAAACAAGTCGTGCACTTTCT

GCGGATGGCGTGTTCGGGATGGAAATTTGTTTTATCTTGACAGCGCAGAACTCCAAGG

TTTTCTTATCAATTTTCTTTAACGTAACACATGCGATTGTCAATTCACACCGTCGGCAGA

ATGCAGCGCAATGTTGATCAACCTTATTGAAAGAAATCGATAGGGTCGTCTACAAAAGT

TAGATTTTCAGTATGCGACACATGTAACATAGCAACGGCAGCGCAGGCTTCGGTAAAA

CTCTATGTTAGAACGAGCAAGCATGAAACAGGTGCTGATCTAGAGAGTATATTCCAAAG

CATTGAAGACTAACAGTATATGCTCCAGTACAATTATGACTTATATCGACCAACGAGTGC

AGATTGCGGGGCAAGAGAAGGGACAAAATTGGTCCTGAGCCCCCAGACGACGTACCA

ATTCTCGCTTCGAGTGACCGTTAATTGTACGATGTTTTCGATATAACACGCTTATCGCAC

TGTTGTCCCGAGTATAGCCAATCAGATGCAAGACACGAATGCATGAGAAATGAACAAG

AAAAAAGATGTCGAAATCAAAAGAGCGGTATCAACAACAACACTGAGATACTATGGAC

CGTTCAGTTATCCTCACTGCTTCGGTGCGATAAGAAAAGAGAGAACAGATCAAGAGGC

ACAAACCAGTCTGCGCGTCCCTGTGATCGCTATCCTCATTCGTCATAAAAAGCAAAAC

GAAGCGACGATTGGATCTCATGAAGATCTTCTTTTCGATTGGGTAATGTGAAATTTCATC

ATGCGTCACGCTGCCACGTGACCGTGCGATGACGTGGCACATCCTTTCCCTTTGCTGG

AATGCGCCCGTTAACTTCCAGAATTCTAGATCATTCTCGACTTCCCTAGCGCCCCGTGAT

GTGCCAACTCCACACACGTAACTTCACTCAATGCATCTTTCAAATAGATGAATGCTTCA

ATGACTATCTTCACATTTCTACAAGTGTTCCGAATGAATAATCAGGGAGCGCTAGAACA

CCACTAGAACAATCTAGGTTCGCCATGGTCAATTCTACAGCCCGTATGCAGCTACAAAC

TAAATAGCATCCCGGCATTAACAAACTTCATAAATTCTATAAAAGCCCGCCCTTCCTCCA

CCTTATATGTCCTCCCTCCTCTCAACTCATCATCACAACTCCCAGTAGTCCATTTCGTCA

ATATCACATAGCTCTTTGGCGCTTTCATACACAAGTACTTAAATGGATCTCTTGTTCAAC

CTCTACGACCCATTTTTTCCGTTC

>*GrcHsp20-19.1*

TCAGAATTTACCTTTTTGAAAGCACTATGTAAACAATTGACTGTTTTTTCTTTTTATCATA

TTCTTCAGGAGACAGTTTCGGTTCAAGTATCTGCATGACTACCTGAAAGCTTTTTCTGA

AAGCTTTTCTTGTGCTCCGGGTTTTAATTTATTCATATATTCCAAGAGAGCAAAGAGTGA

GGCTGTCTTCTCTCGATTCTTTTTATTTCTCGCAAATAGCTTCTCTGGTTGCAACCGTGG

GGCTATTTAGTTTGTTAAGCACAGTCCTATGTTTCAGGATGGAGAATAAACAACATTTC

AGTATATTCTGAGCAGAAGACCAGTGTTCTTTTGTTTAATGCGAACAGAAACTACGACG

TTTATGACGTTCTTAGGAACCGCTCTGGTTCGTTCGTATGTACGCACTGGGATGGGAAC

ACAAAAAACTCCAATCGCCCACAAACAGGTTGTGAAACAGTTAGGGGATTTTATAGGG

ATGCCCCGGCAGCACGAATAAGACGTATTTTAGAGGAAATTGGGAGCGGGGCGACGCT

GTGTTCGCGGAAGGGGGCTGGGCAGATGATCAACAACGGTCGATTGGTTTAGAATAAG

GGTAGAAATTAGCCAAGCAGCTTCTTTGATTTGGGGCTTGGTTAGATTGGAATCGTTCT

ACCGCGGGTAAAGTTACCTTCGTATCCTAGAGCGTTGGTGTGATACAGTTGTCGATTAC

GTACTCTTTCCGTAGTTCCAAAAGCCGGAACACGTATATTGTCAGCTAAACACGTACAA

TAACGAAGGACCAGAGTCCGGGACGTGAAGCCCCAAGTAACCCAAAGGTCGCGTAGG

TAACCTTATATAAACCCACCAATCTACCGGAGCTAAGAGAAGCTCCCGGCAGACTTGTG

CCGCATTTGTTGTTTAAGCTTAGCTGAACGTTCTCACACTGCGTTTACGACAATGAACA

TCGACCACATGTCAGAAGTTCAGGCAACAACAGTAGTTGCAGGAACAACCGCATTCG

AGATAGAGCGCATTTTCCGTGGCACAGCTCAGAAAAAGCAAAAATGAAATGTCACGTT

TGCAGAGTGCGTGACCGCAAAATTGAGACACAGAGAGTCACGTGATTGGCCGACTAG

AAAAATCTCGAAAAATCTAGAATGTTAGATGAGCACAAGTTTTCTTCAAGTCCAACGT

TTCTTTGTATCCACTTCGTCACACCTTCCGCTGTCTCTCGGGAACTCTTGAGAAACATC

TTCCAAGCTGCGTTTATCGCTTGCTATGTTCCTCCAGTACACTTCTACATACTTCCAGAC

GTCTCCATTTAATGCCAATCACAGGAAAAATGAAACGTACCAACGCATCACCCAAACA

TACCTCACGCCGTGAAGTCTATAAAAGGCCATGCTCTCTCTTCTCCCCCTCACCCCACC

CCATCACTCCACCAACTCAACTCGACTAACTGCTTCACACTGCTACAAGTTTCCATAAC

GCTTTACCTGTTCTCACGCATACAATCCTCAA

>*GrcHsp20-18.5b*

AATCTTTCAAGATGAGAAACAGAGCGCCAGTTTCGCAGTCTTTTGCTCAGTCGTTTTAC

TTGCATGTGCGTGATTGGCGGAAACATGACTTTCACGGCATTTCCACAGTTTATCTGCT

GTTCAGGCGCAGGTCCTCTTATCAAAGGAGTATCTTCTAATACAAAACATAGGCAATGC

AGCACACTGAACTAGCACAGAGCTTTAGTAATAGCTCAACCGTGTTACAGTTATGGTTG

GATATTTGTGTGCTGCAAATCAGCACGCCTCATATGAACTATGCACCCAGACGAAAGAG

CATTTGAACGTTTGCTGGTCGGTTGAAAAGGTGCTAACCAGCGATCTTCCTCTCTATTG

TGGTCAACAGCTTTCCTTTACTAAGCTGCAGGAGAGCCTTCAGTAAGAGCTTTCTCTCT

TCAGGAAGGCCAAGAACTTCTGTGGCTCTAACCGGCGATGCTTGCTTATTTGTAACGG

GGATGAAAGATGATGTCATACCTTGCTTGCTACTGGATTGTTCCACATTTCAAGGAATG
AAGACGTTTACCATGCGGCGCGTATGGTTATGTCAATAGATCGCCCGAAGTGTGACTGC
ATTGTAACGCTGAAACTGGAGAAGCGGTACGATGGAAGGTCTAGTTGGTTCTGAGGAA
AGAACGCCAGACTTCATCTTATTTTATAGAGTCTAGTGCCTACGATCGATACATCTAGAA
GATCCTAACACTACAGTACGGGACACTCACGTCGGAGACAGAGCTTGTAGTCTGTCTT
CGACTGAGCTCCGTGGCCCCACAGGTACGTCTGCGTTACATGCAGCCTATCTGTTACTT
CATTTTATGAAAGCCAATATTGCGTCAAGGCAAACCTAGGCCGTCTCAAATCCCCGCTT
CGGTCTTTAAGTTATGACGCAGGCGCCAATATGTGTATCATCTGCGGGCCGTCATCGAG
AAACTCTCAGCGCCGGGGTGCACCCTCGGGGTGACTTCTGACCGCATTCGAAATGAAG
CAACTTGTACAATTTGCCTCCGGCACCTCAGATTAAGCAAAAAATCAAACGTCACGCT
TGAGCAATGCGTGACCATACACACTGGAGACTGCGGAAAGTGACGGGATTGGCTGGG
AACATCTCGAAAAATCCAGAATGCCACACCAGGACAACTCCTCGTCGCGTCCAGTCCA
ACCACCTTCTCCGCACATTCTTCTCGCCACTATCCTCCGGGAACTCTCGAGAAACATCT
TCGAGCTCCCTTCACGATCGCTATACTCCTCATGCACACTTCTACACACTTCCAGATGTC
CCCAATTCCATCCAAATCACATGAAGAATGATCGGTACTGAGATATCACCCAACCATAC
CTCAAACCGTGAAGTCTATAAAAGGCCATGCTCACTCGGCCTCTTCTCTCGCCCTTCCT
CTCTGCACCCAACGACAAAAATCTAACGTCAGCTTCACACAGCATCAACCGTGCATAA
CGTTTCACCCGCTCTTCTACATTCGATCTCAA

>*GrcHsp20-18.5a*

AATCTTTCAAGATGAGAAACAGAGCGCCAGTTTCGCAGTCTTTTGCTCAGTCGTTTTAC
TTGCATGTGCGTGATTGGCCGAAACATGACTTTCACGGCATTTCCACAGTTTATCTGCT
GTTCAGGCGCAGGTCCTCCTATCAAAGGAGTATCTTCTAATACAAAACATAGGCAATGC
AGCACACTGAACTAGCACAGAGCTTTAGTAATAGCTCAACCGTGTTACAGTTATGGTTG
GATATTTGTGTGCTGCAAATCAGCACGCCTCATATGAACTATGCACCCAGACGAAAGAG
CATTTGAACGTTTGCTGGTCGGTTAAAAAGGTGCTAACCAGCGATCTTCCTCTCTATTG
TGGTCAACAGCTTTCCTTTACTAAGCTGCAGGAGAGCCTTCAGTAAGAGCTTTCTCTCT
TCAGGAAGGCCAAGAACTTCTGTGGCTCTAACCGGCGATGCTTGCTTATTTGTAACGG
GGATGAAAGATGATGTCATGCCTTGCTTGCTACTGGATTGTTCCACATTTCAAGGAATG
AAGACGTTTACCATGCGGCGCGTATGGTTATGTCAATAGATCGCCCGAAGTGTGACTGC
ATTGTAACGCTGAAACTGGAGAAGCGGTACGATGGAAGGTCTAGTTGGTTCTGAGGAA

AGAACGCCAGACTTCATCTTATTTTATAGAGTCTAGTGCCTACGATCGATACATCTAGAA

GATCCTAACACTACAGTACGGGACACTCACGTCGGAGACAGAGCTTGTAGTCTGTCTT

CGACTGAGCTCCGTGGCCCCACAGGTACGTCTGCGTTACATGCAGCCTATCTGTTACTT

CATTTTATGAAAGCCAATATTGCGTCAAGGCAAACCTAGTCCGTCTCAAATCCCCGCTT

CGGTCTTTAAGTTATGACGCAGGCGCCAATATGTGTATCATCTGCGGGCCGTCATCGAG

AAACTCTCAGCGCCGGGGTGCACCCTCGGAGTGACTTCTGACCGCATTCGAAATGAAG

CAACTTGTACAATTTGCCTCCGGCACCTCAGATTAAGCAAAAAATCAAACGTCACGCT

TGAGCAATGCGTGACCATACACACTGGAGACTGCGGAAAGTGACGGGATTGGCTGGG

AACATCTCGAAAAATCCAGAATGCCACACCAGGACAACTCCTCGTCGCGTCCAGTCCA

ACCACCTTCTCCGCACATTCTTCTCGCCACTATCCTCCGGGAACTCTCGAGAAACATCT

TCGAGCTCCCTTCACGATCGCTATACTCCTCATGCACACTTCTACACACTTCCAGATGTC

CCCAATTCCATCCAAATCACATGAAGAATGATCGGTACTGAGATATCACCCAACCATAC

CTCAAACCGTGAAGTCTATAAAAGGCCATGCTCACTCGGCCTCTTCTCTCGCCCTCCCT

CTCTGCACCCAACGACAAAAATCTAACGTCAGCTTCACACAGCATCAACCGTGCATAA

CGTTTCACCCGCTCTTCTACATTCGATCTCAA

>*PrpHsp20-25.2b*

AAACAGCGGTTTTCGTAGACGAATCCGCCGCATGCGAGAACTTGGCCGGGCGAGCTG

CAAAGTTGCGATCCCACTGGGTTTCAACATGAATCTGGACTGTGGGTCTCTTACACGC

GCTTGACAACCAAGCCCTCCCAAGCGGAACCTTCTCCGCATCATCTGCACCACTGAAG

AGCATTTTCCACATATACAAAAGGATAACAGGTACACAACTTTATGTTTTTTTCTTTCTC

TACAAGCTAAGGACACAGATCAAGACGCTGGCATCGACCTCACTGATCACAAAGAGC

CGCGTACCAGCCTAATCGGACTATGGCATCTCCAGCAGGACTCGCGCAACGCCAAAGG

ACTGTTGACAAACGTGGTCAGGATAATGTCCATACGATGCACTTCTGTCACCAATTCTT

CCTGGCGCTCTCTGCCTACTTAATTTCAATCAGGCGAGTGGCAGGCTCTTCGCGGCCGT

GCTTCGGGAGTTCTATCGTAACAATTCCGTCCTTGGCTGACGCCGAAATCTTGTCAATG

TCAACGTCATTACCAAGCGTCAGAGAACGATGGAAGGAACGCGCCACGTAGCCATGC

S-type

GTAACTTTCTCGCCCTTTCCGTCGCCATTCTCGCCATCTTCCGTGACGTTCTCTTCCATT

CCGCCGCTGACCTTCAACACATTGTCCTTCACTTCCAGCTGAATTTTCTCCTTCGGAAT

GCCCGGCGCCTCAATGGTCATCACATACTTGTCGTCAAACTCACTGAGAAGGCTTGGG

GGACTTGCGCGCACAGGCTCCAGAAGACTGTTCCAGTCGTCGCGTGTCGTTCCAAAA

GGCGCGCCCAGCAAACGGTCAATCAGTTCAAAGGTGTTTGCGCGACCCGTCGGGTAC

GCCAAATGCGAAGAAACTGCTGGACGCGTCCTCAACGCCATTGGTGAGCTTGCACGCT

TCGCCACACTCTTCGCAGCTTTTTCCTTGTTCCTCTTTGTCGACTTCGTCGCTAATCTCT

TCACTACACTTGGTCGAATGTGGTACACTCTCATCCTGACGAAGATGGCCACCCTCCAT

CGCGCGCCGGCTTCGACATGCACGTGCCGGCGCCCCTTTTTATGCTGTATTTCATCAAG

ACATTTGTAAGACAGGTGCACGTGTCTCTAGACCTTTCGCAAATCTTCTCGTCTCGCTC

CGACACAACGTTCGTTGCCGGCGTACGCAAAAAGCAGGCTTCCTTCATGAAAGTGTGT

ACACTTCCACATGTTTCCACAAAAATCTGGAATGGGCACGGAAGGCTCCGGCTTCTCG

CATCGAATGACGATGCCTTGCTTGCTCCGGCGTGGCATGTCGTGGCATGGCGTGCTCGC

ACGGTTATAAAAGCCGGCGCCTGTCCACGCAGTTACCCGCTCCGGCGCGGAGTATCTG

CAAGGCGAAGGTGAGTGGTGTGGAGAACAAAGTGTCCAGGGAACAAGGAGTCAGAG

CATTTGCTTGCATTGATTGAGACTACCAGTGAAGCACACAATC

>*PrpHsp20-22.0*

ACCGTCTCAGCACACTGCGACGGGCGGACCGGATCCTCTTTCTCGCGGATGGAGCCGT

GGCCGAGCAAGGGACGTATGATGAGTTGTCGCAGAGAAGAGGGCTTTTCCGGGGATAT

GTGGAGGCCTCGCAAAAACAGCGATTTCACCCAACAAGTGCCCAATAACTTGCAGTCC

CGTCAACCATTTACAAGCATGCTGAACTATGACTAGACTACGAAAACTAAAAACTAAC

CTACACGTGGACGAGAATTCCAAACCAGCATAGAACACATACAGAGCTTTGGGCTCTC

ACAACGAGGTCAACTTCCAGGTCATTCATCTATCACACAGAGGCATCAATGCGAGGCA

ATTTCAATGCGCTTGGGCTTCGGTTCCTCCGGCTTCGGGCGCTTCGGTAACACGACAG

AAATCACGCCATCTTTTGCAGCTGCCGAAATCTGATCAACGAGGATATCCTCACTCAGC

ACCAGACTCCTCTTGAAGGACCTTTTCGTGTAGGATTCAAAGACAGCACCCTTCTGCC

CGGCCGGTTCACCTCCGTCAGACGGCTTCGTCGTCTTCTCTTCCAAGTTTCCAGAGATC

AGCAACATGCTGTCCTTGACTTCAAGCACCACCTTCTCCTTCGGAATACCCGGTGCTTC

CACGTAGAGGACGTACTTATCATCAAACTCCTCAAACTTATACGTCGGGCTCCAAGCAC

GCTGCTGTGCAGGCGCAAGCCCCATGGCACGGTCCCACTGTTCGAACGGCGTGTTCAT

GAGCCTCTCAGCAGCGTCCATGAGATCAAATGGACTCAACGGTGCAAATGGCGACAAT

GGTGCGAACATTTGCCTGTTCGGACGCGCCATAGCCCGGGATTGCGCTGGCGGCTGGG

TACGTATGGCGACGTTGCCGTTGCCGTTGCCGTCTCCTTCCTTGCGAGGCGCATTCTGC

GGCGAAGTCTGCATGTACGACGGTTTCGCGCCATACACCCGAATCGTTGGGGCCACTC

CAAAAGTGAACGCCATGCCGTCACTCTCCACCATGATTGTGTGCTTCACTGGTAGTCTC
AATCAATGCAAGCAAATGCTCTGACTCCTTGTTCCCTGGACACTTTGTTCTCCACACCA
CTCACCTTCGCCTTGCAGATACTCCGCGCCGGAGCGGGTAACTGCGTGGACAGGCGCC
GGCTTTTATAACCGTGCGAGCACGCCATGCCACGACATGCCACGCCGGAGCAAGCAAG
GCATCGTCATTCGATGCGAGAAGCCGGAGCCTTCCGTGCCCATTCCAGATTTTTGTGGA
AACATGTGGAAGTGTACACACTTTCATGAAGGAAGCCTGCTTTTTGCGTACGCCGGCA
ACGAACGTTGTGTCGGAGCGAGACGAGAAGATTTGCGAAAGGTCTAGAGACACGTGC
ACCTGTCTTACAAATGTCTTGATGAAATACAGCATAAAAAGGGGCGCCGGCACGTGCA
TGTCGAAGCCGGCGCGCGATGGAGGGTGGCCATCTTCGTCAGG

>*PrpHsp20-17.1a*

GAAAGGTCTAGAGACACGTGCACCTGTCTTACAAATGTCTTGATGAAATACAGCATAA
AAAGGGGCGCCGGCACGTGCATGTCGAAGCCGGCGCGCGATGGAGGGTGGCCATCTT
CGTCAGGATGAGAGTGTACCACATTCGACCAAGTGTAGTGAAGAGATTAGCGACGAAG
TCGACAAAGAGGAACAAGGAAAAAGCTGCGAAGAGTGTGGCGAAGCGTGCAAGCTC
ACCAATGGCGTTGAGGACGCGTCCAGCAGTTTCTTCGCATTTGGCGTACCCGACGGGT
CGCGCAAACACCTTTGAACTGATTGACCGTTTGCTGGGCGCGCCTTTTGGAACGACAC
GCGACGACTGGAACAGTCTTCTGGAGCCTGTGCGCGCAAGTCCCCCAAGCCTTCTCA
GTGAGTTTGACGACAAGTATGTGATGACCATTGAGGCGCCGGGCATTCCGAAGGAGAA
AATTCAGCTGGAAGTGAAGGACAATGTGTTGAAGGTCAGCGGCGGAATGGAAGAGAA
CGTCACGGAAGATGGCGAGAATGGCGACGGAAAGGGCGAGAAAGTTACGCATGGCTA
CGTGGCGCGTTCCTTCCATCGTTCTCTGACGCTTGGTAATGACGTTGACATTGACAAGA
TTTCGGCGTCAGCCAAGGACGGAATTGTTACGATAGAACTCCCGAAGCACGGCCGCGA
AGAGCCTGCCACTCGCCTGATTGAAATTAAGTAGGCAGAGAGCGCCAGGAAGAATTG
GTGACAGAAGTGCATCGTATGGACATTATCCTGACCACGTTTGTCAACAGTCCTTTGGC
GTTGCGCGAGTCCTGCTGGAGATGCCATAGTCCGATTAGGCTGGTACGCGGCTCTTTGT
GATCAGTGAGGTCGATGCCAGCGTCTTGATCTGTGTCCTTAGCTTGTAGAGAAAGAAA
AAAACATAAAGTTGTGTACCTGTTATCCTTTTGTATATGTGGAAAATGCTCTTCAGTGGT
GCAGATGATGCGGAGAAGGTTCCGCTTGGGAGGGCTTGGTTGTCAAGCGCGTGTAAG
AGACCCACAGTCCAGATTCATGTTGAAACCCAGTGGGATCGCAACTTTGCAGCTCGCC
CGGCCAAGTTCTCGCATGCGGCGGATTCGTCTACGAAAACCGCTGTTTCACATGGCTA

CAAGTGTACATATCGTGAGCGCACTGAACCGCGTTCTAGCAGCAAGTACAGCGCAGAA

GTACAGTTCTGGCCAGAGAGGCTCGAGAATTTTCCGGATGGCTCCGTGCAAGTGGCTG

P-type

CGGCATGGCGAATCTGCCTCGAGAATTTTCCGGAAGGCTCGGGCGACTGCTTGCGCCT

CTGCTGCTGCGGCCTGAGATGGCGATTCGACACGGGTATAAATTGGTCGCTCTGCACC

AACAAAGGTCGGAGATTGTATGAGTGACAATGGAAAGGTGGAGGAAGAGCCAGCGAA

CAGGTACGGAGCTGAGCGAGAGTAAGCGTTGGAGCAGACAAAAAGTCTGGAAAG

>*PrpHsp20-25.2a*

AAACAGCGGTTTTCGTAGACGAATCCGCCGCATGCGAGAACTTGGCCGGGCGAGCTG

CAAAGTTGCGATCCCACTGGGTTTCAACATGAATCTGGACTGTGGGTCTCTTAAACGC

GCTTGACAACCAAGCCCTCCCAAGCGGAACCTTCTCCGCATCATCTGCACCACTGAAG

AGCATTCTCCACATATACAAAAGGATAACAGGTACACAACTTTATGTTTTTTTCTTTCTC

TACAAGCTAAGGACACAGATCAAGACGCTGGCATCGACCTCACTGATCACAAAGAGC

CGCGTACCAGCCTAATCGGACTATCGCATCTCCAGCAGGACTCGCGCAACGCCAAAGG

ACTGTTGACAAACGTGGTCAGGATAATGTCCATACGATGCACTTCTGTCACCAATTCTT

CCTGGCGCTCTCTGCCTACTTAATTTCAATCAGGCGAGTGGCAGGCTCTTCGCGGCCGT

GCTTCGGGAGTTCTATCGTAACAATTCCGTCCTTGGCTGACGCCGAAATCTTGTCAATG

TCAACGTCATTACCAAGCGTCAGAGAACGATGGAAGGAACGCGCCACGTAGCCATGC

S-type

GTAACTTTCTCGCCCTTTCCGTCGCCATTCTCGCCATCTTCCGTGACGTTCTCTTCCATT

CCGCCGCTGACCTTCAACACATTGTCCTTCACTTCCAGCTGAATTTTCTCCTTCGGAAT

GCCCGGCGCCTCAATGGTCATCACATACTTGTCGTCAAACTCACTGAGAAGGCTTGGG

GGACTTGCGCGCACAGGCTCCAGAAGACTGTTCCAGTCGTCGCGTGTCGTTCCAAAA

GGCGCGCCCAGCAAACGGTCAATCAGTTCAAAGGTGTTTGCGCGACCCGTCGGGTAC

GCCAAATGTGAAGAAACTGCTGGACGCGTCCTCAACGCCATTGGTGAGCTTGCACGCT

TCGCCACACTCTTCGCAGCCCTTTCCTGGTTCCTCTTTGTCGACTTCGTCGCTAGTCTCT

TCACTACACTTGGTCGAATGTGGTACACTCTCATCCTGACGAAGATGGCCACCCTCCAT

CGCGCGCCGGCTTCGACATGCACGTGCCGGCGCCCCTTTTTATGCTGTATTTCATCAAG

ACATTTGTAAGACAGGTGCACGTGTCTCTAGACCTTTCGCAAATCTTCTCGTCTCGCGC

CGACACAACGTTCGTTGCCGGCGTACGCAAAAAGCAGGCTTCCTTCATGAAAGTGTGT

ACACTTCCACATGTTTCCACAAAAATCTGGAATGGGCACGGAAGGCTCCGGCTTCTCG

CATCGAATGACGATGCCTTGCTTGCTCCGGCGTGGCATGTCGTGGCATGGCGTGCTCGC

ACGGTTATAAAAGCCGGCGCCTGTCCACGCAGTTACCCGCTCCGGCGCGGAGTATCTG
CAAGGCGAAGGTGAGTGGTGTGGAGAACAAAGTGTCCAGGGAACAAGGAGTCAGAG
CATTTGCTTGCATTGATTGAGACTACCAGTGAAGCACACAATC

>*PrpHsp20-22.1*

CCGTCTCAGCACACTGCGACGGGCGGACCGTATCCTCTTTCTCGCGGATGGAGCCGTG
GCCGAGCAAGGGACGTATGATGAGTTGTCGCAGAGAAGAGGGCTTTTCCGGGGATATG
TGGAGGCCTCGCAAAAACAGCGATTTCACCAAACAAGTGCCCAATAACTTGCAGTCCC
GTCAACCATTTACAAGCATGCTGAACTATGACTAGACTACGAAAACTAAAAACTAACC
TACACGTGGACGAGAATTCCAAACCAGCATAGAACACATACAGAGCTTTGGGCTCTCA
CAACGAGGTCAACTTGCCAGGTCATTCATCTATCACACAGAGGCATCAATGCGAGGCA
ATTTCAATGCGCTTGGGCTTCGGCTCCTCCGGCTTCGGGCGCTTCGGTAACACGACAG
AAATCACGCCATCTTTTGCAGCTGCCGAAATCTGATCAACGAGGATATCCTCACTCAGC
ACCAGACTCCTCTTGAAGGACCTTTTCGTGTAGGATTCAAAGACAGCACCCTTCTGCC
CGGCCGGTTCACCTCCGTCAGACGGCTTCGTCGTCTTCTCTTCCAAGTTTCCAGAGATC
AGCAACATGCTGTCCTTGACTTCAAGCACCACCTTCTCCTTCGGAATACCCGGTGCTTC
CACGTAGAGGACGTACTTATCATCAAACTCCTCAAACTTATACGTCGGGCTCCACGCAC
GCTGCTGTGCAGGCGCAAGCCCCATGGCACGGTCCCACTGTTCGAACGGCGTGTTCAT
GAGCCTCTCAGCGGCGTCCATGAGATCAAATGGACTCAACGGTGCAAATGGCGACAAT
GGTGCAAACATTTGCCTGTTCGGGCGCGCCATAGCCCGGGATTGCCCTGGCGGCTGGG
TACGTATGGCGACGTTGCCGTTGCTGTTGCCGTCTCCTTCCTTGCGAGGCGCATTCTGC
GGCGAAGTCTGCATGTACGACGGTTTCGCGCCATACACCCGAATCGTTGGGGCCACTC
CAAAAGTGAACGCCATGCCGTCACTCTTCACCATGATTGTGTGCTTCACTGGTAGTCTC
AATCAATGCAAGCAAATGCTCTGACTCCTTGTTCCCTGGACACTTTGTTCTCCACACCA
CTCACCTTCGCCTTGCAGATACTCCGCGCCGGAGCGGGTAACTGCGTGGACAGGCGCC
GGCTTTTATAACCGTGCGAGCACGCCATGCCACGACATGCCACGCCGGAGCAAGCAAG
GCATCGTCATTCGATGCGAGAAGCCGGAGCCTTCCGTGCCCATTCCAGATTTTTGTGGA
AACATGTGGAAGTGTACACACTTTCATGAAGGAAGCCTGCTTTTTGCGTACGCCGGCA
ACGAACGTTGTGTCGGCGCGAGACGAGAAGATTTGCGAAAGGTCTAGAGACACGTGC
ACCTGTCTTACAAATGTCTTGATGAAATACAGCATAAAAAGGGGCGCCGGCACGTGCA
TGTCGAAGCCGGCGCGCGATGGAGGGTGGCCATCTTCGTCAGG

>PrpHsp20-17.1b

GTCTATTCGTCATGAACGCAAAATGCTGCGCGGCGTGCATGAACCAACGCTTCCCGAG

TCCGGCCTGCACAAGATTCGCACGGATTCCCTGATTCAGCGTGTGGTGAAACCGCTCC

TGTTCAGCGTGACTGTTTGGCCTGTACGGAATCCCCACCTCGTGCTTTATGAGGTGTTC

ACGCACCAGTCTCCCCACATCTCCCTTGAACTCATTCCCATCATCATCTCCTGTCCCGC

AATGCGCGCATGCAGCTGATTAGCTGCCTCAATCCATGCCGCATTCTCCTGCGGGACAT

TCATTTCCATTTCATGGCTTTCCATTTTCTCCAAACAACCGGGCGATGATACCACTTGGA

ATTTCTCCCTTTCTGCTTTGTAACCGAATCAACAAAACCGGACCGCTTCACCGATAACA

GGAACTGGAAACCGCCACACCCCGGAAACTGACAAACCGACGACGAACCGACACAC

CGAAACGAACGGAAGGACAAGCGGGACAATCAGAGAAAAGTGGCTAGAAAGGAAGA

GAGGACAGAAAATTCAACTTATAGGGGACTACGAGAGGGAGTGGACCGGTCTTGACA

AGTGAAGGGTGATGGAAGGTACGTTTAAACCCCAGCTGCCTTTTTAAAAACACTCCAG

ACCGATATCCGCTCGCACAACACGCTAGCGTGCCTCTCCCGCAAATCCAACCCAAGTC

TTCCAACGACTGCCTCAATCCAACTAACCAATTCTCTTTTTTATTCTATTTGCCCTTACCT

CTTCTTCTACCTTGACCTGGAAATTCCTGGGAACCAGCTTGCCCCTTCGTCCTTTTATAC

CCTTCGTCCTTGCTCTTTCCAGCAAGGACGGGAGAACAAATTTCTCCAACAGGACGAA

TTTCCACCACAGGTTTTGATTCGTTCCACAACTCGCAACCACAGCCGGCCACGTATCCG

GAAAATGGCCGGGAGGGCGCGTCAAGAACGAATCCAGAAGCTTCGGCCGGCCTTTGA

CCTTTGCACAAGGCCACACAAAACGACAATGACAAACATTTCGCAGGTCCGAGATCAT

CACGCTTCTCGAACAATCTTTCATCATAGAACTTTCTAGGGTTTTGTACTCAAAACCCT

ACGGCGTCTCTCGGGGCGGAACCCTAAAATTCCACGTTCACATGGCTACAAGTGTACA

TATCGTGAGCGCACTGAACCGCGTTCTAGCAGCCAGTACAGCGCAGAAGTACAGTTCT

GGCCAGAGAGGCTCGAGAATTTTCCGGATGGCTCCGTGCAAGTGGCTGCGGCATGGC

P-type

GAATCTGCCTCGAGAATTTTCCGGAAGGCTCGGGCGACTGCTTGCGCCTCTGCTGCTG

CGGCCTGAGATGGCGATTCGACACGGGTATAAATTGGTCGCTCTGCACCAACAAAGGT

CGGAGATTGTATGAGTGGCAGCGGAAGGGTGGAGGAAGAGCCAGCGAACAGGTACG

GAGCTGAGCGAGAGTAGGCGTTGGAGCAGATAAAAAGTCTGGAAAG

>PrpHsp20-17.6

ACAAATCGCATCGATGCCGGCACTTTGACCAGGAGCTTGCCAGTTCCCATCACAAATC

CTGCATCAGTGTTGCCAATGCCCGTGGCAAACATGCCGAACGCGCCGGCGTTACACGT

GTGCGAGTCGGTTCCGAACAAGATCTCGCCCGGGCGCGTGTGCCCCTCTTGGGCCAGG

GCAATGTGGCACACGCCCTTGTAGTCCGGGTTGACCTTGAAATCGCTTCGGTCTACAAT

GTCGTAAAAGTACTTGATGTTCTGCTCCCTGGCAAAATCTCTGAGAATATCGACGTTTC

GGTTTGCGCGCTCGTCGGCGGTGAAAATGTAGTGATCTGGAATGATGACGACCTTCTC

CTTGTCGAACACCTTTGCGTCGGCGCCAAACTCTCGCTTGAAAATGCCAATGGTGCCC

GGCCCGCACACGTCGTGCGTCATCAGCACGTCCACGTCAACCCAGATATTATCGCCAG

GCTTTACGCTGTCCTTGTTTGCGCCGCGCGCAAGCACTTTCTCGACCAGCGTCATTGGG

CGCACCAGCTTGCTCTTCTGCGCGTCCTCCACGCGACGCTCGTCCGAAATCACCATCC

GCACTGCCGCGGGGCGCCGGCCAGCGGCACCTCGCCTGCTCTTCACCTCTGCCGCTCC

GCCATGACGTGCGCGCACAAGTCCAGCTCCAACGGCGCCGCTCGCGCTCCCCACAAA

GCACACGCCCGCCATCGTTCCCTAGTCACACCGCTCTCTCCTCTCTCCCTCTCCTTCGC

CTTCGCCTTCCCCCTCACGCGCCTCACCCGCCGACGTTGAGCCGAAAATCGGCCGCGG

TACAGTGCACACGCAGTTCACTGTACAACGACAGAACACGGAAGTACACTGATGTCAT

CATGATAAGTTAAGAGATGACAGCTTCCCCAAAAACGTCATCCGGTTCACGGAAGGAA

TACACCGCTCGCGCGGAGCTGTACCATATTAAAGGCAATGGGGTACTGTTATAAACGGT

TCATGTGGGCCGAGCGGAATCTGGAAACCACTAGAAACCGCGGGAAACCGGTTTCGA

GCAGGGCTGGATGATGTTGACACCGTTCGACCAAGTGGGTTGGACCTTGATGCGATGT

S-type

GATGCATGGGTCATGGTGGCAGCAGGTCTTCACACTTTTCCACACGTTTCCACAGATTT

CCACAGGATTCTGTAAGTCTTCTAACGTTTCCGAAACATTCTACAGACTCCCAGAGGGT

TCGAAAGCGTCTGGAAAAAGTACGGAATGTCGGAGAAGCCTCTTCGAAGGATCCAGA

ACGTGAACGGGATGTCCGCGCATGTCTGTCACGCGACGGGCAGTGCGGTATAAAATTG

GTCTCCAGGCCTGGTAAACTTCAATGAGCGCAGCGGAGGACGCGAAGATAGAGAGAC

AGAGACAAAGAGAGAGAATCGGAGCAAGGCCAGGGAGACTCAGGAGAGAAGCGTAT

CTGTGAGGACAGAGAGCATCAAGAGACAGGGAAGAGAGAATAGGACAGA

>*PrpHsp20-37.4*

CTCGTTCAGCGAGGACAGGTGAATGGAACTAGCCGGCCCGAGAAACAGCGTCCCTTT

CTGGACAGCAGGGTCTGCGCGCTTCACCGTGCCACGAGAAAGCACCGAGTGTAAGGA

CTGAAGTTCCGGACCGTGCCCAGATACCAGATTCAGGTATTCGCGCGCAAGCTTCGCA

CCGCCGAGCATGGTATCGCGACGAGTCCATTCGCTCATCAGTTCAGAAAGGAACGCGT

CCTTGCGCAGCAACGCGGATTGCTCGAGCCACTCGAGATTCAGCTTGTAGTGTGGCGG

CACCGTGACAATGCGAAAGGTCTCATTCAGCAGACTGTCTGGCGAAGCAAATTCGCCC

AGCAGCTCGCAAAACTTGACGTGCATTAGCGCATGGGCAAGCGGGATTTCGTCCTCGT

CGTTCTGAATGGCAAGCGCAAGCGCCTCTCGGTTTGGTGACCCTCTGCCACTGCCACT

GCTGCTGCTGCTATTACCATCCGCACGCCGTCTGTGTATTGGCGCAGCCCACGTCACGA

GCTGGCGAGCCAGTGCGTGGCGTGATTCTTTGAGGTGACGAACGCTCCGATGGCGCG

GCACATTTTCTAGTTTCCAGCCTTCGTACTTGCTCAATTCTGCGTATGTATTCGATATGTA

CATCATTTTGTGCTTCCATCGCAGCCACCGCTCTGGCGTGGTCGCCAGACTTTCCAGAA

AGTAGCGTGGCTTGTGCGCACCCTTGTGTGTCCGTTCCGACTCGTAAATGACATAAACA

TCGACAAAGTCAGAGGTCTCGAGCAATCGGATCTCGAGCAGGTCCAGGTTGAATCCG

ATTGGCACAAAGTCAACAATGCGGCGCGGGCTTGCTGATTGAGGCAGGCGTGCCCCG

CTCAGTGCCATCGCGTACCTTGCATCCGAGCAGTTCACAGGCAAGCGCGCGCTTGAAC

TCGGTGGCAGGTCCGGATTCGAAAACACTTGCAGGGGCGCTTCGGGTTCAGCTATCAC

GTACGGGGTCACGAGCTCCATGAGCGCCTCGGCTGTCTCTGCGTGATCCAAGTCCCAT

GGTGCCGATGAAAGCGCGCCATCTTCTGTGATGAGCAACTCGATGAATGCGGACGAGT

CAAACGTCCTCTTGAGCGGCGGCGAAACCGAGGCATTGCCGTCGGCCCGAATCATTTC

AATGGCATCCGCAGCTGACACGCCACGCGCAATTCTGGCGCGCGTTTGCGGTGACAGC

GAAGCAAACACACCCAATGGTCGCGCTGGTCCAGCAGCTGGAGCCTGGTCTCGTGCC

GCCTCTGCCGCAGCGGCCACGTCATGATTCTCAGCTAATACCGGAGCGACGGGCGCAA

CGGTCATGCGATGTGCGCATACCAGCAGCGCCACTGCTGCGCACACAGCAAAGCCTGT

GCCCACCCATTTCGCCCACGCGCGTCGCGTCATGTCCGCATCCGCGCGAACGAACACG

CACGTGCGAGCTGAAATGCAACGCATCATAATAAAACACACGTTTTTGTT

>*PyyHsp20-20.3*

GATCGTTTCGCGAGAGGGCCGGGGCGGAGGGGCGAGCGCGCGCAAAAGGTGGACGC

AATTTGACAGCGCCAGGATGCGCATCGTCGCCGTAGCCCCCCCCGTCGGCTGGGCGCG

GGGCGGGGGCTGGGGGAGGGGGGGGGGGGGGCGCACTCGATCACCGATATTCACTG

TGATAAGGCTGCCCCTCCCCTGCCACGCCCCTTGTCCAATGGGCAACGGGGAAGGCCC

GAAGTGATTGGCACGGAAGAGCGGACAGCCCGTAAAACACACCGGTCGGGCCCGTGT

GCGCGTGCAAACCTTCGCAACCGGTGCAGCAGCACATGGCGCGCCGCGGCGCATGCG

GCACAGAGTGCCCCCTGCTTGTAGCGACGCCGTACTTTTACGAGTCGGGGCCACGGCC

CTTGGGCTGTCCATGTGACGGCTGGCCCGCGTCTGGGACCGCCCGGGCCAATGGGCGT

TGGCCATCCACGCCATCGACGCGGGTCGTCTCGGCCTGGCACGGAGCGTGCGTATTGT
GAACCGTACGGGTGTTGTGAAGGACGTGGCGTCGCTGGGATGGTCTGTGGTGCGGCG
GCGGGGGTCCCCCTTCACCAGGAGACGCGGCGATTTTGGGCCTATTGGTGTGGTCTGT
CGCGTAGCACAGGTCCCCACAACGCTGAGCGCGTTGTCGGCTGGATTTATGGGTTGGT
TGGCTGCCAGCTACGTTTGGCGGGCCAGGAGTTGACGCGCCCTTTTCTGCCATTGGGT
CGTGGCCAGGATAGGCGTGGGGAAGTGGGGGGGGGACTGTGCGAAGCTCACAGACCT
GCCACCACTCGGAGCCAACCTCTCCGACCGGATGGCCGTCCGCCGTCGCATACAGTAC
AGGGGGCACTGGGCACTGCTGTACGTCAAACTTCCACCGTGGCAAGCGCTGTTAGAG
CCGATGTCGCGACGGACTGGACGCGTTCAGTGATCTGAACTACTGTTAGCCTCGCAAG
TCTCCTACACAGGACAAATCTGCATTGCGGTGCGACACGCCACTGATATGAGTAATTGA
CAGCGTGCACGAAACTTCGAGAGCACTCTTGGAGGTTCGAGAGGGGCAGTCCGGAAT
GTTCGGGACAATGCAGTCGAATGTTCGAGAGCGTGCCAAAGTGCGCGTGCGTCCAGC
GCACTTCGTTCGCGCTGCCCTGCTCGCCGCGGCTGTCGTCATCGGCTTCCGTCGGCTTG
TCGTGTGCAGCCATGGGGCATGCTGCACATGGAAGGAGGCTCACCGTGATCAGGAGG
GGGCTGCAGCCCTCGCCTACTTAAGCGGTGCCGGCGAGTGTATAGGGCCCGCATCCCA
TCTTCCGCACCACGACCTATCCACCCAACTCCCCTTCTCGCTATCCTTCTCCCGCTCTCT
CCGCCTGCACCATTGTGCACCTTCCCTCTATCTCCTCTGCCGTCCTTGCCTGCTGCGGC
ATCATCCTGTCACGAGGAGCAAGCCTGCTTCTTCCCCGTTTCTCTCCCACGGCGCC

>*PyyHsp20-19.6*

TCCCAAGGGGCCAAATCGGCCCCGTCCTCCTCACTAGGAAGGTGGGCACAGGGGTAC
GACGCGATCTCTTCGAGGTCCCCCACATGGAGGGCCCATAGGGTCCCTACAAGGGGG
ACGGACGCAGGAGTGCGGGCAGGGGTGAGTGCGGGTGAGGTGGGATCGACCGCTGC
CAGCGAGCTCGAGACCGGCGAGATTTGTCGCAAAGACCGACGAGATTTCTCCCATCTT
CGCCTGCAGTGTGTCAAACAGTACGTATTGTACATACATAGTTACTCTTGGAAACGATG
TCAAACAACTTGGAACTGTTTTCAACGTACACTTTCTTTGGAGACAGCGTCATCGTTAA
ATTTCCACCCAAGGGAGACACGGGCAGGGCGACCCCGTGGCGGTCAAAAGCCCCCGC
AACAGCACCCATAACGCGCACTCTCGTGGAGACGCCCTCGCGCTCCCAACGGTCTGAC
GCGGGAGCAAGTCCACGGCTACGCCCAGCAGATAGTGCGCCCAAGGCGATCTAACCAT
GAGCACGAAGCAGGGAAAGAGGGTCGATGAGCGGGGTGCGCTCGGCTCCCCCGCCG
CCGCCTCGTCGTCATGAAGCGGTGCTGCCGGCGCCCTCTTGGCGGACGCCGTGAGTGG

GGGCAGGGTCCGCATAGTGTAAGACCAGACGTCCTGGGATTTTGGCGTCAGCTCGTCA

GGGGAGGTGCACCGGCAAGGTTTTGATCATCAACCATATTGAAGATTCCAAAACTTGT

TTGACACTCTGTTCTTGGGTCTGGTGAAGGAGCGCGAGACCTGCAAGATGTTCTCT

CGTCTTCAAAGACAACCTACCGAAAGTAAATATTTTGTTGCGAGAATGGTAGATATCAT

CCCCATGTAATTTGTGTTGTTACGTGCTTCAACGGCGGCCAACCAACGAGAGTCCACA

CGTTGATCCAGACTGTTCCAGATCCGTGCAAAGCGGCGGCTGTCCAAGACGCTCCGCC

TCCTGCAGGAGGCAGCTGCCGCTCCATTGGGATGACCATTCCTTCTCGCTGTGCGTCCG

TGCTGCTGACGGTGCACTCGTGCACGCCCCCACCAACAGCCGGTCGTCCCGCTCCCC

AGCAGAAATATGGAGCTGTGGAACAATCCAGAGTGTCCCGGTTGCACCTCGAGGGGTC

GTACGCGTGCCGCTGCAGCCCCACCGCAGCTCCATGCAGCCCGAGCGGTGCACCGTGT

GCACGTGGCCAGCGGGCAAGTGGCCGCACGGCTCACACGCCCCAGAGACTCCCGCCC

GTACGGCTGCTGTATAAATGCGCTCCCAGGCTCTCCTCATCCCTTGCTACAATCCTCCCT

CACTGCACTCCAACACACTCACGCCGCCCTATCGATGATCACCCATACTCTCCGGCACC

TCCCCATCTTCACCCTCTGCTCTGACTGCCATCCCGCACTCACTACCTCCGACAGCTCC

CTGCGCACACGTCTTCACCCCACCCCACCCACGCCCCTGTCTTCTCGGC

>*PyyHsp20-25.9*

TCTCCACCCGTCTTGTTGGCGCCAGCCGCCGTCGAGCCGCCGCTGGTGCGGCGCTCGC

CGCCGTGCCACCGCCCGTGCGGTGGTTGCCGCCGCGCCGCCGCGCTGCGGCCGCC

GCGCACGGGGCACGCCGCCCGCAGCAGCCACCGCCGCTGCCGCCGCCGCCGCCACTC

CACACACGGGCGCGCCACCAGCCGGGGCCGCCGCGCCACAACAGCCACCGCCACCAC

CCCCACAACCGCCGCTGTCGCCGCCGCCACCACCGCCGCCGCCGTCGCCGCCACCAC

CACCGCCACCACCACCACCACCATCGCCACTACCGCCGTCACAGACAGCCGCCGTCCC

TCAGGCAGCCGGCAGCTGCTCGGCGCCTGCCGGCCGCCACGCCGTCATCGCCCGGCG

CGCACGCCGCCCCGGTGGCGGCAGCAGGGGCGCCACCAGGAGGCGGTGGCGGCGCT

GCCAACGGCGCCCGTCTTGCCCGAGGCATCCCGTCTTCCCTCATCCTCCCCTCCTCCCC

CCCCCCACCCCCTGCAATCCCTCCCGCATCACACACCGGGAGCCGAGCGCGCCGGAGT

AGGGAACACGACGATGGTGGGGGTGATGGCCTCGCCCCGGCAGGCGCCGAAGTCACG

TTGCTGCGATGCCTCGTGGTTGGGCTGTCTTGACATCTTCATGGCGATCCTTACATAAG

AAATAATTCTTCGGATTCCGAGGATTGTGACGAGGTGATACGATATCAGGATTCTTGCA

P-type

AGAGAATTCCAACCTGTCCAAAATATCACCACATCTTCTGGAATATTCTGGAGGACGGG

CCACGTGTAATGAACACGTGGTGACTCACCGCAGGGGTAGATCAAAGGGATTCGGAA

CCAGGCACCACTCGATCGCCGCGGTGCGTCACGGTCCGATCTGCAGATGCATATGAGG

GGGGAAGCCCCGACGAGGACACGTCCCGCGGGGGGATTGTGCGAGAGGATCGCCCCC

CAATCAGGGTGCGCTCCAGCGCGCGAACTCGTTGTGGTTTCGGGTGGAACCATCTGGT

ACGCCTGTGCATAGGCAGGTGTCGGGCGCCGCTGAAACGTTCCGAGGAGTGCGCACC

TGCGCCGCTAGTAAACAGCGTCCGTCATCCCAGGGGGAGGGAGCTCGGGTGGCTTCC

AGACTCTCCCGGGAGGAGCGCCCGTGGAAGCATGGAACCCGCTCCTCTCCTACGCAA

GAGCAGACCCGCGTTGCATGCACTTCCACAGCACTGGCCTGCAGCCAGCATGGAACG

CCCGGCCAACTCGTCCGCCTGGCGTCCCCATAGCATAAGAAGAGAGCGAACCAAGCC

GTGGACGCAGGTCAACGCTGGTCGCCACCGTTCCCTGTCTCCATACGCCCACCGTCTT

TGCTGCATCCACCGTGCAGACGACCACCCGCTCGTGTACTCGACGTTTCCGCCGCGCA

CTTCCGCTCTGCCTGCTCCACCCCTGCCTTTCCCGTCTCCTTCCCTTTCCCGACGTTCA

AG

>*PyyHsp20-19.3*

GCGGTTGGGGAGCCGCGGTGCGGTGCGGTGCGGTGGGGCAGAAAACGGTGACGACG

CGGGTGGCTGCCGCGATAGGGAAGGGAGGGTGGAGGGGGCGGGTCGAGAGGGGGGG

GTCGGGGCTTCCGCCGGCGCCGTACCAAACACCGCTGAATTGTCATTGTGATGGAGCG

ATTCCATGCGGCTGTCTGCGCCGCGTTTTTGAGTTGGCAGCGCCTCCTGCTCGTACGCG

AGGATGGTGATCGCGTTGGGGAGGGGGGGAGGGAAAGGAGCGAGAGGGCGGGGAGA

AGAGGCGGGAGGGTCGTTTGCGGCATGCCAAAACCTCTTCCGGAGCTGGCGTCAGGA

GCGCGCTCCCTCCTCGCCACCCCCACTCGGCTGCGGGGCGGCCAGCCCGTGGTGCGC

GACGCATCCAGAAAAGCCTGCGGCGCGGCGCTCCCTGGGACGACACGGCCACCAATC

AACCCTCGGGACGGATGGTGCGCTACTGGGAGAAATTCTCCCCGCGTCACTGCCCGAT

GGGGGAAGGAGCGACGGCCGGGCCGCCGACGACTGGCAGCGGAGGCTGGCACGGTC

CTCTCGGGTAACGTCATGTGCGCATGGGTGGGGGCCTCATCTGCAGGAGAGTATAGGA

TGATTATGTAGCATGGACATGGGACGCGCGGGCTCCCAGCAGAGTATGTGCTGTAGCA

CCCAGTACAGTACAGTATAGTGCGCACCCATGTGCGCCTGCATCATAGGGCAGCCGGTG

AGGGTCCTACGTCCCCGACTATGTATTGGCGCGCCCCCACGGGCGTGAGGGCGAGGGG

GGAAGGGGCTCTCTTGTCCCGCGTCTCTCTCTCCGTCTCTCTTGTCCCGCGCCCGCCCT

GCAGCCCCCAAGGGGGAGGCGCACGTGTTACTTGCCTCCGAGGGGCGCAAAATAATTT

CGGCTCGCTGGTCGTGCTTCGCATTGACATTGCGCGCCCAAGTCCAGACCTTTCCAGAT

CCGCACCGATTTGAGTGGAAGCGCCTCGAACCGCCCTGCACGTGCATGGTTCCGCGCG

ACAGCGAGGATCACCTGCTGCCCCATTCTTTCGGAGAAGGTTGATCCCCCCACGTGCA

CGCACCGTCCAGGGCTTTCGTTCCCTCCCCAAGCTGAGAGTTAGGCTGTGGAACGATC

GAGACTCTCCTAGGGGCACCTGGCAGAGGCCGTGCACACCCCTCCCGAATGCAGTTTG

CGGTGCGCGCGTGCCCACACGCCCACCTTGGGCCGCAGGCGCCAGCCGCATGGCGAG

CTGGCGCTGCGCCTCGGCAGGTGTGCGGTAGCTATAAATACGGGTCCAAGTGCAGCCA

GCTCGTGGTTCGAATGGTCAACGCACACACCATCCTCACTCGACCAACACCCACGAAC

ACTCCTCATCCCTCCCCGCTGTGCGTTTACCACCCTCTCTCCCTTGCTCTCTGCTCTCTC

TGCTTCCTTCCCCTCTCATCCTCTCCTACCACCTTCCCCCTTTCTCCCACCACGCA

>*PyyHsp20-19.2*

GGGCGGCGGAGGCGTTGCGGCCGGCAACGACAGGCACATCGCGTGCCGGCTGTTGCG

TTGTCGTCCCTACCACAGCGATGCTTTTGACGCCGGTCCCACTGGGCGGGGGTGTGCC

GCGGCTTCCAGACGCGTCTCCGGTGCAGGGGCCGGTGGCCCTGCTGTTCTGTTTTTG

TTCCGGTCCATGGCATCGCCAGGCGCACGGCGTGCCGCGGGGGCACCCCCCCCGGTT

CGGCTAATGGCGACCCGAGGTGCTGCTGCTCCCCGCCGTGCAACCGCGTTGGGAGTCG

AGGGGACGAGGACCCCTACGTGGAAGATCCAGCAGGGCGGGTTGCTGGGAGGACGCC

GACGGGGAAAGCCGGCGGGTGGAATGAGAGCGCTTGCTGGATGAGCCCCGCCCCTAG

TGTTTATAAAGTTTGCACTGTACTGTGCGTTGCGTCATCCGTCTTTCCCTCAAGTCCCAT

GGTGAGCCCTACTTTGTGTCTTTTTGCTCTTCCCGCCGGCGCATGCTGTTCGGTTTCCG

CTGTATGTCCCGGGCTGGAGACGCGTGTCCGCGCTGTTCAAGGAGGTCGCGCGTCCGG

CGGCCGCCACGCCTGCGAGGCGCTGTTACCGATGGTGGTCCTGCCGCCGCGCCAACCC

ATGTCTGCATCTGCAAAAAGAAAAAGCACGTGACGCAGGGGCTTTCTGTATGTCTGTA

CAGTACGCACCCACATCCTCCCGCATCCAAGAGCAGGCTCTGCCCAAGGAGGCCCTAC

CGTCACAATCACCTCCGTCCTCTTACGCAAAAGAAAAGGAATGTGCGGCACGAGACTC

AGGGTCTGGAGCACCTTGTGGCTTATTGTATGTACTGTACTGTACTGTACATACGTATCC

GGGTACACGTCACAGAAGTCCGCCTGCAAAAATTTGCGGCTCTCTGGTTGGGCATCGG

TTGCGCGCCACGGTCCAGACCTTTCCAGATCCGTCCCGATTTGCGTGGAAGGGTCTTG

CAAAGCCTCCTGCGCGTGCATTGGTTCCGCGCGGCAGCGGGGATCACCTGCCGGCATG

CCCCATTCTTGGCTCCGTGTGGATGACGTGTGGATGATCCCTGCGTGCGCTCCCTGCGT

CGACGCACCGTACAGGGCTTTTGCTCCCATCCTCGAGCCGACAGGGTTTAGGCTGTGG

AATGATCGAGACTCTCCCAGGGGCGCCTGGCGGAGACCGGGCACGCCCCTCCCAGGT

GCAGTTCTGCAGTGCGCCTGCTCACGCCGACCTTGGAGCCACCGACCGCAGCCCCGC

GGCAGCTGGGCACAGCTGCGTAGCTATAAATACGGGTGCCGATGCAGGAGCACGGTAG

CTGGGTCGGGCAACACACACACCATCCTCACTCGACCATCACGCGTACGAACACCCCG

AATCTCTCGCACGTCGCTCTCGCCACCCTTTCCCTCTTCTCTGCTATACCCACTTCCTTC

CCTACTGCAACTTCCCCTGCTATCTTCCCCCTTTCTCCCATCACACT

>*PyyHsp20-37.6*

CTCAGGTCATCTCTGTCAAGAACAGTGTGAACCGCTCAGCCGGTGCCTGTTCTCCCCT

CGAAGAGCTCAGAAATCCCGAGAAAATTCCATGTCACACTGTTCCTACAACAACGGAC

CCCCATGTTGCAGAGCAGTCATTGGCCAGAGAGGCAACGTCTGAGTCATACCATTGGT

CAGATACAGGTTGTTCTTACTTGTCCATAAGAGACAAAATTGGGCAAACTGCAGCATG

CCAGTCAGCTCAAAGACAAGAGACAACAGTGGTCGACCAATGGTGTGCCTAAAATCT

CTGGCAGTCTCGCATCAGATCTGTCGTTGTGCAAGCAGGGCAGCCGGAGACTTCGACC

GATTTCTGAGCTGATCGACAAGAGAACCGGCAGCGGCTGGTGTTTCCGAACTGCGATT

TCCTGAGGCTCGCCTGAGATCGAGGCTGAAAAGGGAAAGTCAGGAAAAAAACGACC

GTATTCAGGACCTGCAGCCAATCGCATATTCTGGGATCTAAGAGACTTTGCATTACTGC

GCAGGTCTCATGCTGTTCTCGACTGTCATTTGCCGGTACGCTGTCAGCCACCAGTTGTT

GTGCAGTTGTACCGTACGTCCTTATGCACAACCGCCAAATTTGGGACTTTTACATTAATT

CATTGTTGTTGTCCCAGTTTTGGCCGACATGGACAAGAACATCAGTGCCATCCAAGAG

TGTATCATGCATTGATGGTGCCGGATACAGCCGTCAAGCTAATGGGCTGTTGTGCAGCA

ACGTGGTTGTTCTTTTCCACATCGGGCAAATTGGGTGAACAGCAATATTATGCACTAGC

GTTACTCACGACTGTCTTCAGAGAAACCAGCCGAGATGGGAGAAAACTCCGACGCGA

TCAGACGAGAGAGCAACAGGTCTGGATCAATATGTGGCAAATATACGGATAAAACCGC

AGCAGAAAGAGCTCGTCGAAATGCATCGACAGTTATGAGCTGTGGGTGTATGACATGA

CACGTTCGCCATGTACGGCCCTCCGACCCAGTCCGCTATAGTGCGTCCCGTAAACTGAC

AGTCCAACGGAACTACTCGGAAGTACTCTCGATCGAGATTTCACCCGTTGAAGGTGTT

CTCGGTGCGGATCTAGGTATCAATTGTGGGTGCTGGGCGACCCAGGATCCACCCACAG

CTGACCCAGATCCATGCCTGGTGAAAGTATCTTACTTCGGAGTCGTTTTCATGTTCGAG

ACACTGCGAGTGTGCCCACGTGCCCACGTGGACTCGAACCTGCCATCAAGGCCATAGC

TTCGAACGATCCTCCAGCATTTCGAGCCCCCTGGAAGGCCCTGGAGTCCGCTGGAGTC
ATAGACTCCGCCAGGAAAGACGGCGTGCGTTGCCCCTCCGCTCCGTCATGGTCACTCT
GCGGGCTGCCGCCCCGCGTCACCCGGCACTGTGGAGGGCTGCGGACTCCACGGTCGC
CAGTCGCTGGTGCTGCGTTGGTCACTGCCGCCGTACAGTACGCGTCG

>*PyyHsp20-37.4*

AGACAGGGGTGGTGATGGACGGCGACGAGGAACGGTGTGAGAAGTCGTCGGCGAGA
CGCCAGGACTTGCCAGCACCTGGGGCGATTTCGTACCTCATTGAATCCAGATCGCCAC
GTGTCGGTAATATACGGTAAGAAATTTCCTGACCGGTAATCACGCAGGAAGACACAAC
GGGGAGGGGTCGTGACATGTGATGGAAAAATTCCATACGTGGCGGTCATTTGTACAAC
GCTACACTTGTGTGTCCCCCAGCTGCAACAAGAGGAAGGATATTGTGCGTCCCGTAAA
AAGTGGACGTGCACAGGTCGTGCAGTGTGGGTTTGATTGGTCCAGTGGCCGACATTTG
ACATGATCACGACACGTGCACGTGTACTTTTTACGGGACGCACTATACGAAGCGCATAC
GTCTGCCATACCAACGCTGCATGTTAGTTTGAAAAGCCGTATTCAACGGTACCGTCCCG
AGACACACCAAGGAGTACTGTGGAAAAGTTTCAGCTCGATACGATCATTTGCGACGAA
ATAATGCCGCTTTTGTGACCGGCCTAGCTATCTAGATATATGAAACCCACCTTCAGTCGA
ATTTGCTGTAGTCAGGCTGAGAAGGGGCGACCATGTTAGAGGACATAGGAGCTGGCTG
CTACCTCGAGAAGAAAGTGGCGCTGTCAGAGCGGGATGTCAAGGCTCGTGGTTTCGT
GCAATATGGGACTCAACGTGTCTCTCGGTGCAGCGCGCGGCGCGGCTCTGGCGTGCGC
CGCCCGGTCGTCGGGGCCTCACGTCCGTTGACAAGCGTAGCGATTACTACAGTCATTC
AAGGACTCCGCTGAGCAAAGGTGAAACCATACAGCGCCACTGTCCCCCTTCCAGTATT
CCCGATGGCTCGCTGGGTTGCCATTCAACCAATGGGCCGTGCGTCCGCCATATGGGCTG
CAGTGAAGGCGCTCGTGGTGCACGAAGCGACTGCCAGTAGTGATGGCATCGAAGGCA
AAAAAGCAACCAGTCAGGTCCCCTGAATGCGTGCACCTGTGCTGAGGGGCTTATGTAT
GACCTCACCGAAGCTGGCGACAGTCGTAGTGGACACAGGTGTACGACGAAGTGCGGG
CGTGGGATTTTCTGAGCCTGGCCAGCCTGCGGAATTCATGTACTGTACCGCATGCATGT
ACTGTACAGTACTGTACCATTATACATGTGGAAAGTGTGCGAAGGCCATTGATTGGCCC
ATCAATTGACATCAACAAGACGGACAGCGGGGCTTGGACTGTTGCTGAGCTACGAAG
GTGGCTGGGTTCTCCGCAGAGCTGACCTGAGGGGAAGCGGGTAGTGCTCGCCTCAAG
GAGTGCCTCGCGACGCGGTGGCTCTGGTACAAACTTTCTTCAGCGCGAGCACTTGGCG
CTTTGTCTGGGCGTTACCGTCGGCGTCGTCCGTCGAAGCTCCTCCTCTGGTCGCTGTTC

ACTTCCCGAGCGCTGTCCCCCTCCGCCTTGCCGTTCCCCGCCTCCAGCG

>*PphHsp20-25.9*

GGGGCAGAGGGAGAGCACGACCGCCCCGCGGCCGCGGTCGCCCGCTCCGCCGCCCGC

GTCGCACTCCCCCGCCACCACGGCCACCAGCCCCTTCAAGAAACGGCAACCTCGCTG

CGTTTCTCCATTGAGCCCGCTGCCGCCGCCGCCGCCGCCGCTGCGGCATCCGTGT

GCCCCGCCGCAGTGGCGCTGCCGCCCGCCGCCCGCCACCGTTGCCGCCACGACCACC

CGCGCAGCGGCGGCGCCAGCGGCGGCCGCCCGTCTAGGAGAGCGGCGCCGCTGCCAA

CGGCGGCGGCGGCGGTCGGTCCGCGCCCCCCCCCCCCCCCGACCTCCCCGCCCGCC

TCCTTCCCGCCGCGTGCAGCGTGGATGAGGTGGCGGCGTCCACCGCGTCGGCGGCGG

CGGCGGCAACGGCGGTGGCTACAGACTGGGGGGGCGAGGGGCGAGATGGCCGCGTG

GGTGGCCGCTCGAGGCCGGCCGCGGGAGTTGGGGAAGGCAGGGGCACCCGCGCGGC

ACGCGCTCCATCCCCGCCGTCGCGTGGAGGCCGCCGCGGCGGCCGCGCTGGTGGTGTT

GGCCGCCCCCGCCGCGCCGCCGCGGCGAGCGCCACGCCGGGCCGTGTCGTTGGAGCG

AGGCAGGCCACGCCGGCCACGTCGGCGGGGTGGAAGAGGCCACGCCGGCCACGTCG

CTGGGGCGGGGGGCGGCCACCTCGTCAGGGCGGGGATGGTCACGCTGGCGTCGACAG

GTGGGGACGGCACCGCGGCGTGACGGTCCACAGATGCGAGGCCACCGAGCGCCGCGC

GGCCGAGAGGCGGCGGCGCGACGCTGCTGCGCTCGAGCCACGTCTCGTACGAAAACC

AAAATGACAGTTGTCTGAAAACTTTCGCAGTTTGTTCTGGATTCTTCTGGACCACGGG

ACCCTCACAACAGCGTGGGATCAAGGGGTCCGCCCCACCCCCGCAGAAACGGCTTCA

GGCACCACTCGCTCGCCGCGGTGCGTGCCCGACCGGGCGGTCTATAGGCGGGAAGGG

AGTTGGGACGCGGCGGGGTTTTGGGTGGAAGCAGCACCTCGCCGCTCCGTCCAGCGC

GGGGGCACGTCACAAGGGATGTCGGGCTGCGGGCGCCTTTCGGCCATGGAGGGCGGG

GGGTCCGCGTTCCAGGTTTGTCTGGAACCTCCCGCGGAGAGTGCCCGTGGAAGCAAC

ACCCCAACTCCGTTCCCTCGCAGGGGCAGACCGCGGTGGTGGCTCTTCCGCGGCATCC

GCTGCCGCCAAACTGCCTCCGGTGGCCACCCCAGTCTGGACGCCACCACAGTATAAAG

AGGGTGCGCTTGCCCAGACTCCTGTCGCGACTCGGTTCGGCATCGACCTCTCCTTGTC

TCCCACCTCCTGTGCCGCATCGACGCTGCAGACGAGCACCCGTCTTTGTGCTTACCTCT

TCTAACGCGGGTGTACATACAGAGCGGCTGTCACCCGGCTACTGCCCTTCTCCTCCTCC

GACCGTCAAC

>*PphHsp20-19.2a*

CCCTACAAGTGTACCCTGCCTTTGTCTCACTTCCGGTGTGCGGGCCTCGGCGGCCGTC

GCCATACTCCATAGCCTCCTCTGTCCGCTGAGGCTTCCATCGACGCAGGGCCGATCCCA

CCCGCGTGGACCGCTGCGGCCCAAGACTGGCTGCTTGTGGCTTCCAGCTGCCTCTGCA

GCCGCTTGTACAGGTATGTTGATTGGGAGGTGGGTGACCGTGAGCACCAAGCGTTTTG

CGCGCGCGAGACAGAGGTATCACATGTTGTGGTGCCTCACAGCGAAGGCCTCTCGCCG

TCACTGGATACGTCACGTCGTCTGCTATTGTGATTCTCGCTGCCGTTGGTCCCAACATA

ACCGTCGCAGCAACTGACTGGTCTTCAACCGTGTCCCCCACGCCCTCCCGTCTCCGGT

TTTTCTTTTTCTGTTTGGACCCCCTTGGCTAGTAAGCTGCATGGCGCACTGCGTGCCAG

S-type

CGTTTGTTTTGCCTGGATTCGGCATCTTTCATGCATCTTCAGGACGTCGGGTGCTACCC

AGGACCTATGTACCTTCTGGTCCTGCATCACGTCGACGTGGTCTTCCAGGCCGATATGG

GCCGCGCAACCCTCGGACGCGCCCTGCGCACGCCGCGGGATTGGGCACCGGGCTGTC

TGTTGACCTGTCTGTGCCCGCGGTGGGTATCTCTGTCAATATCACGATGGAGCAGGAAC

TGCAGTCGGTCTTTGTGGGGGGGGAAGGGGGCGGGGCGGCGGGAAGGGGATGGCTG

CCGATGGCGAGGCGGCGCCGCGTCTCGCCGAGTCGGCGGCCGGCGTGGCGCGCGGCC

GCGACGACCGGATTGATCCGCAGGTGGGACTAACCCGAGAAAGGGGGTTTCATCCCA

CCTATATTTTGCACACAACAAGTTTCCCCGGTTCCTCCAATCAGATCCGGAGCGCTCCC

S-type

GACAGTTCCAGGCAGTTCCAGACGGTTCTCCATTCGTCTGCTGATTCGAGTGGAAGCG

CGCAGCGCGCCGGCAGGCGGGGCTTGATCTCTGGCGCCGCGACGGCGCGGCCGCGGT

CCGTGCGGGTCCCCCCCCCCCGCCGACGCATCGCGCACGGGGCCGACGTCGGCAGC

GGCGCGACCGGCGCGCCCCCGGCTGCGCGTTTAGGGTGTGGAACGGTCGAGACCCGT

CGAGGGGCATCTGACGGCGGCCGCGCGCGCTGACCGAGAGCGTACCAGGTGCCCCTT

GCGGGGCGGGCGCAGCAGCTGCCGCTCTGCCGCGGGCGGACGCGCGGACCGCGCAG

GCACCCAACCGCTGGGGATGTGCCTATAAATACGCTCCCAGGTGAGGGTCGATCGGCG

TCTCGGTCGCTCACCACACTCACCCATCACCACTCGACCAACACGCACACATACTCCA

CCATCCCTTCCTCACCACGCACAAGCCAAGCTCCCCCTCTCCCTCTTCAATCCCACCTG

TTTCCGGGTTGGCGCGCCCGCACCGGTCTACCACGCCCCTCTCTCCCACCACC

>PphHsp20-19.2b

GCGGCGGCGGCGGTGGGGTGCCCCCCCGCCGCCGCCCGTCGACGGTGCGCGCAC

CTCCTCCGCGAGCACCGCGCCGCGCTGCCTCAGCGCCGCGCGGCCGCTCCTCCTCCCC

CTCCCCCCCCCCCCCCAGCACACCCCCCCCGCCTCGGCCGTCGCGCCGCGGATATACG

TCGCGCGCCAGCTCCCCCCATACGCCAACACCCCCGCCGACGGGTGCAGCCGCCGCA

GCACCGCCGCGCGCCACAGGCCCGCGTCACGGGACGCGGCGTGGAGGGTGCGGGAC

GCCGCCCCCACCGCGTCGAGGTCGGCGCCCGTCGGGAGGGCGCCGAGGACGCGCAGC

AGCAGTGCGTCGGGGAGGGCGGCAAGGGCGCCGAGGTCGGCCGCCCGGTGGGAGGG

GCGCGCGCCGGGGAGGAGGGCGTTGCCGTAGGGCTGGACGTCGCGGAGGGGAAAAT

GGGGGGGGGGAGTACCCCTAGGGGAGACGGTGGAGCCGCCGCCATTGGGGGGGGG

GGGCAGTCGTAACAAGAGGGGGGGGGGGGGGGCACGGGGAAAGGCGGTGGGGGG

GAGTGGGCAGGGCGGGCGGAGGGGAGAATGGGGGGGGTTGGAGCGGCAAGGGGAC

GGCAGCAGGAGGGAAAGGGATGGTGAGGGAGAGGGCGCGAGGGGCAGACGGCCCC

GGAGGGCGCAGTCCGACAAAGGTGACGCCGGGAGTCGGCGCTTGGGGGGGGGGAA

GGGGGCGGGGCGGCGGGAAGGGGATGGCTGCCGATGGCGAGGCGGCGCCGCGTCTC

GCCGAGTCGGCGGCCGGCGTGGCGCGCGGCCGCGACGACCGGATTGATCCGCAGGTG

GGACTAACCCGAGAAAGGGGGTTTCATCCCACCTATATTTTGCACACAAGTTTCCCCGG

TTCCTCCAATCAGATCCGGAGCGCTCCCGACAGGACCAGGCAGTTCCAGATGGTTCTC

CATTCGTCTGCTGATTCGAGTGGAAGCGCGCAGCGCGCCGGCAGGCGGGGCTTGATCT

CTGGCGCCGCGACGGCGCGGCCGCGGTCCGTGCGGGTCCCCCCCCCCGCCGACGCAT

CGCGCACGGGGCCGACGTCGGCAGCGGCGCGACCGGCGCGCCCCCGGCTGCGCGTTT

AGGGTGTGGAACGGTCGAGACCCGTCGAGGGGCATCTGACGGCGGCCGCGCGCGCTG

ACCGAGAGCGTACCAGGTGCCCCTTGCGGGGCGGGCGCAGCAGCTGCCGCTCTGCCG

CGGGCGGACGCGCGGACCGCGCAGGCACCCAACCGCTGGGGATGTGCCTATAAATAC

GCTCCCAGGTGCGGGTCGATCGGCGTCTCGGTCGCTCACCACACTCACCCATCACCAC

TCGACCAACACGCACACATACTCCACCATCCCTTCCTCACCACGCACAAGCCAAGCTC

CCCCTCTCCCTCTTCAATCCCACCTGTTTCCGGGTTGGCGCGCCCGCACCGGTCTACCA

CGCCCCTCTCTCCCACCACC

>*PphHsp20-19.1*

GTGGCGGCGGCGTGTGCCACGCCGCGCCGCTGCCGTTCGCGCGACTTGGCCGTCGCT

GGTGCCCCCGCTCTGGCGGCCGTGACCATCGCCTCGTCGCATGCGCGCCCCCTCCCGC

CCACCACTGCCGCCGCCCATGACGCCGTCGCCGCCACTGCCGCCGCCGCTGCTGCCTC

GCGCGCCGGCGTTTCGAGCCGGAGCGTGGGCGCCCCATCCCCCCTTTCCCCTCCTGCT

TCTCGGAAAACCACCTGCGGCTCCCACCATGTGCACGCCACCCCCCCGTCGCGGCGGG

GTTCGACGCCCCCCGCTTTCTGCGGCCATCGCCGCCGCTATATAAATTCCCGACAATAC
CTGCCCTTTGTTCACGCCCCCCAATGAGAGTAAACAGCGTGCCACCGCGGAGGCAGG
AAGGCAGGAGAGGAGAAACAAAGCAGAGAGAAGAGAGAGAAAGAGGAGAGAGACA
AGGCGGCCGACAGAGACACCGAGGGGCTTCAAGCTGGAAAGGTGGGGGGGGCGCGA
GGGAAGACGAATGTGCCGGCCGACGACGGCCGGTGCGTGCGTCCCCGCGCCTCGCGT
CTTGAGCACCGTCGGGCGGACAGGTGGCCCCGCCGCGCCGGATACACCAAGTAGTCC
GCCGGGGAGATTGGGATCCCACCCGCTAGAGGCGGACGCATAACGCCTTAAACCTCAC
AAACAAGACACAACACGCGCGCGACGGGCCGCGGCTTTCCCCTCCTCGAGAGCCGCC
CCCTCCTGCGCATGGACATGCACGTACCCGCACACACATACGTACGTACACCAGCCGAT
GTGGGCCCTGCAGTCAATGTGACGGCGCGTGGCCGCTGGGGTGCGCGCCCGCTTCTCC
GCGTGACGATGCAGCGGGGGGTGGCGGTGGGCGCCAAGGGGCGCGGACGGAAGCGC
TTGCCTACAAACTGTTTTGTGCTCTCTGGTTGGGTGTCGTCGGCGTGTGCAAAACTCTG
GACTGTTCCAGATCCCTCCCCGATTTGCGTGGAAGCGTCTGTCGCGGTTCTGGGAACC
AATGGATGCTGCGGCTACAGCAAGGCCTTCCCACGCTCACAAATGGGATTGATCCCGC
CACGTGGTGCCCGCTCCGCCGACGCACCGTGTGAGCGCCCCGACTTCCCGGCAGGGT
TTCTTTGGCTGTGGAACGATCGAGATGCTCCCATGCGCGCCGCGCAGAGGCCAGGCGC
GCTCCTCGCCCAGCTGCTTGCGGGGCGGGCGCAGCCCCGCGGCACGTAGGCCTGCGG
GCAACACAGGCGGTGGCCGCTCCCATGCTGCGCGCAAGCGCGGCTGCTCGGCTGTAG
CTATAAGTACGCTCCGAGTTGCGGGTCGATAGGCGCCGCTCTCGGTCATCGCACTCACC
CATCCACACTCGACCAAGACGTACAAAACACCCCATCATCCATCCCGCCGTTCTCTGG
ACAACCTCCCCCTGCCGCGCTGCACTTGCACCTGCTGCCCTACCCCCTTTATCTCATCA
AG

>*PphHsp20-28.5*

GACACGGCCCCCTCAACCCCCGACAACAGCTGCTTGAACTGCTGCGCGTCCTCCTCAA
CACTGCTGCCCGCCTCCGAGTCGCGCATGCAACCCGGTCCAGCTCGGAAATTGGCTCG
CCAAGCACAAAGCGTCCGCGACAGCCACTGCCGAGCGCCCTTGTGCAGGGGCACAGC
CACGGACAAGCCCAGCCAGGAAAGAGCAGGAACTGGCTGTTGACGAAACGCCGACT
TTTGCGACGACGGAGTGAGCGGGGAGAGGGCGCCGTCAGCCGCAGTGTCGGACGAG
GTCACCCGGTTGACGGCCGGCACCGGCAATGGTGCATCCTCCACGTCCATACGGGCGA
GTCACCGCACAAAACAAGAGGCATCAACGGGCGGTCACGGCGGGAAAGACGGCCGT

CGCGGCGGTAGTGACAGCGGGTTGCAGGGGCGCGCGTCCAGCAGCGTGGACGAACGC

CGGGCAGCGCCCAGCTCAGCGCCGGCGACGAGGGCCAACGCGGCCCACCCTGATATC

TCAATTGTGGCGAGGAGCCAAGCAGGCAGATACGGGAGCGAGATGAGCGGCCATGTG

CGCATCGGCAATATGGTGGCAACCTACATCGCGAGTGCCGCTGCCCCTCGTGCAGCGG

GACACACCTGGCACAGCAGGACACTCCCTCCCAGGCACGGAGAGGGGAGGTCGGTC

AATGTCGGCTAGGCGCGGCAACAGACAGAACCCAGCGCTAGCCAAGCTGGGTGCGCT

GCACGGCGTCCAATGATGGCGGGGTGGAGGGACGAGGGGGCGGGGAATAACATTGTC

AGTCGGTTGGTATGCCACGGGCGTGGTGGGCACGACCGGGTAGCTCTCCGGCATGGCC

TTCGCACACGTTGCACAGTAGAGAGCGCTTCAGCAGGAAGGATCTGGCAAGAGATCT

CGATAATTTCTCGGTAGCGAGGCAGCAGAATATTCTTACCAGAACTGAGGCTGCGGGA

CCTCCCTAATTCTTTCAATACAATTATTGTAAATGTGACTGACTGGTGTGCTGCGAGGCG

ATGTGGGTGACGTGGACTGGATGTCGTACATCACACAGTACGCCATAGCTGCAGACTTT

CGTTCCAGCGCTCCAACGGACAGCAAGGCCTAGAAGGCGCCGGAATCTCCGACGTCG

AGATGGGCGGGGCTCGACATCGACGTGGTGGAAGGCTCCCGACTCCAGCTCCACAGC

CGCCAGCTGCTGGGATGAGCGGGGGGATCTGGTACGCCACCCGCGCGTGCTTGAACTT

GGCTTGAACTGGATGGCCTGGTGTGGCAACTGCAGAGTATGAGGCATAAAAGCGGGC

GTGTGCCCATCAGTTGGACGTCCTGAGCACTGCATCTTCTCCTCACTCACACACCTTGT

TCAGCGCCCACGACACGCCACACCCACCTGCCGCTGTTCTCCGCCACTCGCCTCGTCT

TCCGCAAGCCTCTACTACGGACGCCCCGCTCACCCCTGACGCCCCGACGCCTCCCGTT

TCCACC

>*PphHsp20-15.6*

GGCGGCGGCGCCGCGTGGATCCCCCCGCCCATCGGGCGGGTTTGCCGTCGTGGACGC

GGCGGCGGGCCGCCGTGGCGGCGGGTCTGGACCGGGCGGCACGCGCTGCCGCGGCA

CGCGACGCTTGGCGCTGTCGATGGAGCGGACGCGTCAGGCACGCGGCGAAAAAACGG

TTGTGCGGCGAAATCGAGACACCAATCCGAATGAAGTATCCGTCCATAAGAAATACTTC

TTGTAATTAGTTGGTCTCACACTTGGTTTAAGATATCTCGGTACTGTGTGAGATTTCCCT

AGTCGTGCAATAATTTCTTGACATCAAGCTGTACAGGGATGATCCGCATCAGTTGACAT

CTGACTTATGCTGTCATATTGTCACGACATGTGTCGCCAACGATCCGACTCCACAGATG

CGTTTGGACTCTTCCAGATCCGTGCAGAACAGGCGTGGCCGCTCTCAAACGCCCATTC

CATTCGGCCACATGGGCGGTGGGGCGGTGCCATCTCCACGTGCGCGCGCCTTCCGCGC

TGCCGGCGGCGCGCGTCCCACGCTCCAGCAGGACTGTTGAGCTGCAGACCGATCCAG

AGTGTTCCAGACAACCGCGCCTCCGCGACAGGCGCACGCCGCCGGTGCAGTGCGCCG

CACTCGCGGACAGCAGGGAAGTGCCTCTGGCGCTCACGCTCTGTGCGGGCCCCACCC

CTGTTGACGAACGACCGTGCCCCTGTAACGGGGCATCACGAGTGAAGATCGCGAGGC

AAGGTGGAGTTGACCGCCGGCGGGACATTACCACCAACTTGGTCATCCAGTAGCCGGA

CAAGTCCCGCGCGGGGGGGGGAACAAGTGCGGCACCACGAGAGGAGGGATGATCGA

GCGAGAGAGACGCGTGGCCCACGGTGGCGACTGACCAGACTAGAGAGGGGCACCCG

CGAGACGATGTGGGGCCCGCCGTGTGGAGTGGGCAGGCGAGGAACGGGGACCCCCAT

ACGGAGGTCCTCGGGGGGACCAAGGCGCGCCCACACTGTTGGGGCGAGTGGGGGAG

TGGACCGGCGAGAGACACCGACCCCTGAGTGTGCGTTGAGGGGGGTAGCCCCAGCGA

GGCTGAATGAGAGAAAGGGGGCTGAGGCAGACGCGGGCGCGCGGCGCCACACGAGA

GAGGAAAAAGAGCAACGCGTACTGCACCTGATTGTCAACAACCCCCGCAGGTTCAGC

TATAAGTATGCTTCCAGGTTGTGCTCCACCTTCGTCCTTCTCGTTTTCGTTCGGACACCA

ACACTCTCACCCCTACTCCTCGACCAACAACCTCCGACATCCCGCCTGTCCTACCCTCC

GTGCTCTGACAACATCCTCCCTACTCCCCACCACCGGCATCTCTGTGTAGACGCCCTCA

CCCCACACTACCCCCCCCACTCGTCGACATGGACGTGTTTGCAGTGGACCTGTTCTGC

CCCCCCACCGCGCCGCAGCGCCGTGGGCGCAGCGCAGACCCGTGGGGTCTCTGGCGC

CCC

>*PphHsp20-20.8*

GGGGGGTTAGAAAAGGTGGACGCTATTTGACAGCGCCAGCCTGCGTATCGTGGCCGTA

CAGACCCCCTTGCGTGCACGTTGATAGGGGGGGGGGGGGGGGGAGGCGAGGGACTCG

ACATTCACCGTGATGCGGACGGCCTGACTCACCCGTTCTTGGCCAATGGGGAGGGGGG

AGGGCCGGCAGTGATTGGCATGGAAGGGCGGACACCGCAGTATGGAAAAATAGGTCT

CCCCGCCGGGGGCTGGTGCAAACGTGCGCAACCAGTGCAGCAGCACACGCGGCGAA

CGCCCGCTGCGCTGCGGCACGCCACAGCGGGGACGGGTGGGAAGTGCACCAGTACCC

CCTGCTAGTAGCGACGCCGTAGCTGGCGGCGAGCCGGGGCCGCCTCTTGCGCTCTGGA

TTTGACGGGTGGCCCATGACAGGGACTGGGCGGGCCAATCATTGGCCATGATGGGCGG

CAGCCATCCTCGCGGACGACGCGCGCTGTTGAGCTGGCACGGAGCGTGCGTACTTGCA

TCGTAGGGCGTGTGGGGAAGGACGCGGGGAAGGACGTGGCGTCTGTTGAGGGTTGAT

ATGGTATGGGGGGGGAGGGGAGGTGGAGTCCTTCCCCTTCACCGGGATACGCGGCCG

AATGGGCCGTATCGCAGCCACCGGAGCTTCACAACGCTGGGCGCGCCATTGGCTGATG

TATTTTTGAGTAGGTTGCTACGTTGGCTGGCTACAAGTCCGTTGCGGCTGTCGCCAAGT

CAGCAATCTCGACCGGGTGGACCCCCGGCGTCGCATACAGTAGAGGGGGCACCGCTC

GAGAGGGGCGGTCTGGTCCTTTCTCGGGGCAGGCTGGTGCTGTGGGTGGTGGGTGGC

GTGGGCTCGCGAAACTTCCACCGTGGCGCAAGCGCTGTTTCATCGCAGTGCTCCTCGT

GATCTACTCCACTGTTAGCCGTTCAAATTACCCACGGGAGACAAGTTCGCCCGAACTC

GCAAGTGCAATACAAGTCCAGCTGGATTGGTGATGGAAGCCGAGATGTGCTGAGACAT

CATTGACAGCGAGGCGGGTACTTCCAGAGTGTTCTCGCACGTACTTCCAGAGTGTTCT

P-type

GCATCGTTTTCTCGAACATTCCGGTGTGCCGTGCGTGGCTGTCTCACCCTGCTGGCCTG

GTCATCCAGCAACTCGAGCGGCTTCGCGTGTACGGCTGCTCCCCGCTTCTCGTCAGCA

GCTGCTGTCCTGGAACGGCCCGGGCGTGGAGAGAACCTGTCCTTGAACAGGTCTGAT

GCGTCCCAGTCGCCTACATAAGCGGTGCCGTTAACTGTTCCAGGTTCACTCCCCGTTGT

CCGCATCACTACCTGTTCCCTCAACTGACTTCACGCCGTCAACCTCCCCTTCTCCCTCC

GCCTTTCCCACCGCGCGCCCCCCCTCTCCTCCCCCCTTTCCCCTTCCCTGCGGCTGCAT

CACCCTGTCAAGACCAACACCCCCCCTCCCACCCCGTTCCCGCCTCCCGCTGCCC

>*PphHsp20-31.9*

CTCCAACAGGAGGACAGAACAGAGCATCTTCGCGCGAGAGTCACTCGAGGATCGCTC

CTGGAATGCCTGTTGCGATGTCCGACAGCACGTTAAGTGTTCGAAGGTGCCCGAGAAC

AGGTGGAAGAAGCAAGCAAGCATCCCGGATCAGTCAACCTTCGATGTTGCAAGCAGC

AAAGTCCAAAGCATCGCACTGTGGTGGAGCTGCTGTTTGAAGACATGTCCGTGAAAGT

CACAGTCCGACATGGCTTTGATGACGATGATGACCTTGGTGACTTGCTCGTCATGGCCG

CCATGGTCATCACATCCACGAGCAGAAACACAGCGCTGAGAAGCTGCCGCTACAGTCA

CACCGTGCAGCGCCAAGCTGCTCTCGAATTACATGTCCGTGAGAGTCTTAGTCCATGGT

CTTCACGAGTGTGAGGACCTTGTCCACTATGGTGATCATGCTCTTCAGAAGTACGACGC

TCACAAGCTGCCGACTTGGTCACATGACGAAAAGCTGAAAGCTTATGTTGCAAGTACG

TTGTCGGCCTGATGTGCTTGCTCTTGCTAGGGTGTGATGCCCTTATTACGGAATCTCACA

CTCCACAGCAATAACGTCAGCGCAACATCGGACGTGCGCGATGATAACTAGGTCTTTCT

GTTTCAGGCTAGGTCACGTCTCTCCACGCTAACTGCCATGGACCTGCGCTGGCTCTCA

ATGCGGAGAGCATCGAAGGAAGACTACTACTCTTTGCTCCTACCGTGGGGCACTACGA

GAGGCCCAGAACAGGAGAAAAAGCAATATAATGAACACAGGTAGTCTTCAGTGTCATG

GTCTACATCCCCCCGCAGGGCGCGAAGGTGGTGGTCTGTTTCTAGGTCCCACTTTGCA

GAGCGAGGTGGGAACGGGAAGGGAAGTGTAATTACTTTAATTGGTGTGGTCGGAACA

AGAAATACCCACACTCTGTCACGTTTACGAAGAGGCTTACTGATAAATTTTGGTATTTG

GGAGTTTGGAAGTACTTGTTCCGTGCCGCAATCATGGTCATCAAGCACCCCTGGACTTT

GATGGGGGCTACATCTGTGAAATCGACTAGACCGCCATCGCCATCAACCTTGAGCCAC

ACGCGGATTTTGTCTTCCAGCGTTGCCATGGAGGTCCGAGGGTCTTGTCGTCGAGGTC

GGGCCCCGTGGCTCCGTCATCGACCCGCTGGCGCCGCGTTGCGCACCGCGCCAGCGG

CCTGAGCGCGCGGCATCCGCGTGCTCGACAACATCACCTTGCCCTGCGCAGCCTGGTG

TGGCCACGCCAGCCGAGGGAAGCGTAAAAGCCAGCATGGGCCCGTCCGTTTCACTCC

CCTTTGCGCTGCATCCTCGCCTCATCTCCTCCCCCACACCCTATCGCCTACGACGCGCT

GCGGCTACCTGCGCCCCTGCTTCCGCCGCCTCCCTCGTTGGCCGCACGCGGCTACTCC

AGACGCCACCCTCTCCCTCCACGCCCCCACCTCTCTCCCGTCCGCC

>*PhuHsp20-18.9*

NNNNNNNNNNNNNNNNNNNNNNNNNNNNNNNNNNNNNNNNNNNNNNNNNNNNNNNNNNNN

NNNNNNNNNNNNNNNNNNNNNNNNNNNNNNNNNNNNNNNNNNNNNNNNNNNNNNNNNNNN

NNNNNNNNNNNNNNNNNNNNNNNNNNNNNNNNNNNNNNNNNNNNNNNNNNNNNNNNNNNN

NCCGCTCGCCACCGCCACCACCGTCGCGGCGGCCACGTGCACGCACCGCAATACGCC

GTAGCACGCGAGGGGGCCCTCGAGGGCGGGCAGCAGGCTGTCGCGGTACGACGCCCC

GCCGACGGCCGAGGCGGCGACGGTGGCGGCGGCGGCGGGGCCGCCCGCACGCACCG

CCGTGGCGGCGGCGGCATAGGCGAGGCGGAGGACGCCCGCGGGGGCGGCGATGCAC

AGGGCGGGCACGAGCAGGTGCCCGGCGGCGGCGGCCGCGGTCGCTGCGAGGAGCGC

GGGGGGGGGGGGGCGCCGCCAGGCGCGCCGCCGCCGCGCGGGCGGCGGTGGTGGC

GCCGGCGACGGCGGCGGTGAGGGGGGGGGGGACGCCCCCAATGGCAAGGGGGGGGG

GCGCCTCGGCCGTCGGGGGTAGGGGGGGGGGGGGGGGGGGCGCCACACGGCGCCG

CCCGCCGCGCGTGCCGCCAGCCACCGGGCCAACCGCCGCGGCGGCGGCGCCTCGTTG

GCGTTACTCCCGAGGCTGCCACAAAGGCGGTCTCCATGAAGGCGGCGGGCAGGCGCA

AAGGCAGAGGGAGGGGGGAGGTCGGCGCCGCGTGGGCGCCGCCGACGCGGACGGGC

GCCGCCGGTCCGAGCGCGCGGGGTCGGGGTCGTTGCGCCAGCGGCGCGACGGTCTCT

GCGTGGAGACGAGGGTGCGCTTTGTTGCAGAGGACCCGCCCAGCACCACTCGGTGTG

CTGGGGGGGTCCTCGATGAAGCAGAACCTCGTCTCTGATTGGCCAATTCGTGAACAGG

TCACGCTTTGGTCGAGCTCATGGAATTCAACCGTTGTACAGAGTCACAGGAGAGCCAT

TAACAGGAAGCTCAGCCAGATCAGGAAGAATCTCGCTGAGAGATTGTTCCAGGCCCTT

GGTCGAGCTTTCCAGACGCCTCCGGACGCTTCTTGACACGACCAAGACCGACCAGCG

CCGCTGCCTCGTCCGCCTGCCCCCTTCCCACACGCACTGCAGTACCCGCTGTGGGCGT

CTGCTTGCCGCCCGGCCGTGCTGTCCTGGCCTGCGCCACCGCCCTTGCTGTCGCGCCC

TGTTCGCCTGACCGTGTGGCTCCCGACTGGACAAGATGGCCGGTGGGCGCCTATATAAT

CCCTGACCGGAGTCGCTCCCGGCCCGCCAGCCCGACTTGCCATCGACACCAACACACC

CCACTCACCTGCTCACCGACTCACCCGCACACCCGCACACCTGCACACCTGTACATCT

GCACACCTACGACCAAGGGCATCAACCGACCAAGACCATCCACACGCCCACCCGCAC

CTCCCCCCTCCCCCTCCTCCCCGCG

>*PhuHsp20-20.5*

AACCGAGCATTGTGCAAGTGGCACCGAAACCGACAGTTTGTGGCGTCGACTAGTCAC

CGAAAGTGCAGCTTTTGAGAAAAGTGATGCCATCCAGGACGACAGATTGGTTCGATTC

ACTGGATCAGCCAATGACTGCACTTCCCTCCAAACTGGGCGGTTTCAAAACAGCTCCC

GAGAGTGTCAACGGCAACGACATCTGACCTGTTGATATCTCGCGACGAGCCTTCCGCA

CAATGCGGATCGTGCCAGTGGCGACGCCCGCTCTCCAGTTTCCCCGCCCAGCCGCTTA

CCGCTTCTTCTGTGGCCCGATCCAATCCGTTTACAATCGACAGGACTGCGCACGAGGA

GATCGGATTCTTCCCAGGGGCCTAGTTCCAATGAGGTGCCCGATTTATGTCGTGGCCGT

GCCAGGTGTCGGCCCGTTCGTCCCTTTGAATGCCTCTTTCTTCGACGTTGTCATCGGTT

TCGACGATTATTAGCGGCATATCTGGCGCTTGACGAGGACGTGGCAATCTCACCGCGAC

TGTTGGGCACACTTTCCGGACAACTTTTCGGTGGTTTCGTTTCATTAAGCGCTGATCGA

GATCCCTTAGCGCTCCTTCGCTTTCATTCGGATTTGGACTACCAGAATGGGGCTACACA

TAACCCAGCAATGACGACCCCATTCAGACCGTCTCGTGGCTCGTTTCAGACAGCTCGA

TGCCCAACTACATCGCGTCTGGCCCCAAACAGGGCCCCCAGAATGGGCTCTCCATTCA

GCATGCCACACGTTGCGCCTCTAGATTCCCCAATTGGAAGATGGGATCGACGTTGCAG

GGTTTGCTCTCACGCCGGCCGTCGGTTGTCTGATCTTACAGTCGTTATGCGTTTTCATG

ACAGGGGTGCTTCAAAAAAGTAGCACCCTCTTTCAGTCCGAGATCGCGAGAGATCGCG

GTCCTGAGGCTCTAGGGGCCATTCACTTGTAGAGCATGCTCTCCAAGTGAATGGCACAT

AAGCGATTAATGACTGTACTTCAAGTCGTACCCGATCTTGAGGCCCCATTATGTGCAAA

GCTGTTTTCACATCAGCGGGGACTTCTTTTCAAGTTTAGCCAATCCACCCGGAAGCAT

CCAGCCGTTTCTTGACTGTTCTGGAGCTCTCCTTTGATCGACGGCTCACTGCTGTCCAT
CATCCTGCCCCAACCACACCGTGCTCTTCCATCGCCGCGACCCATGACGATGGCAAGG
GCGCTCGCGACGCCCAGGTATGGAGGCTGATGGATGGTACTGATCAGGCACCGTAGCT
GCCTACAAATGCAGTGCTGCCGAGCGCCTCTACTCAGCGCTTTCCTTGTTTTCTCATCC
TCCCCTCTCTCCCACCCTTCGCCACTCCCGCCCCCTCTCCTGGCCGTGACGCGGAACC
ACTTGGCCACCTCAAGCATCATACCCCTCCTGTGTCGACATTCAAGAGCCAAGGTCATC
CCTCTCTTCTTTTCCCTCCCACTCTCGCTGCCCGTCCCC

>*PhuHsp20-37.5*

CGCTGATGCGCGCCGGCGGCGGCATGCCCGGCGTAGACGAGGAGGACCCTGACGGCA
TTGTCGTCACGCCGCTCATGTCGTCGCGCAACAAGCGCCCGCGCCTGGACGGGGGAT
GAGCGGCGCCAGCGGCATGGACGAGCCGGTCCTCGCAGGGCAGCTGCCGATTGGCGG
CGGTGCCGGCAGCGCCGGCGGCGGCGCCGGCAGCGGCGCGGGGGGGGGCGCCCTCG
GCGACCCGCCGTCGCCGTGGGACCACCTGCTGCGGATTGAAAAGGAGCGCAACGCCG
TGGAGCGACAACGGTTGGAGCGGGAGGCAGCGCGGCTCGAGGTGGAGGCCGGCCGC
CTGCGCGCCGCGTCGATTGCCGACGCGGCGCGGCTGGCGGCGGAGAAGGAGGACCGC
GCCGCGTCGCGGCAGCAGATGACGCAGCTGATGGCGCTCGTGTCGGACCTGGCCAAG
CGCCGCGACGCGTGAGGCGGCGTGCCAAGGGCGCGACGTGGGCGGGTCTGTCGAGG
CGGGGGTGCGGACGGTATTAGGGGCGGGGGTGGGGCTGAGGTGAGGGCGTGAGGGG
GGCAAGGTGAGGATGGTTTCGACTTTGTTGTGCATCAGTGATGCTTGCGACTATGTTTG
TTTTTGTTTTGTGTTGGTGCCGAGCTTGGTGGGGGTGCGCGCTTGTAATGAACGCTTGT
TTTTCTTGTTGCTCATCGTGCACACGGCGGCAGGAGGGCATCCTGTGGCCAGACACTG
TCCAAGCAGCCGCTGACTGACACTCGCAACAGGCGGGGGCGGAAACAAGAGGGTGC
GGGCCCGCGGCGCTGTTCGGGAATCGGATTCGACGCGTGCGCCGCGGCGGTGCCGGG
CGCCCCCCAAAAAAGCGCGGGCCGACCGCCAAGCGCCGCCTGCCGTTGCAGATCGAG
CGAAAACGGGGGCGACGGCCGCCATTGTCCGCCACGCACCGGCCGTGTCGTATATGTT
GTGCGGGCTTGTGGGTCCATGTCGACCACCCCTCGTGTCTCGGCGGCGGGTGCGTGTG
TGGCTCGCGGGCGGCGGGCTTGTGGGTGGCCGACGCTTGCACCCGCCAGCCACACGC
TTGAGGGTCACGGCGCGGGCGGTGGTGGCGGTTGGGACCGAATTGGCTCTCCGGCCT
CAATTTGTCGACGCTTGGCTCCGAGCGCCACTGTCGCGAAGATTGCTCAAGTAACGTG
GGTAGTTGGACGCGCGAGGAGCGGCGAGGTGGCGCACTCCAGCTTCCAGCCGCCCAC

CGGCTGCCGCCGTTTGCCTGTTCGGCGCCGCCAGCAATGCGGTCGCCATCACGCACGG

CCGCGCCCTATGGTTGTCGGTAGGCCCTCCAGTGCGTGGAACCCTCGCGGATGTTCCA

ATGGCTGCCGCCGGCTGTGACGGACACCGCTCCGCCGTCGCCATGACAGCCCCAGCGT

CTTCGTCCGCTGCCGCGGCAGCGGTCGCCCGTCCCACATGATAAGGACGAGCCGGCAG

CCCGCCGTTG

>*PhuHsp20-19.0*

CGTTGCGCCAGCGGCGCGACGGTCTGCGTGGAGACGAGGGTGCGCTTTGCAGAGGAC

CCGCCCAGCACCACTCGGTGTACAGTACGTACCCAATATTACTTGTCCACCCCTATCCG

TGGCCAATGCGCACGGTTGCTGAGACCGGCCCCATACAAACTGCGCTTGCTGCTGCCG

CACGACAAAGGAGCCTTTGTCGGAGAAGAAGACCAACACCCAGTGGTCGGTCCCCGA

CCCGCGGTTCCTCGTCCACGAGGCGTGTGTCCTTGTTGGCAGACGAGGAGAACTTTCT

TGGAAGGAGAATGAGATGACAATCCAAGGCCCTTGACGGCCTTCTGCATCGTTTGGAA

AGACAAGTGGGGGGGAGAGGGCCCTCCAGCAGTGTTGGTGCTCTCGGCATCGTGCTAT

TCATTATTTTCTGCCAGACTGGCGAAGGGGGCGTCCTGCTCCGTCAGACGGAGCCCGG

GCAGCTCGCATTCACTCACCACCTTTGTCCTGCTCCTGTTTACATTGTCATCCAGCCCG

CCATACCCCCGAGTGGCGGTAGCGACGTAAGCATCCGTTCTTCGATTGTGGCTGATGGA

CGTTGCCATGTCGCCGTTGAGCTGTTTGTTGGCGACCATCTCCTTGATCGCGCCTACCT

GCAGAGGGGTGATGGGGGTCCTTCGATATCCCGCCATGATGGTTCTGGTGAGTTTCTAT

CCGCTGGTGACAAGCTGGGGGCGTTTCTTCGTAGGTCACTGGGTGAACGCTTCACGCT

CAAGTCCCCAGAATGTGGCAGATCAGGGCCAGAGAGGCCGATTATCCAATCCCATTGA

CGACCGCAATCGCCTTTCCAGGGTGGGCAAATAATATTGGGATCGTACTGTACACCCGT

GTGCTGGGGGGGTCCTCCATGAAGCAGAACCTCGTCTCTGATTGGCCAATTCGTGAAC

AGGTCACGCTTTGGTCGAGCTCATTGAATTCAACCGTTGTACTGTACAGAGTCACAGG

AGAGCCATTAACAGGAAGCTCAGCCAGATCAGGAAGAATCTCGCTCAGAGATTGTTCC

AGGGCCTTGGTCGAGCTTTCCAGACGCCTCCGGACGCTTCTCGACACGACCAAGACC

ACGCACCGCTGCCTCGTCCGCCTACCCCCTTCCCACACGCACTGCCAGACCTGCAGTA

CCCGCTGTGGGCGTCTGCTTGCCGCCCGGCCGTGCTGTCCTGGCCTGCGCCACCGCCC

TTGCTGTCGCGCCCTGTTCGCCTGACCGTGTGGCTCCCGACTGGACAAGATGGCCGGT

GGGCGCCTATATAATCCCTGACCGGAGTCGCTCCCGGCCCGCCAGCCCGACTTCCCATC

GACACCAACACACCCCACTCACCTGCTCACCGACTCACCCGCACACCCGCACACCCG

CACACCTGCACACCTGCACATCTGCACACCTACGACCAAGGGCATCAACCGACCAAG

ACCATCCACACGCCCACCCGCACCTCCCCCCTCCCCCTCCTCCCCGCG

>*PhuHsp20-18.8*

TGCCGGTCGGAGTCGATCAAACTCGGGACTGCACTGTGGGTGTGCTCACCAACCGAC

CGGGAACCGCCGACCGACGGCAAGATATACACTGCCTCGGCGGGACGAGCGCCGCCG

CCGTCGTGGCGGCGGGCCACTACAACTCCGAGACAGCCCATTGGCAACAGGCAACAG

CGGAGAGGCTGCAGTGACCACACCGACCGCCACCTCCTACGGCGAACAGCGCACGCG

TGGCAAGTTACACAGGGCAAAAACGACCCGTCCGCCGCCCGCGCGCGGTGCATGGAC

GTCAACCAAAAAAATGTCATGCGGTGGCCGGCCCACGTGGTGGCCTAACCGCGGCGC

GCTCGATAGCGTGGCGCGTGGGGGACTCCCCGCACCACCCCACACAAGCTGTCAGTG

CAGCACAGTTGGCGGCCGCAGCCGTGACGGGGAAAGGAGCCAATCTCCGCGCGCCAG

GCACCACTACGCACGACATGCGGCACCGGTGCCACTTCACCATCCCTGCAACGCACTG

CAAGCGCTTTACCTACGCCCCGCCACTCACAGGCACCGACGGCGGCCATGGCGTGACC

CACTTCGGGGCGCCTGGGACCATCCCCGGGGCGCCATCCAAAGGTGGGGCCTTGCCTA

CCTTCTCTCGGCCGGAAGCACATTGTACAGTAGTGTGGGAAGCTGCACGTGCGTAAGA

CGCGCGCACCTCGGGCCGGCTGCTCGAACGGGGGCTGCTTTGGTGGCGGCCCAAGCA

GCAGCGCATGCAGTAGCGACGGCACGCCACGTTACCAAAGCAGTCAAGTCCACACCA

CCACAGCTGTGGGGTCCCTCAGCTGTGGTCTCGACGACGACACCGCCACAAAGATCA

GTCCGTAGCCACGTCCGGCTTCATGGTGCTGGGGGAGGAAGCGCCCGAGCACGAGGC

TAATAATAAAGACGCCCAGTCGCGACCACGACTGTCAAGCACAAACTACCAGGAGAG

AGTCCGAAAATACTGCAATGTGTACACAAAATACGATAAGTGACTACAATAATTCCATG

TTCAATTTCAGATGTTCTGGAGAGTTCCACGGATGCATGCGTGTTCCACCAAGATTGGC

TGGAACGCGGATGCAGCCGAGTGTCGGTTCCTTGCGATCCGGCCCAAGCGTGAACTCT

S-type

TCTCGGTCTTTCGAGGTGGTTCCTCGCCACCTGGTACGCACGCATGCCCTGCTCTGTTT

GCGGTGGAAGAAACTCCGAAGCCTCATGGTGCCACGCTGCGCTCCGCTTCTACGCGAC

CCCGATTATGTGCCGTGCCTGCCTGCAGCGAAGGGCCGGCCACCGAGCGCTGCCCGTG

CCGTTCCAGGCTCTATAAAAACGCATCAGCCGTGCGACACCAGGCTTTTCGCCGGCTG

GCACCCACCCAACCAACACACACACACACCCCACCATCGTCTCACCCGCCCTAGGTTC

TCACACCTCCTACCCTCCACGCCTTCACCCCGCTCCCCACCCTCACACCAAGTCCTCGC

C

>*PhuHsp20-19.3*

CGGAACATGGCCATAAACAAACGTGTTGAATTGCATCAGAGGATGCGGATTGCAGGCC

GTGGACAAGACGAAAGGAGACAACGTACATCAGCTGTCTCTCTGACTTCAACGGTCA

CTCTGGTCAGCTGCAAGAGCTTCCCACCGGTGGGCTGCCGCCGCTCCAGCCGCAGTAT

CTGGCAAGCCGCAACAAGACCAGGTTGGTGCAACCGAGGCAGAGCAGTCGGGCGGG

CGAAGCACAGCGCCAGTCAGTCATCACGACACCGACATCACTACACCGCACCCATTCC

ATGTTGCAGCACATGGCCAGAGAAAGGGCACCAAGAAGTTCACGCTACGCGGTCAAC

GAACAGCAGTCATGGCGCACAGAAGCAGGGCTCGCCATTACGTACAGCAGTGTCGTG

GCCGCCGCCCGCATCGGGCAGTTGCACGTAGTGCCCAACCGAGCCGCCTCGTGTGTGC

ACCGGCGCAGCGTCTCCGCCTGGCGAACCGTCTGCTGAGCGGCGTAGATGCGGTGGG

GGAAGGTGCTCTGGCCCCAACTTGCCGGCCACGGTCGTCTCAAAGGCGACGACGTCA

TAGCCGAGGCGGGCCACCTCCCAGAGAGCGCCGTCACCATGGCGCTCGTGCAGGGAA

GCCGGGACGCTCAGGTCATGGAACATGATCGAGCAAGCAAAAGGAAATCCCTTCTATG

GCCGGGGTGACTGACAAGGAGGCCAACGATCCCATAGCTCAACCGCGCGAACTGCTG

GACGGGCGATGGCGGCCGTGACGGTGACGACTTGACCGCCGCGAGCGAAGCGGGATG

GATCAACGACGACGAGTTGAATGACAGAAGCCTCTGTGACAATTAGATGCACAATACA

CCAGACAGTCTAAAAATCTAGATGGAAGAGGGCTGAGGTGAGAAAGAAACTCCAGCC

P-type

CAGAATATTATTGTGAATCGTAGACTGAATGGCCACGTGGTTCTGGAAACTTCCACCGAT

GCAAATGCTGGTTCCCGCAGATCTGCAGATCCGCGTCGCCTGTGCGTGCACTCTTCTCG

GTCTTTCGAGGCGCTTACTCTGCAGCTGGAGGTCCGCTGCAGCGGCTGGGGGCACACC

CGTTCCCTGGGGGCACACCCGTTCCCTGGGGGCACACCCGTTCCCTGACGTGTGCGCT

AGTTCCGTCTCGTTTGCGGTGGAAGACCACGGAAGCCCCGAGGCACCCCACAGCGCC

CTCGGCAGCCACGCGGCGCCCGTTCCGAGCTGCATCCGCCTGGCGATGCCAATGGCAG

GCCTCGTCGCGCCGCGCCTGCAGTTGCCTGCCCGGTGCGCGGCCATCACCGCCGCCGA

CTGCTCTATAAAGGAGCAACAACCGCGCACGTCTGTCCAGTGGCTCGACACGGACCA

ACCCAACATACACACGCCACCGACGTCTCGCCCCCCTTCGAACCCACCCTTCCTCACC

TACCACGGCCTTCCACGCTTCGCGCACCCCACCCACCACCCCCCCCTCCCGCGCCCCT

GCC

>*PhuHsp20-24.4*

GCCGCTTGCGGGCATGCGCGCCGCGCCCACGTCCAGAGGATGGAGGTGGAGAAGAGG

AGGACCGCCTACTCGGCAGAGTTGGGCGTACACGACCACGGGTAGGGCTTGGAGAGA
TGCGCGAGCGTCCAGACGGTGACCGACGGAGAGAGGGCAGGGGGCTACGGGGGCGG
CTCGCCCCAGAGGGGAGGTCTGGCCGAGGTTGTGGGCCGGGAGACATAGCCCGGACA
GAGCGTTTCGCACTCGCGAGCGGGGGGGAGAACGGCCGCGAGCGGGGGGGGGGGGG
GGGGGGAGGGGGGGACGATCGCTTTTGGCGTGCCATGTCGTCGGCTCGTGGGAGAGC
TAGACCGGCGGGCGCTGCGGCGTGACGCGGTGCCGCGGGCAGACATTGCGGGGACGC
GGGTTGCACGGGTCGGTGCGCGGGCGGTGATGGGGCGGAACCCGACCGACGCTGCAT
GGAGAGCGGTGTGGGATTATATGGCGCCATGGCCACCCTCCACACCGCAAGCAGCGGT
GGCCCGCCCCATCCTGCCAAGCGGTGTTGTGGGGCCCTGTCCAGCCTTGTCGAACAAC
GCGATTGCAGGTGGTGTGGTGCGGGGGGGGTGGGATGGGGGCAAAGAAGCAACAATG
TGCGACTGTGGGGCTGATCCGCATGTCCTCCATGTAGGATCTCAACATCTCAAACACGA
AGCAAAATTTTGCTTCGGGCGAACGCTGACGAGCTCTGAGAGCGCGTGCTCCGGAGC
AGCTGCCCAAGGCCCTGCTGGGGAATGCCTTGGGCCATTGGATTTACTGCTTCGCGAG
CACAATACCATTGCCTGGCCTGGGGTGTCGGTGGTGATGACGACATGCATGGATGACA
CCCGCTCTGGGATGCCATGAGGCGGCCCCGCTCTCCCCGCGCCCGCATCCTCCCGCAC
CCGCCCCTCCCCGCTCCCCCCGCCCCCCCGCCCTCCTCCTATCGCGCCCCAGCCTCCTG
TGGCATCCAGGTGCTCCCTATGGCCCCCTCCGCCAGGGTGGCGGTCCGCGGGGGCGAC
AGCCGCAGGCGGCGGCGGCGGCCGAACGGGCTGCAGCCCGCCGCTGGAGGCACCCC
CCCCGCCCCCCTAGCTTCCCACTCCCCCCGCGCCACCCCCCCTCCCTGCCTTTTCCGGC
TTCCTGCCGCTTCCCTTCGGCCCCATCTGCCATGCTGGCGGTGTGTGCTGACGGTGGCG
GCAGCCAGCGGCGGTTGTCGTGCGGGCTGCCGCCCGCCCCTCGGGGCACCATTCGCG
CCCGCCCCGCGCCGCTGCCCCCCTGCCCCCCGAGGCTCCCGGAAGTGCTTAAATAGGG
TGCGTCTGGCATCGTCCGGGGCCGGGTCCCTCACCGCCAACACACCCGGTCCTATCAC
CCCACCGCCGACGCTGCCCCCACCACACACCTGCGAGCGGCGCGCCTCTTGCACTCCC
CCACCGGCGACCGACCACCCGTCCTCCACACACGCCCGGCGCACCCGTCCCCGCCAC
CGTC

>*PhuHsp20-28.5*

CAATCTCAAACGCCACCCCCTTTTCCGCGGCCACATGCGACTGGGGCCGCTTGACAAA
GAAGGGCAGCCGCGCAGAGAGGTCCAGCGAGAGGATGTCAATGTCAAATTGCATGCA
CTGCTGCATCAGCTTCTCCGTCGCCGGCCGGACTGCAACAATGTCGTACTGGGCCAAG

ATGGGGGACGCGAGGTTGGTCAACTACAGGACGTGGTGGACAATGGGCAACCACGAG
AGACCAGGCACAAGAGGCGTCGGTGGCACGAGAGAGGGCTACACATGGGATGGTGA
GAAACGGAACATGGCCATAAACAAACGTGTTGGAATGCATCAGAGGATGCGGATTGCA
GGCCGTGGACACGACAAAAGAAGACAACGTACATCAGCTGTCTCTCTGACTTCAACG
GTCACTCTGGTCAGCTGCAAGAGCTTCCCACCCGTGGGCTGCCGCCGCTCCAGCCGCA
GTATCTGGCAAGCCGCAACAAGACCAGGTTGGTGCAACCGAGGCAGAGCAGTCGGGC
AGGTGGACCACAGCGCCAGTCAGTCATCACGACACCGACATCACTACACCGAACCCAT
TCCATGTTGCAGCACATGGCCAGAGAAAGGGCACCAAGAAGTTCACGCTAGGCGGTC
AACGAGCAGCAGTCATGGCGCACAGAAGCAGGGCTCGCCATTACGTACAGCAGTGTC
GTGGCCGCCGCCCGCATCGGGCAGTTGCACGTAGTGCCCAACCGAGCCGCTCCGTGTG
TGCACCGGCGCAGCGTCTCCGCCTGGCGAACCGTCCGCTGAGCGGCGTAGATGTGGTG
GGGGAAGGTGCTCTGGCCCCAACTTGCCGGCCACGGTCGTCTCAAAGGCGACGACGT
CATAGCCTAGGCGGGCCACCTCCCAGAGAACGCCGTCACCATGGCGCTCGTGCAGGG
AAGCCGGGACGCTCAGGTCATGGAACATGATCGAGCAAGCAAAAGAAAATCCCTTCT
ATGGCCAGGGTGACTGACAAGGAGGCCAACGATCCCATAGCTCAACCGCGCAAACTG
CTGGACGGGCGATGGCGGCCGTGACGGTGACGCCTTGACCGCCACGAGCGCAGCGGG
ATGGATCAACGACGAGATGAATGACAGAAGGCTCTGTGACAATTAGATGCACAATACA
CCAGACAGTCTAAAATTCTGGGTGAAAATAAGGCTGAGGTGAGAAAGAAACTCCAGC
CTAGACTATTGTTGTGGATCATAGCAATGGCCACGTGTTCTAGAGGCTTCTAGCGATGC
AAGTGCTGGTTCCGGCAGATCTGCAGATCCGCATGACCTGCGCGTGCACTCTTCTCGG
TCTCTCGAGGCACTTCCTCTCCAGCTGCAGGTCCGCTGCAGCGGCTGGGGGCACACCC
GTTCCCTGACGTGTGCGCTAGTTCCGTCTCGTTTGCAGTGGAAGACCTCGGAAGCCCC
GAGGCACCCCACAGCGCGCTCGGCAGCCACGAGGCGCCTGTTTCGAGCTGCATCCGC
CTGGCG

>*PhuHsp20-20.1*

ACGTATACACTTCACCCTTTCAGAAAAAGAGAGAGACGGAGAAGCATCTATGGCCCTG
CTAGCAGTCGCAATGTGTGGAGTGAGGAACGATTCCTGTTCTCGCAAAGACAAATATG
CAAAGACCTCGCATGGGATGGCCTCTGAGAGCCTGTCAAGGGGGGTCGATCTGTTTTT
TGCACAGGAATTCTGTTTCTTGAATCAGGAACACGGGCCTCCAAGAGGCTCAAAACA
AGCAAAAACAGACGTGCTTTTGACGATGGTTGTGGGCTCGCTGTGATCGAAATTTTCT

AGTTTCTGAATGACTGTGAAAAAACCAAGGCTGATCGTGGGCCCACACGTTGCTCGCT

TAAGCGTACTATCTTCTATACTGTTTCGAATCATTCGACCAATGAGAGAGCATAGAAGA

ATGGGGTCGGCTTCGGACCGACCCCATTTCCGGTCGGGCTCGGTCCGGCACGGGCCCC

ATTTCTGAAAGCAACATATGCGTTGTACTTACGTCATTTGATTTGGTTGAAATGAAGCCT

AAATGGGGTCGGAGACGTTTATGCGGGAGCGTTCCGGCCCCATTTCCTTAATAATCAGA

CACGTTTGCGCCACGTTGCGCCACGTTTCTCAAATATGACTTGGGGCCGACGTTGCAG

GGTTTGCTCTGATTGCACCCGCCAAGAGCTTTCCAGTGGCCTGCCGACGTGGACACCC

GATCACACACCGCGAGCCATCTTTCCGAGATGGTCGTTGGCCGGGCGCCGCCATAGGT

CGCGGAGCATCGGATGTCCCGTGCTTCCACCGCCGGTCAGGGGCTGGTAGCCGCCAGA

CAGCTGCCGCTGTATGCGATGCCGTGGCGGTCTGTGGCGGTATGTTCCTCTCTCCAGCG

TGATGGCGCCTCCCCCCCCCCCCCCGCGTTGGATGCCGCGCGACGCCTGGTTGGAGGC

GTTGGCGCTACCGCCTCCGTGCCGCCGCCTCGCGTGGCCGCCGCTGGGTGGGGGGTTG

CTCATGCATCGGCACCTGCGGGTGCCGCCTTTCTCCTGTGCCCTGAGCACCTACCCCCG

TTGCTTGGACAATACAAAAGGCCACCACCTACCTCGGCCTCTTGTCCATTCTTCCCTTC

ATCCGTCTTTCCACGTCTCGCCCACCCACCCGCCCGCGCCGACCACCCCACACCCCCT

GCTTCCGACACGACGACCGTCGACTTGCGCCACGGACGCGCACGGTACCGCCCCCCTC

TTCCCCCCCACCCCCCGAACCATGGCGTTTGTCTTTACCGCCGTCGGCGTCGGCGCCCC

CGCCGCCCGCACGTTGGGCGGCCCCGCGACACGAACCGCGCGCCCCGTCTTGGCAAC

CACCCCCGCCATGGCCTATGGCGGCCGCGGCTTCCCCTCTGCCGACGCGCAGCGGGAG

GCTTGGCGGCGGCGCAATGCGGCGCGCGCCGGCGCGGGCGGCCCGCCGCCGCCGCCA

CAGCAGCAGTGCGCGCCCGGCCGGGCGCCGACGCAGGAGGAGCTG
